# Microheterogeneity and Individual Differences of Human Urinary *N*-Glycome under Normal Physiological Conditions

**DOI:** 10.3390/biom13050756

**Published:** 2023-04-27

**Authors:** Hend Zedan, Kousuke Morimura, Amr Elguoshy, Tadashi Yamamoto, Shunji Natsuka

**Affiliations:** 1 Department of Life and Food Sciences, Graduate School of Science and Technology, Niigata University, Niigata 950-2181, Japan; 2 Biofluid Biomarker Center, Niigata University, Niigata 950-2181, Japan

**Keywords:** HPLC, multistage mass spectrometry, *N*-glycans, urine, serum

## Abstract

Urine is considered an outstanding biological fluid for biomarker discovery, reflecting both systemic and urogenital physiology. However, analyzing the *N*-glycome in urine in detail has been challenging due to the low abundance of glycans attached to glycoproteins compared to free oligosaccharides. Therefore, this study aims to thoroughly analyze urinary *N*-glycome using LC-MS/MS. The *N*-glycans were released using hydrazine and labeled with 2-aminopyridine (PA), followed by anion-exchange fractionation before LC-MS/MS analysis. A total of 109 *N*-glycans were identified and quantified, of which 58 were identified and quantified repeatedly in at least 80% of samples and accounted for approximately 85% of the total urinary glycome signal. Interestingly, a comparison between urine and serum *N*-glycome revealed that approximately 50% of the urinary glycome could originate from the kidney and urinary tract, where they were exclusively identified in urine, while the remaining 50% were common in both. Additionally, a correlation was found between age/sex and the relative abundances of urinary *N*-glycome, with more age-related changes observed in women than men. The results of this study provide a reference for human urine *N*-glycome profiling and structural annotations.

## 1. Introduction

*N*-glycosylation is considered one of the most common co- and post-translational protein modifications [1,2]. Approximately 50% of human proteins are likely to be *N*-glycosylated, with some estimations approaching 70% [3]. Unlike nucleic acid and protein synthesis, *N*-glycosylation is not a template-driven process, as diverse glycosyltransferase and glycosidase enzymes control the attachment of the oligosaccharides to some asparagine residues of the protein backbone [4]. *N*-glycans are involved in numerous physiological processes, including protein folding, cell signaling, immune reactions, host–pathogen interactions, protein trafficking, and so forth [5]. The variation of the *N*-glycome profile reflects the physiological and pathological status of cells and may be associated with different diseases’ onset and progression [5].

In the context of biomarker discovery, different *N*-glycome studies are focused on building a baseline reference from human tissues or biofluids under normal physiological conditions, which can be used for the comparison with future *N*-glycome studies under disease conditions. For example, Kaijie Xiao et al. identified 214 distinct *N*-glycan structures with a spectrum-level false discovery rate ≤ 1% from human normal liver LO2 seed cells [6]. They reported that 37% of identified *N*-glycans was fucosylated, 13% was sialylated, 32% was sialylated-fucosylated, and only 4% was oligomannose-containing *N*-glycans. Additionally, the glycome profiles of IgG-depleted and nondepleted plasma samples were demonstrated under normal physiological conditions, and they identified 50 and 106 independent glycan ions before and after IgG-depletion, respectively [7].

In urine, Haiying Li et al. identified 116 *N*-glycan compositions. Additionally, they performed quantitative comparisons of the most repetitive 46 glycan compositions between different age and sex groups. They showed significant differences between the adult male and female cohorts [8].

However, the previous studies investigated urinary *N*-glycome under normal physiological conditions in the context of biomarker discovery. They generally identified glycans at the composition level, without assessing further structural aspects that may differentiate glycan isomers with the same composition.

Of note, only using MS-MS/MS data is not sufficient for this purpose, and further dimensions might be required. Research reported that liquid chromatography (LC)-MS techniques, such as hydrophilic interaction-LC (HILIC) [9], porous graphitized carbon (PGC)-LC [10], and reversed-phase (RP)-LC [11,12] are often more suitable approaches for this purpose.

However, the isomeric separation of PA-*N*-glycans on a C18 column outperforms other LC methods in discriminating the glycan isomers with different branching structures based on their elution time, as pyridylamino residue (PA) is small and less hydrophobic than other labeling methods, such as 2-AB or 2-AA, which makes the contribution of the glycan itself to the retention relatively high and results in better separation of different species and deducing more structural information on the glycan [13,14]. Thus, to investigate the structural features of the human *N*-glycome, and discriminate among glycan isoforms under normal physiological conditions in urine and serum, PA-Glycans were dually fractionated by anion-exchange HPLC and reversed-phase (RP)-LC before MS-MS/MS analysis.

Additionally, considering these identified structural features, we aim to investigate the variations among genders and different ages in urine. Moreover, a comparative analysis of urinary and serum glycome was conducted to distinguish between *N*-glycan structures originating from the kidney or urinary tract and those originating from the circulation system.

## 2. Materials and Methods

### 2.1. Protein Extraction and Purification from Urine Samples

Urine samples from 12 healthy individuals, including 6 males with an average age of 32 years and 6 females with an average age of 30 years (Table S1), were obtained. The urine samples were collected and immediately placed in ice boxes, followed by centrifugation at 1000 rpm for 10 min at 4 °C to eliminate debris. The samples were then stored at −20 °C for further processing. The methanol/chloroform (4:1) extraction method [15] was used to extract glycoproteins from the urine.

### 2.2. Preparation of PA-Glycans

Urinary *N*-linked glycans were liberated from glycoproteins by hydrazinolysis, according to the method described before [16,17]. Briefly, the lyophilized samples were subjected to heat at 100 °C for 12 h with anhydrous hydrazine. Repeated evaporations were applied to the samples to remove hydrazine. The released glycans were re-*N*-acetylated with acetic anhydride in a saturated sodium bicarbonate solution and then passed through a Dowex 50Wx2 (H^+^) cation exchanger (Dow Chemicals, Midland, MI, USA) to remove sodium ions. The reducing ends of the liberated glycans were tagged with the fluorophore 2-aminopyridine using reductive amination [18,19]. To analyze human serum *N*-glycans, 5 μL of pooled healthy human serum was purchased from CosmoBio (Tokyo, Japan). The method used was the same as for the urine samples.

### 2.3. Purification of PA-Glycans

To remove the excess reagents and contaminants, three consecutive purification methods were applied: water-saturated phenol/chloroform (1:1, *v*/*v*) extraction, gel filtration (1 × 8 cm, TSK-gel Toyopearl HW-40F; Tosoh, Tokyo, Japan), and solid-phase extraction with a graphite carbon cartridge (GL-Pak Carbograph 150 mg; GL Sciences, Tokyo, Japan). These methods were reported elsewhere [12,20].

### 2.4. Anion-Exchange HPLC for PA-Glycan Separation

Prior to LC-MS analysis, the samples were fractionated according to the number of negative charges using anion-exchange HPLC, in which PA-glycans were separated on a TSK-gel diethylaminoethyl (DEAE)-5PW column (0.75 × 7.5 cm; Tosoh, Tokyo, Japan) using a Waters Alliance HPLC system with a Waters 2475 fluorescence detector (Milford, MA, USA) in the methods previously reported [12].

### 2.5. LC-MS Analysis

PA-glycans were analyzed using a Dionex U3000 HPLC system and an ESI-probe coupled to a LTQ XL linear ion trap mass spectrometer (Thermo Scientific, San Jose, CA, USA). PA-glycans were separated by reversed-phase LC on an Inert-Sustain AQ-C18 column (2.1 × 150 mm) at a flow rate of 0.2 mL/min at 30 °C. Elution was performed using Eluent A (0.2% formic acid) and Eluent B (20% acetonitrile in 0.2% formic acid). The column was equilibrated with Eluent A, and 3 min after sample injection, the proportion of Eluent B was increased linearly from 0 to 25% over 100 min, and then to 100% over 5 min. The elution times of the PA-*N*-glycans by reversed-phase HPLC were standardized and converted to glucose unit values by comparison with the elution times of PA-isomaltooligosaccharides, as described before [12].

MS and MS/MS data were collected in a data-dependent mode, and the top five full MS peaks in each scan event were selected for MS/MS analysis. Collision-induced dissociation was used for MS/MS analysis. The data were recorded and analyzed using the Xcalibur 2.2 software (Thermo Fisher Scientific, Waltham, MA, USA).

### 2.6. Data Analysis

To identify the *N*-glycans, the analyzed ions were compared against an in-house glycan library [12,14] using various factors, such as precursor ion mass, elution time, glucose unit, MS2 fragment ions, and published structures. To calculate the relative abundance of each glycan in a sample, the glycan area was expressed as a percentage of the glycome area of the A2G(4)2S(6)2 glycan, which represents the most abundant glycan in both urine and serum samples. Individual glycan structures were named using the Oxford nomenclature [14,21], in which different symbols are used to represent specific features of the structure. For example, the letter A indicates the number of antennae (GlcNAc) on the trimannosyl core present, F represents fucose, B denotes the presence of a bisecting *N*-acetylglucosamine, G represents galactoses, and S indicates sialic acids.

Statistical analysis was conducted using the Wilcoxon test to compare the data, with *p* < 0.05 indicating significance. Additionally, a correlation test was used to investigate the relationship between the relative abundances of identified glycans and age, with *p* < 0.05 considered significant. All plots were created using the ggplot2 package in R. A bootstrapping technique was used to validate significantly differentiated glycans between males and females, as well as glycans significantly correlated with age. This method was also used to detect newly significant glycans that were not identified in the original data (false negatives).

## 3. Results and Discussion

### 3.1. Structure Analysis of N-Glycans

To obtain a *N*-glycome profile based on reversed-phase HPLC and mass spectrometry from urine under normal physiological conditions, hydrazinolysis was utilized to release *N*-glycans from urinary glycoproteins. These *N*-glycans were then labeled with pyridylamino residue (PA), which is a useful glycan labeling method for improving the separation ability of glycans in reversed-phase HPLC. The PA-glycans from glycoproteins in urine were separated into different fractions based on their sialylation levels—neutral(N), mono-sialylated(A1), di-sialylated(A2), tri-sialylated(A3), and tetra-sialylated(A4)—using anion-exchange HPLC (Figure S1).

Each fraction was then further separated on reversed-phase HPLC (Figure S2) and analyzed using ESI-MS.

**Figure 1:**
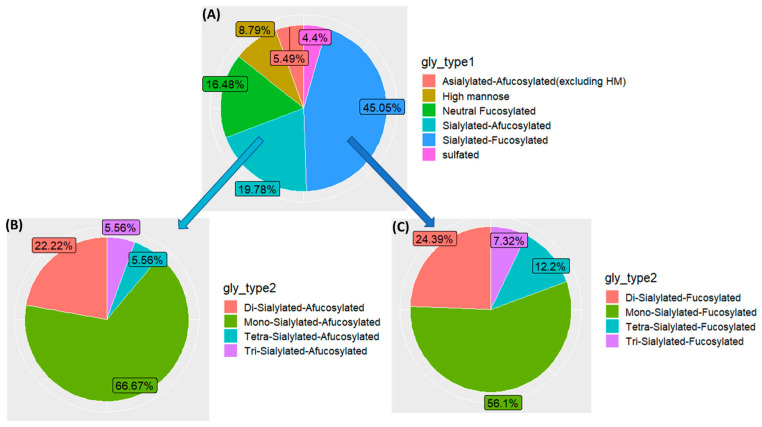
(**A**) Pie chart showing the distribution of the identified *N*-glycans through different glycan classes; (**B**,**C**) show the distribution of the sialic acid count for only sialylated glycans.

In general, mass spectrometry can differentiate between glycans based on their *m*/*z* values. As a result, we were able to identify 67 distinct compositions, which can be grouped into 6 different categories according to their primary constituents. Sialylated and fucosylated glycans made up the majority of urinary glycans (45.05%), followed by sialylated-afucosylated (19.78%), neutral fucosylated (16.48%), high mannose (HM) (8.79%), asialylated-afucosylated (excluding HM) (5.49%), and sulfated (4.4%) fractions (Figure 1A). Analysis of the sialic acid count distribution for sialylated glycans revealed that mono-sialylated glycans were the most common, representing 56.1% and 66.67% of sialylated-fucosylated and sialylated-afucosylated glycans, respectively. In contrast, tri-sialylated glycans were the least common among all sialylated glycans, representing only 7.32% and 5.56% of sialylated-fucosylated and sialylated-afucosylated glycans, respectively (Figure 1B,C).

Overall, our findings align with those of Haiying Li et al. [8]. They examined the glycome of healthy volunteers and showed that most urinary glycans were both sialylated and fucosylated, while sulfated glycans were present in only small amounts.

Earlier studies highlighted the significance of the primary constituents of the glycans identified in the normal physiological condition of the urinary glycome profile. The presence of sialic acid has been shown to be crucial for various biological functions, as the removal of sialic acid from uromodulin has been found to result in a loss of its ability to inhibit crystal aggregation and growth [22]. Moreover, it was reported that high mannose glycans in uromodulin play a significant role in the urinary defense against bacterial infections [23,24].

Since mass spectrometry alone faces challenges in unraveling the glycan structure and differentiating between glycan isomers with varying branching structures, the elution time and glucose unit were utilized to determine the structural details of glycans and distinguish between various glycan isoforms with the same mass according to the empirical additivity rule of unit contribution [12].

We applied this rule to determine how each monosaccharide contributed positively or negatively to retention on C18 columns, taking into account the monosaccharide type, position, and glycosidic linkages. With this approach, we successfully identified and quantified 109 distinct *N*-glycan structures corresponding to 67 glycan compositions from urine samples using LC-MS/MS analysis.

These identified *N*-glycans displayed a uniform elution profile across all samples, which is useful in accurately determining their structural characteristics and distinguishing between glycan isoforms.

**Figure 2:**
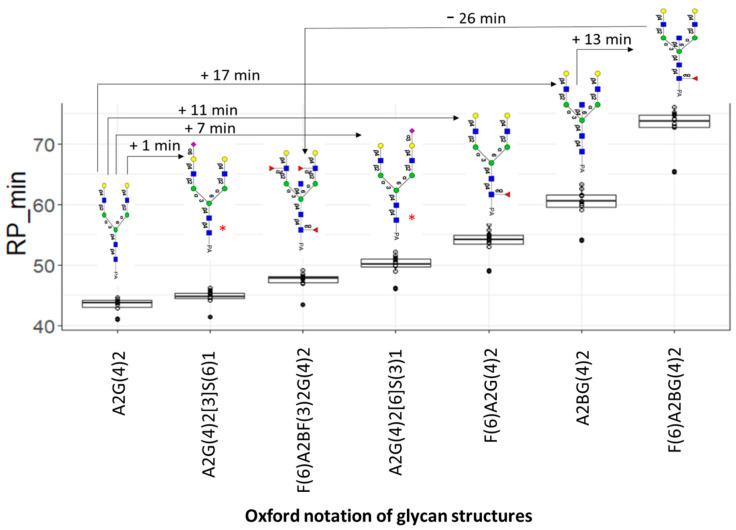
Boxplots showing the distribution of different PA-*N*-glycans elution times in minutes across samples. Line arrows indicate the time shift of the elution positions of PA-*N*-glycans by the addition of certain glycan residues on a certain position. Glycans with * in red are glycan isoforms with the same composition.

For example, we observed differences in the elution times of M7[6] and M7[3] glycans (H7Hn2PA1) in the N-fraction, attributed to the addition of a terminal mannose to α1,6 or α1,3 core mannose antennae, respectively, where the former had a tendency to elute earlier by approximately three minutes. Similarly, differences in the elution times of A2G(4)2[3]S(6)1 and A2G(4)2[3]S(3)1 glycan isoforms with the same composition (N1H5Hn4PA1) in the A1 fraction were attributed to the contribution of α2,6-NeuAc or α2,3-NeuAc, respectively, with the former eluting earlier by approximately five to six minutes (Figure 2).

Applying the empirical additivity rules by comparing the elution positions of the N-fraction PA-*N*-glycans on reversed-phase LCs suggests that the addition of core Fuc contributes positively to the retention by 10–12 min. In contrast, the addition of one α1,3-Fuc residue with bisecting on a branch made a substantial negative contribution to retention (10–15 min). Moreover, the addition of bisecting GlcNAc contributes positively to the retention by 16–17 min (Figure 2).

Interestingly, the existence of a methyl group in Fucoses made them relatively hydrophobic compared to other *N*-glycans monosaccharides. In core-fucosylated glycans, the interaction of the PA label and fucose with the C18 stationary phase is more likely to be stronger, leading to increasing their retention.

In contrast, when the fucose is positioned at the opposite side of the PA-label (on one of the antennae), the interaction with the C18 stationary phase is less strong, leading to decreasing their retention [25].

In A1 and A2 fraction glycans, the addition of α2,3-NeuAc contributed positively to the retention in the range of 6–8 min (without bisecting GlcNAc, with or without core Fuc), whereas the addition of α2,6-NeuAc attached to LacNAc on α3-Man made a smaller contribution to retention, which was less positive (0–2 min, without bisecting GlcNAc, with or without core Fuc) (Figure 2). Of note, the results obtained from applying empirical additivity rules are highly consistent with previous studies describing the separation of PA-labeled glycans with C18 columns [12,26,27].

### 3.2. The Origin of the Urinary Glycome

It was estimated that 70% of the human urinary proteome could originate from the kidney and the urinary tract, whereas the remaining 30% comes from various systemic organs and is then filtered by the glomerulus [28]. However, the origin of the glycans present in urine has never been investigated. Thus, in this section, we aim to investigate the origin of the urinary glycome. The results obtained from the serum and urinary glycome comparison showed that ~50% of the urine glycome may originate from the kidney and urinary tract, where it was absent in serum (Table S2). In agreement with our finding, Haiying Li et al. compared urinary identified glycans in their study with the published *N*-glycome of human serum/plasma, and they reported that 50% of the urine *N*-glycome was not present in the serum/plasma glycome [8]. This emphasizes the potential of using the urinary glycome as a source of new biomarkers for kidney and urinary tract diseases.

Since uromodulin is the most abundant protein in urine, and it was estimated that glycans represent ~30% of its weight [29,30], we postulated that a high portion of the identified glycans that originated from the kidney and urinary tract could be released from uromodulin. On the other hand, ~30% of serum glycans were represented uniquely in serum only (Table S3).

Most of the unique glycans in serum are sialylated *N*-glycans. These glycans may lose their terminal sialic acid residues during blood circulation through the action of neuraminidase (sialidase). Such cleavage exposes the galactose residues and generated galactose-terminated glycoproteins, known as asialoglycoproteins (ASGPRs), which can be completely taken up after binding to asialoglycoproteins receptors (ASGPRs) in the liver [31] or renal tubules [32]. These receptors might contribute to the removal of sialoglycoproteins from the serum via endocytosis.

Additionally, other less common unique glycans in serum, such as high mannose glycans, can be cleared by the mannose receptor (ManR) in the liver because they can bind to glycoproteins with oligomannose-type glycans [33].

**Figure 3:**
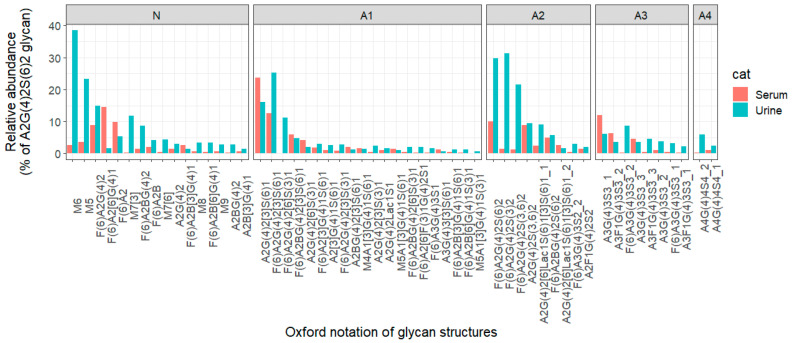
Bar plot showing the difference of relative abundances of common glycan in serum and urine as a percent of A2G(4)2S(6)2 glycan in different fractions(Neutral(N), Mono-sialylated(A1), Di-sialylated(A2), Tri-sialyated(A3), Tetra-sialylated(A4)).

The relative abundance of common glycans in urine and serum was calculated as a percent of A2G(4)2S(6)2 glycan(N2H5Hn4PA1), as it represents the most abundant glycan in urine and serum samples (Figure 3). Of note, A2G(4)2S(6)2 glycan represents 35% and 18% of the total serum and urine glycome signal, respectively.

Interestingly, the relative abundance of the majority of common glycans in both urine and serum was elevated in urine compared to serum. These urine-elevated glycans may be released from proteins that are enriched or enhanced in the kidney and urinary tract. For example, the M6 glycan was approximately 14 times more abundant in urine than in serum. Conversely, some glycans, such as F(6)A2[6]G(4)1 and F(6)A2, were found at higher relative abundances in serum than in urine, increasing by approximately 9 and 2 times, respectively. This dual contribution of shared glycans to both the serum and urine glycome may be due to incomplete or partial reabsorption (Figure 3).

In agreement with our findings, Li et al. identified the M6 composition (H6Hn2) as the 2nd highest relative abundance (9.45%) in the enriched uromodulin fraction compared with low relative abundance (0.62) in the depleted uromodulin fraction, showing that the majority of urinary M6 may be released from the enriched kidney protein (uromodulin) [34]. Oppositely, the F(6)A2 composition (H3Hn4dH1PA1) was identified in the uromodulin-enriched fraction with low relative abundance (0.02) compared to that of the uromodulin-depleted fraction (0.15), showing the possibility of release mainly from other tissue-enriched or enhanced plasma proteins [34].

### 3.3. Annotating the Structure of the Core Glycome in Urine

**Figure 4:**
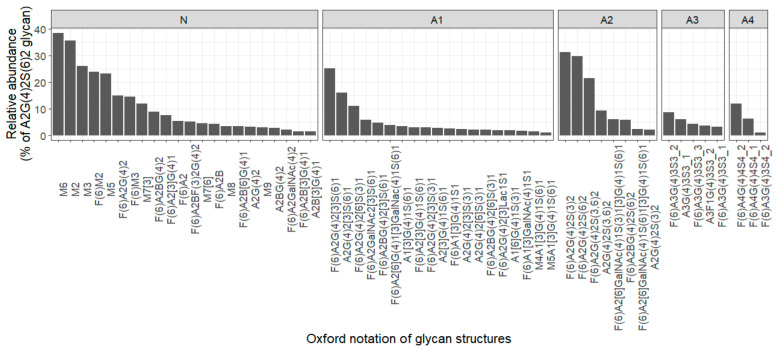
Bar plot showing the average of the relative abundances of 58 glycans in the urinary core glycome in different fractions (Neutral(N), Mono-sialylated(A1), Di-sialylated(A2), Tri-sialyated(A3), Tetra-sialylated(A4)).

Of note, 58 of all identified glycans showed a tendency to be identified and quantified in at least 80% of urine samples; we named them in this manuscript as “the urinary core glycome”, and they accounted for ~85% of the total urinary glycome amount (they are shown with a blue background in Table S2 and in Figure 4).

Investigating the distribution of the relative abundances for the glycans of core glycome across urine samples showed that A2G(4)2S(6)2 was the most abundant glycan, accounting for 18% of the total urinary glycome, and it increased more than twofold over the other most abundant glycans in the other fractions M6 and M2. Glyconnect data showed that A2G(4)2S(6)2 with a composition of N2H5Hn4PA1 could be released from the most abundant proteins in serum and the plasma-like immunoglobulins Plasminogen, Serotransferrin, and Fibrinogen (https://glyconnect.expasy.org/browser/structures/1378 (accessed on 6 Septemper 2022)).

We analyzed the structural features of the core glycome in different fractions, as they could be attributed to its precursor protein function. In the neutral fraction, 22 out of the 58 core glycans were identified and quantified. The most abundant structure was M6, followed by M2, M3, F(6)M2, and M5. Each of them has a relative abundance above 20% of the A2G(4)2S(6)2 signal. On the other hand, other glycans have a relative abundance of less than 20% of the A2G(4)2S(6)2 signal (Figure 4). However, they tend to be identified repeatedly in at least 80% of samples, indicating their crucial role in different biological activities under normal physiological conditions. As shown in Table S2, 11 neutral glycans in the core glycome were fucosylated (neutral fucosylated type), and 10 glycans (N-9-1, 9-2, 11, 12, 14, 16-1, 18, 19, 20, and 21) (Table S2) were fucosylated only on the core (α1,6-Fuc), whereas glycan (N-10) was triply fucosylated at the core and branches.

Additionally, partial structure information of the identified glycans can be deduced using MS/MS spectra, as it can validate the existence and location of certain structural features in the identified glycans using some characteristic fragment ions, such as core fucose characteristic fragment ions or bisecting GlcNAc characteristic fragment ions (Figure S3). The presence of the core fucose for these N fraction glycans was validated with MS/MS data based on the existence of the core fucose characteristic fragment ions of *m*/*z* = 649.4 (HexNAc2Fuc1-PA(1H+)) or *m*/*z* = 325.0 (HexNAc2Fuc1-PA(2H+)) (Figure S3—N-11, N-12, and N-14).

Overall, 7 out of 11 fucosylated glycans were galactosylated (N-10, 12, 14, 16, 19, 20, and 21) (Table S2), whereas the afucosylated glycans in the neutral fraction (high mannose and asialylated-afucosylated (excluding HM) types) were agalactosylated (N-1 to 6-3), and only 3 were galactosylated (N-8, 15, and 17-1). Of note, seven glycans in the neutral fraction have bisecting GlcNAc glycans (N-10, 15, and 17-1 to 21).

The presence of the bisecting GlcNAc for these glycans was validated with MS/MS data based on the existence of the bisecting GlcNAc characteristic fragment ions of *m*/*z* = 1014.3 (HexNAc1-Hex-HexNAc2Fuc1-PA(1H+)) (Figure S3—N-21).

In the mono-sialylated (A1) fraction, 19 glycans were identified and quantified as the core glycome. The top 3 abundant glycans in the A1 fraction were F(6)A2G(4)2[3]S(6)1, A2G(4)2[3]S(6)1, and F(6)A2G(4)2[6]S(3)1, as each of them had a relative abundance above 10% of the A2G(4)2S(6)2 glycan signal. On the other hand, M5A1[3]G(4)1S(6)1 and M4A1[3]G(4)1S(6)1 were the least abundant core glycans in the A1 fraction. In total, 11 mono-sialylated glycans (A1-8-1, 9, 14-1, 14-2, 15, 17, 19, 20, 21, 24, and 26) were core-fucosylated (sialylated-fucosylated type).

The existence of the core fucose for these A1 glycans was validated with MS/MS data based on the existence of the core fucose characteristic fragment ion of *m*/*z* = 649.4 (HexNAc2Fuc1-PA(1H+)) (Figure S3—A1-9, A-14, A1-15, A1-24).

While the other 8 glycans (A1-2-1, 2-2, 3, 5, 6, and 10 to 12) were afucosylated (sialylated-afucosylated type), most of the glycans contained type 2 LacNAc (Galβ1-4GlcNAc) antennae, a common structure of vertebrate glycans, and some were modified by bisecting GlcNAc (A1-24, 26). The existence of bisecting GlcNAc for these A1 glycans was validated from MS/MS data based on the existence of the bisecting GlcNAc characteristic fragment ions of *m*/*z* = 1014.3 (HexNAc1-Hex-HexNAc2Fuc1-PA(1H+)) (Figure S3—A1-24).

The type 2 LacNAc of these glycans was frequently sialylated by α2,6-linkage rather than α2,3-linkage.

Among the mono-sialylated glycans, sialylation occurred mainly on the α1,3-Man arm. Some glycans (A1-8-1, 14-2, 17) had LacdiNAc (GalNAcβ1-4GlcNAc) antenna.

The existence of LacdiNAc (GalNAcβ1-4GlcNAc) sequences in these A1 glycans was characterized by B ion fragments at *m*/*z* = 407 (HexNAc2) (Figure S3—A1-14-2, A1-17). Moreover, the LacdiNAc-NeuAc1 sequence in the F(6)A2GalNAc2[3]S(6)1 glycan was characterized by a B ion fragment at *m*/*z* = 698.2 (HexNAc2 NeuAc1) (Figure S3—A1-17).

In general, the existence of sialic acid in A1 glycans was elucidated based on a characteristic fragment ion of *m*/*z* = 657.2 ((NeuAc-Hex-HexNAc1) (1H+)) (Figure S3—A1-2-1 to A1-12, A1-15, A1-24).

In the di-sialylated (A2) fraction, nine glycans were identified and quantified. The most abundant glycan in the A2 fraction and all glycomes was A2G(4)2S(6)2, as it represented ~18% of the total glycome signal. F(6)A2G(4)2S(3)2 and F(6)A2G(4)2S(6)2 represented the 2nd and 3rd most abundant glycans, respectively. The afucosylated form from F(6)A2G(4)2S(3)2 was identified as the least abundant core glycan in the A2 fraction. A total of 6 di-sialylated glycans (A2-7-2, 8, and 9 to 12) were fucosylated (sialylated-fucosylated type), and the remaining 3 glycans (A2-2, 4, and 7) were afucosylated (sialylated-afucosylated type). All glycans had type 2 LacNAc antennae, and 2 glycans (A2-7-2 and 9) also had LacdiNAc antenna. The existence of LacdiNAc antenna in these A2 glycans was characterized by a B ion fragment at *m/z* = 698.2 (HexNAc2 NeuAc1) (Figure S3—A2-9).

In general, the presence of di-sialic acids in A2 fraction glycans can be elucidated using the characteristic fragment ions of *m*/*z* = 900.8 ((NeuAc2-Hex5-HexNAc2) (2H+)) or *m*/*z* = 1799.3 ((NeuAc2-Hex5-HexNAc2) (1H+)) (Figure S3—A2-2, A2-8).

In the tri-sialylated (A3) fraction, 5 glycans (A3-2, 4, 13, 14, and 15) were identified and quantified in the core glycome. A total of 4 glycans out of 5 (A3-2 and A3-13 to 15) were fucosylated (sialylated-fucosylated type), while A3G(4)3S3_1 was afucosylated (sialylated-afucosylated type). All of these glycans had a tri-antennary structure, with two branches (Galβ1-4GlcNAc) originating from α1-3 core mannose.

The presence of tri-sialic acids in A3 fraction glycans can be elucidated considering the characteristic fragment ion of *m*/*z* = 1329.8 ((NeuAc4-Hex6-HexNAc3) (2H+)) (Figure S3—A3-4, A3-13).

In the tetra-sialylated (A4) fraction, 3 glycans (A4-5, 7, and 11) were identified and quantified. All of these glycans had core fucose, and 2 of them (A4-5 and A4-7) had a tetra-antennary structure.

The presence of tetra-sialic acids in A4 fraction glycans can be elucidated considering the characteristic fragment ion of *m*/*z* = 1557.0 ((NeuAc4-Hex7-HexNAc4) (2H+)) (Figure S3—A4-5).

### 3.4. Investigating the Glycans Significantly Differentiated between Males and Female in Core Glycome

Given a restricted number of samples to compare male and female groups, we employed bootstrapping as a means of multiple sampling to enhance the statistical tests’ strength and yield more reliable outcomes in comparing the two genders.

We generated a thousand random samples by resampling our initial core glycome dataset, each with a replacement. We then computed the difference between the mean values of the male and female groups for each glycan structure in each bootstrapped sample and used it to create a distribution. This distribution was used to assess the significant differences between the two groups by computing the *p*-value.

The bootstrapped data revealed that out of 58 glycans, 5 exhibited considerable variation between the male and female groups. Of the 5, 4 (A2BG(4)2, A2G(4)2S(3,6)2, A2G(4)2S(3)2, and F(6)A2F(3)G3S1) had significantly higher levels in females compared to males, while F(6)A3G(4)3S4_2 had notably higher levels in males than in females.

**Figure 5:**
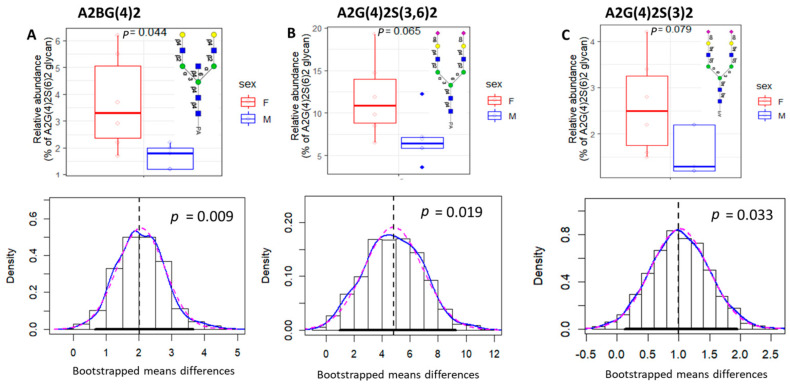

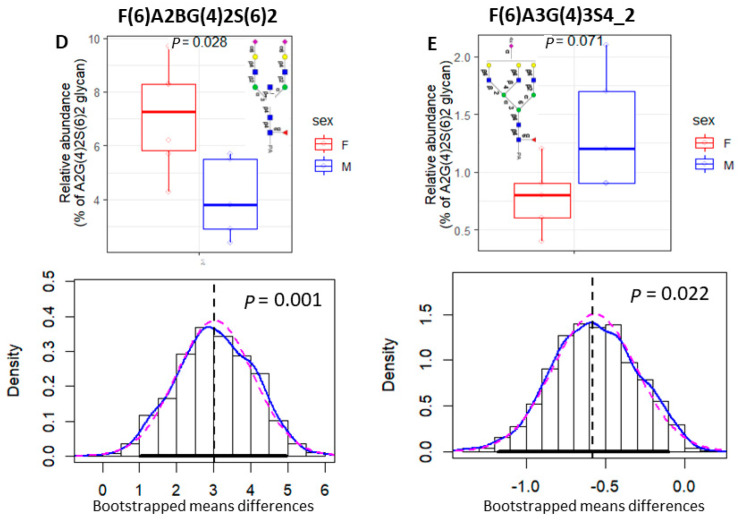
Each glycan was represented by an upper boxplot showing the distribution of the original data combined with the calculated *p*-value and a bottom density plot showing the distribution of the mean differences of the bootstrapped samples combined with the calculated *p*-value. The dashed vertical line represents the mean difference in the original data, the bold horizontal line represents the 95% confidence interval (CI), the dotted red line represents normal density, and the blue one represents kernel density. (**A**–**D**) show the significantly elevated glycans in females (N = 6) compared to males (N = 6) (*p*-value < 0.05) in bootstrapped samples. (E) shows the significantly elevated glycan structures in males compared to females (*p*-value < 0.05) in bootstrapped samples.

It is worth noting that the bootstrapping technique was effective in confirming the significant difference shown by the Wilcoxon test (*p*-value < 0.05) for 2 glycans (A2BG(4)2, F(6)A2BG(4)2S(6)2), as demonstrated in Figure 5A,D. Moreover, the technique detected a significant difference for three glycans (A2G(4)2S(3,6)2, A2G(4)2S(3)2, and F(6)A3G(4)3S4_2) that were not identified as significant in the original samples by the Wilcoxon test, which is also known as false negatives. (Figure 5B,C,E). Notably, the distribution of bootstrapped means differences (Figure 5—bottom) showed that a 95% confidence interval (CI) (denoted by a bold horizontal line) did not contain 0 for all significantly differentiated glycans.

Concerning the characteristics of these glycans, four out of the five distinct glycans contained sialic acid. Among these four glycans, two were categorized as fucosylated (F(6)A2BG(4)2S(6)2), and F(6)A3G(4)3S4_2) and two (A2G(4)2S(3,6)2 and A2G(4)2S(3)2)) were classified as A-fucosylated.

The female glycans exhibited relatively greater variation than those in males. This variation in females could potentially be due to fluctuations in the secretion of ovarian hormones.

It should be noted that the differences in the glycans between males and females may be related to their binding to gender-specific proteins or sex hormones. For instance, A2BG(4)2 (H5Hn5PA1) may be attached to proteins that are highly expressed in females, such as immunoglobulins [35], or female enriched proteins, such as Choriogonadotropin alpha and beta chains [36], which could explain why these glycans are more abundant in females than in males.

On the other hand, a further investigation of the F(6)A3G(4)3S4_2 glycan in the GlyConnect database (https://glyconnect.expasy.org/browser/compositions/1238 (accessed on 8 December 2022)) revealed that they only attach to either Erythropoietin or Alpha-1-antitrypsin proteins. These proteins may positively contribute to the abundance of the F(6)A3G(4)3S4_2 glycan (N4H6Hn5dH1PA1) in males compared to females.

### 3.5. Investigating the Glycans Significantly Correlated with Age in the Core Glycome

**Figure 6:**
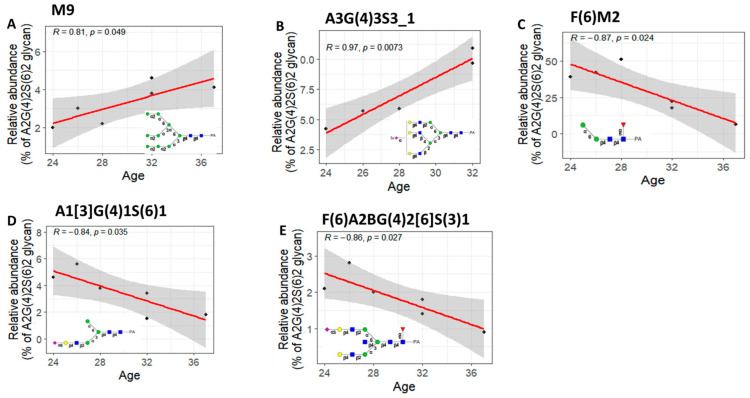
(**A**,**B**) Correlation plot showing a significant positive correlation (R > 0.8 & *p* < 0.05) between relative abundances of the M9 and A3G(4)3S3_1 glycans and age in females (N = 6). (**C**–**E**) Correlation plot showing a significant negative correlation (R < −0.8 & *p* < 0.05) between relative abundances of the F(6)M2, A1[3]G(4)1S(6)1, and F(6)A2BG(4)2[6]S(3)1 glycans and age in female.

It is widely recognized that men and women utilize physiological pathways to achieve longevity, with women generally living longer than men. Therefore, we studied the connection between gender and glycan values at various ages. Our findings revealed that in females, the relative abundances of three glycans (F(6)M2, A1[3]G(4)1S(6)1, and F(6)A2BG(4)2[6]S(3)1) were negatively correlated with age (Figure 6C–E), whereas the relative abundances of two glycans (M9 and A3G(4)3S3_1) showed a positive correlation with age (Figure 6A,B).

**Figure 7:**
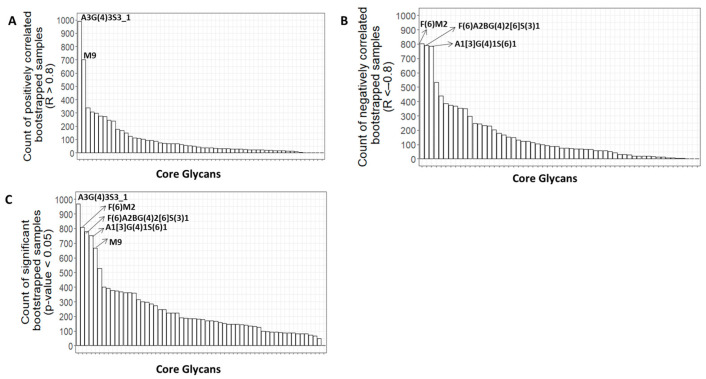
(**A**) Bar plot displaying the number of positively correlated bootstrapped samples (R > 0.8) between each core glycan and age in females (N = 6). (**B**). Bar plot displays the number of negatively correlated bootstrapped samples (R < −0.8) between each core glycan and age in females. (**C**) Bar plot displaying the number of significantly correlated bootstrapped samples (*p*-value < 0.05) between each core glycan and age in females.

In males, the relative abundance of F(6)A1[3]G(4)1S1 showed a negative correlation with age (Figure 7). All negative correlations have a Pearson correlation coefficient (R < −0.8) and *p*-value < 0.05. Additionally, all positive correlations have a Pearson correlation coefficient (R > 0.8) and *p*-value < 0.05. Our results showed that there is an interaction between age and sex in the urine *N*-glycosylation features, with more dynamic changes in females than males.

**Figure 8:**
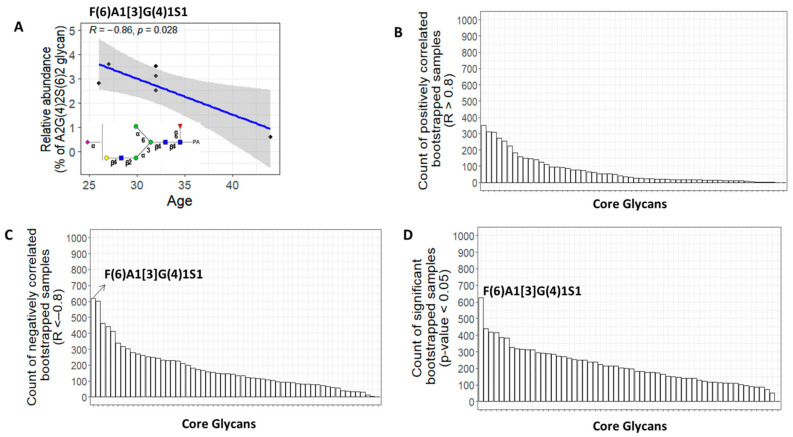
(**A**) Correlation plot showing a significant negative correlation (R < −0.8 & *p* < 0.05) between the relative abundance of the F(6)A1[3]G(4)1S1 glycan and age in males (N = 6). (**B**) Bar plot displaying the number of positively correlated bootstrapped samples (R > 0.8) between each core glycan and age in males. (**C**) Bar plot displaying the number of negatively correlated bootstrapped samples (R < −0.8) between each core glycan and age in males. (**D**) Bar plot displaying the number of significantly correlated bootstrapped samples (*p*-value < 0.05) between each core glycan and age in males.

To confirm the glycans that were significantly correlated with age and to identify false negatives that were missed in the original data, the bootstrapping technique was used. The process involved creating a thousand random samples by resampling the initial core glycome dataset, each with replacement. The R and *p*-values were then calculated for each core glycan in each bootstrapped sample for a specific gender. The frequency of positively correlated bootstrapped samples (R > 0.8), negatively correlated bootstrapped samples (R < −0.8), and significantly correlated bootstrapped samples (*p*-value < 0.05) were determined for each glycan in both the male and female groups. The results, as shown in Figure 7 and Figure 8, indicated that the most frequent glycans in the bootstrapping analysis were the ones that displayed a significant correlation with age in the original data for males or females.

The glycans’ association with age could potentially be explained by the age-related associations of their precursor proteins. Research examining the plasma proteomic profiles of adults revealed a group of proteins and pathways that were significantly linked to chronological age and were better indicators of biological age [37]. For instance, the study found that proteins such as FSH, LH, and SHGB were positively correlated with age only in women, while netrin-4 was significantly negatively associated with age only in men.

Previous research on the urinary glycome has highlighted the differentiated urinary glycans in different genders and ages. Li et al. (2020) conducted a comprehensive MS analysis of *N*-glycans present in urine samples from both adult and pediatric populations. The *N*-glycans released by PNGase F were isolated, labeled with aminobenzoic acid, and methylamidated before being analyzed using LC-MS/MS. The authors were able to detect 116 *N*-glycan compositions, out of which 46 were reproducibly detected and quantified. We checked the existence of these 46 glycans in our core glycome, which consists of 41 glycan compositions corresponding to 58 core glycan structures. We found that >70% of the glycan compositions are common between both studies; however, our study provides more information at the glycan structural level. For instance, there is agreement between both studies that the most abundant glycan in urine is the N2H5Hn4 glycan composition, as it represents ~21% of the total glycome signal. Yet, we indicated that the A2G(4)2S(6)2 glycan structure is the most abundant among others that have the same composition, as it represents ~18% of the total glycome signal, and other glycan structures (A2G(4)2S(3,6)2 and A2G(4)2S(3)2) represent the minority, where A2G(4)2S(3,6)2 represents ~10% of the abundance of the A2G(4)2S(6)2 glycan and A2G(4)2S(3)2 represents ~2% of the A2G(4)2S(3)2 glycan signal.

Additionally, they found that 42 glycan compositions out of 46 showed a significant difference between different ages and sex groups, but our study found only a small number of glycans that differed significantly between genders and ages at the glycan structure level. Another recent study also suggested that male and female urine *N*-glycan profiles have few differences [38]. The discrepancies observed in these studies could be attributed to different factors, such as the sample size, methodology, and data analysis. For instance, Li et al. (2020) utilized PNGase F for glycan isolation, while we used hydrazinolysis. Additionally, they used aminobenzoic acid for labeling, while we used PA. Moreover, we used the Wilcoxon test to detect the differences in the glycan relative abundances between males and females, since glycan relative abundances are not normally distributed. However, Li et al. (2020) used the t-test to detect the differences of glycan MS1 peak areas between males and females.

We also acknowledge that there is great variety in the living environments and eating habits between the cohorts of our study and others, which may account for individual differences and lead to new findings. It is worth noting that the gender and age differences in this study could be affected by the limited sample size.

In light of the biomarker discovery, different studies indicate different candidate biomarkers for different human diseases in terms of the glycan type or glycan composition. For instance, Ganglong Yang et al. [39] revealed that terminal GalNAc and Gal, core-fucosylated *N*-glycans and high mannose-type *N*-glycans, were more highly expressed in bladder cancer cells and tissues than in normal cells. However, identifying glycan biomarkers at the structural level can provide higher sensitivity and specificity. Additionally, structural information can differentiate between different glycan isomers, which is important in the development of targeted therapies. Thus, the quantification data of the core glycome presented in this study can serve as a potential reference for future studies interested in discovering glycan biomarkers and developing targeted therapies for various human diseases at the glycan structure level.

## 4. Conclusions

Urine biofluid outperformed other biofluids, such as blood, as it can be collected non-invasively in large volumes with high stability and less complexity. Moreover, the urinary glycome can reflect both the systemic and urogenital physiology, as it is linked to proteins that come from systemic circulation, as well as those released from the kidney and the urogenital tract. Thus, the human glycome profile was investigated in urine under normal physiological conditions using LC-MS/MS. Urinary glycans were fractionated by DEAE HPLC and reversed-phase (RP)-LC before MS-MS/MS analysis to reduce the sample complexity. Mainly mass spectrometry data were used to identify 67 distinct compositions of the urinary *N*-glycan. The retention time of PA-glycans from the C18 stationary phase was used to elucidate the structural features of these PA-glycans and to distinguish among different glycan isomers, where the addition of certain monosaccharides to PA-glycan contributed either positively or negatively to the RP retention time. Consequently, 109 distinct glycan structures were identified. Of them, 58 distinct glycan structures showed a tendency to be identified in at least 80% of samples. Additionally, some characteristic fragment ions from the MS/MS data were used to elucidate the structural features of the *N*-glycans, such as the characteristic fragment ions that validate the existence of core fucose or bisecting GlcNAc in the identified *N*-glycan. The comparative glycome profile between urine and serum showed that approximately 50% of the urinary *N*-glycome is uniquely detected in urine, shedding light on some perspective candidate biomarkers for diseases related to the kidney and urinary tract. On the other hand, the other 50% of urinary *N*-glycans are shared with serum; this can be explained by the incomplete reabsorption of these *N*-glycans in tubules. Additionally, gender and age differences were also observed in the urinary *N*-glycan profile. This urinary *N*-glycan profiling can aid future glycan studies in the discovery of novel biomarkers for diagnosing different diseases in the context of gender and age differences.

## Data Availability

Data is represented within this article and Supplementary materials.

## Supplementary Materials

The following supporting information can be downloaded at: https://www.mdpi.com/article/10.3390/biom13050756/s1, Figure S1: DEAE HPLC of human urine *N*-glycans; Figure S2: Chromatograms of reversed-phase HPLC for each sample; Figure S3: MS and MS/MS spectra for some of the glycans detected in at least 10 samples (the core urinary glycome); Table S1: Human urine sample information; Table S2: Characterization list for 109 urinary *N*-glycans detected in urine samples from 12 healthy donors; Table S3: the list of 24 glycans detected exclusively in serum.
