# Supplementary material for: Microheterogeneity and Individual Differences of Human Urinary N-Glycome under Normal Physiological Conditions
Source: Biomolecules. 2023 Apr 27;13(5):756. doi: 10.3390/biom13050756 (PMC10216293; doi:10.3390/biom13050756)
Supplement: Supplementary file 1 [file biomolecules-13-00756-s001.zip › suplementry data/Figure S3.pdf]

Supplementary_data3:

MS and MS/MS spectra for some of the glycans detected in at least 10 samples (the core urinary glycome) in each fraction(N, A1, A2, A3, A4).

Figures N-1 : N-21. glycans in Neutral fraction (N).

Figures A1-2 : A1-24. glycans in Mono-sailylated fraction(A1).

Figures A2-2 : A2-12. glycans in Di-sailylated fraction (A2).

Figures A3-4 , A3-13. glycans in Tri-sailylated fraction (A3).

Figures A4-5. glycans in Tetra-sailylated fraction (A4).

GlycoWorkbench software was used to draw the glycan structure and to assign the fragment ions to their precursors.

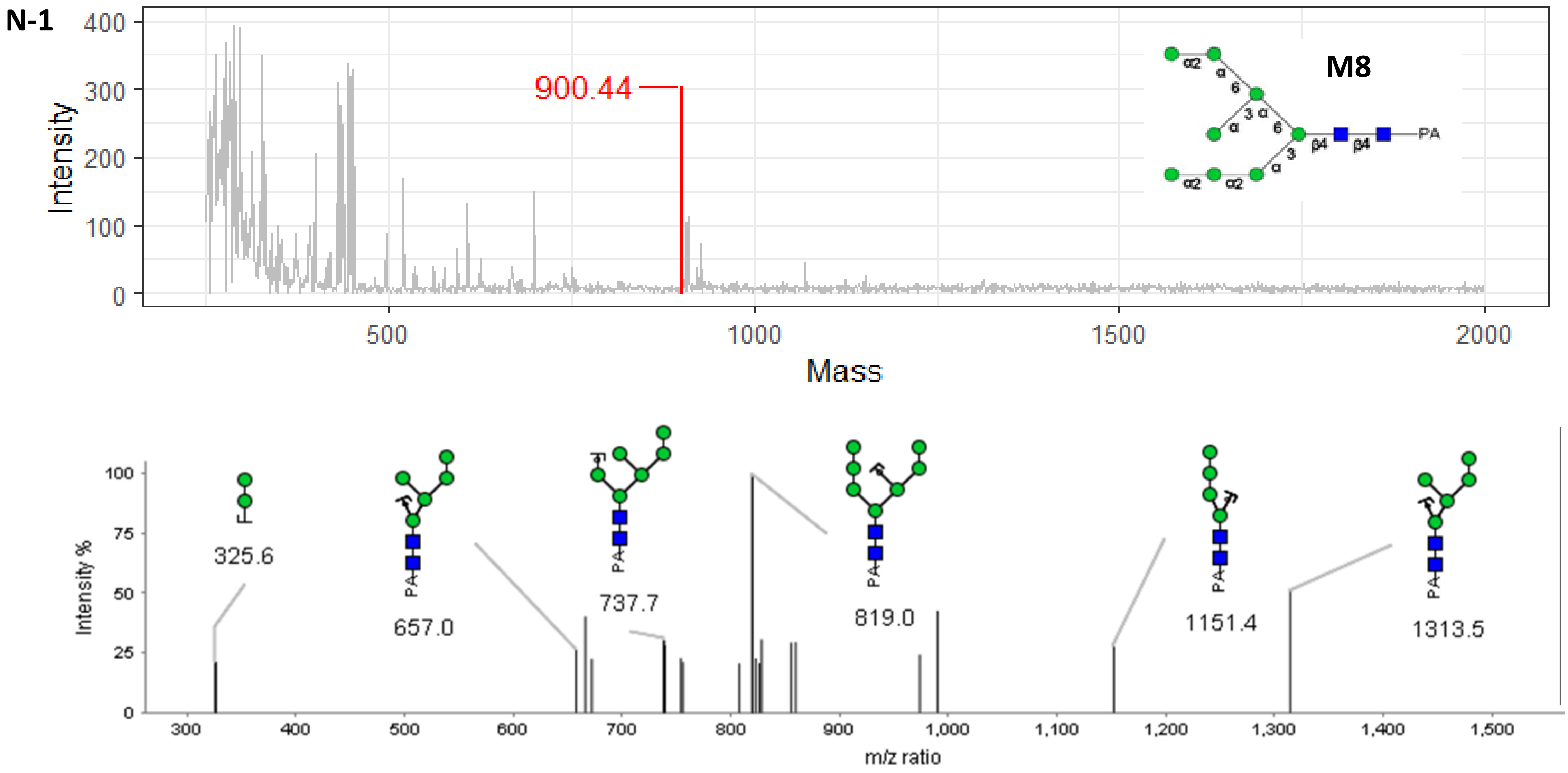
N-1
M8
900.44
Intensity
Mass
α2
α
6
3
β4
PA
325.6
657.0
737.7
819.0
1151.4
1313.5
Intensity %
m/z ratio

N-2

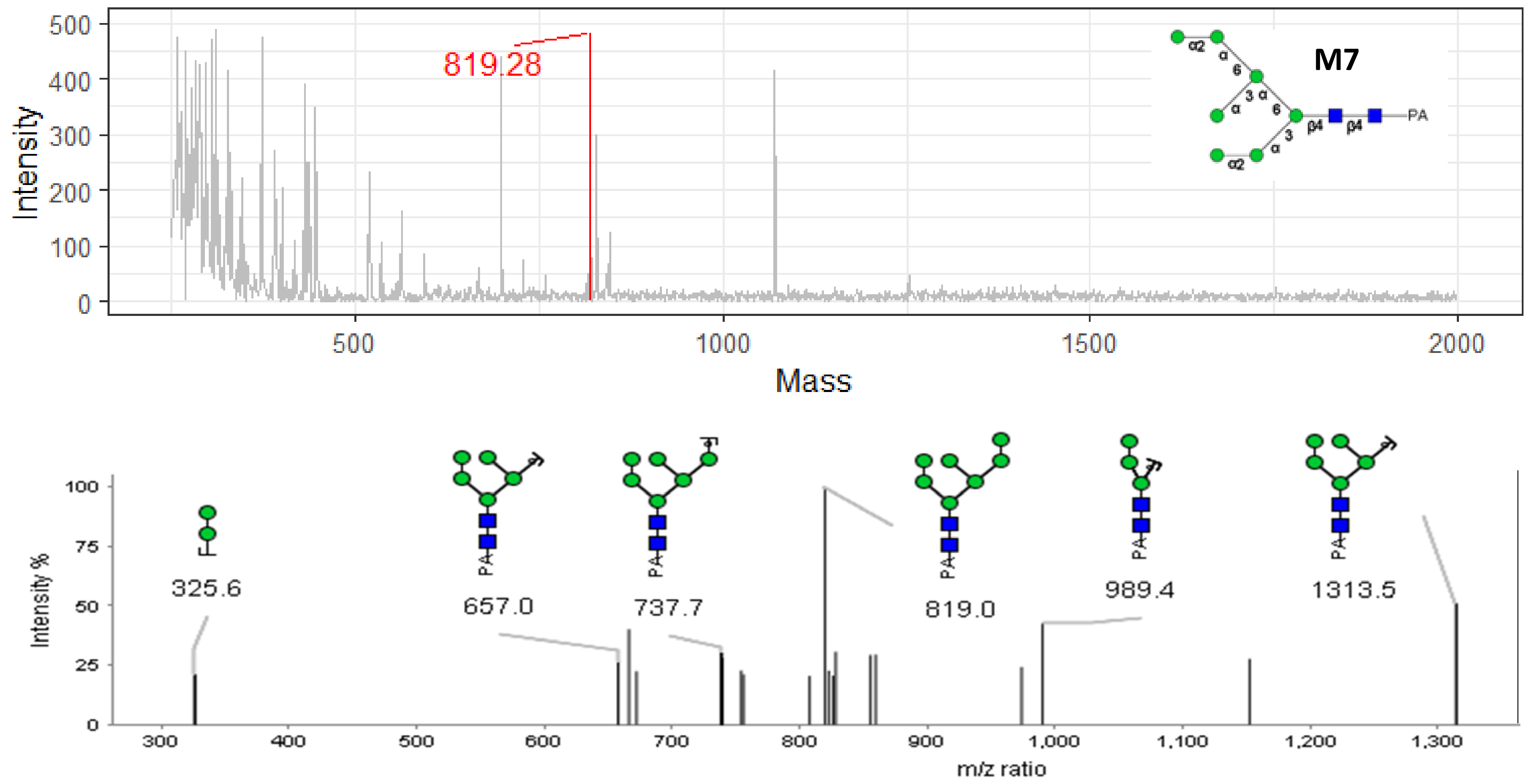

819.28
M7
α2
α
6
3 α
6
α
3
β4
β4
PA
α
α2
Intensity
Mass
500
1000
1500
2000
Intensity %
325.6
657.0
737.7
819.0
989.4
1313.5
m/z ratio

N-3

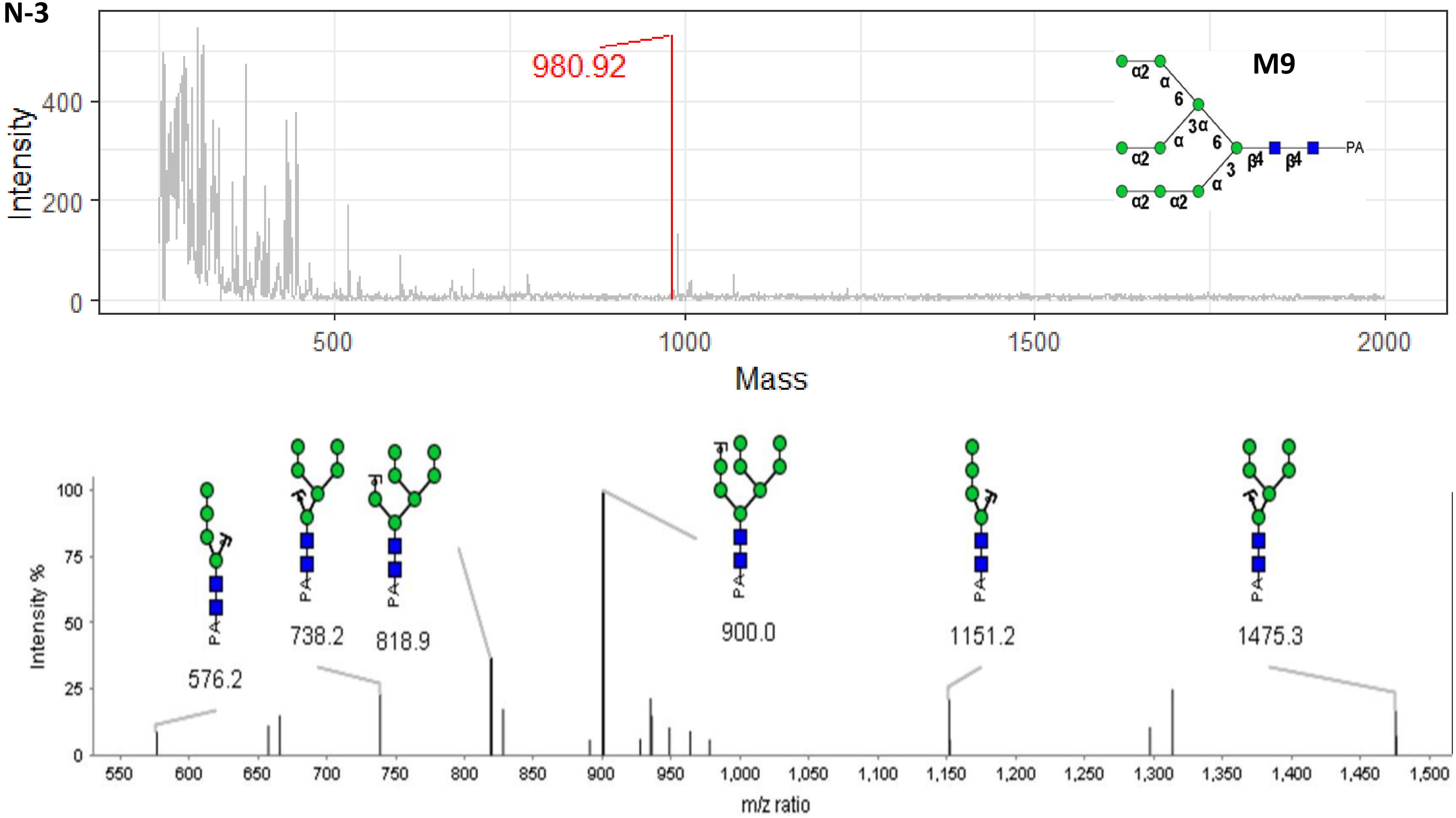

980.92
M9
α2
α
6
3α
α2
α2
3
β4
PA
Intensity
Mass
400
200
0
500
1000
1500
2000
Intensity %
m/z ratio
576.2
738.2
818.9
900.0
1151.2
1475.3

N-5

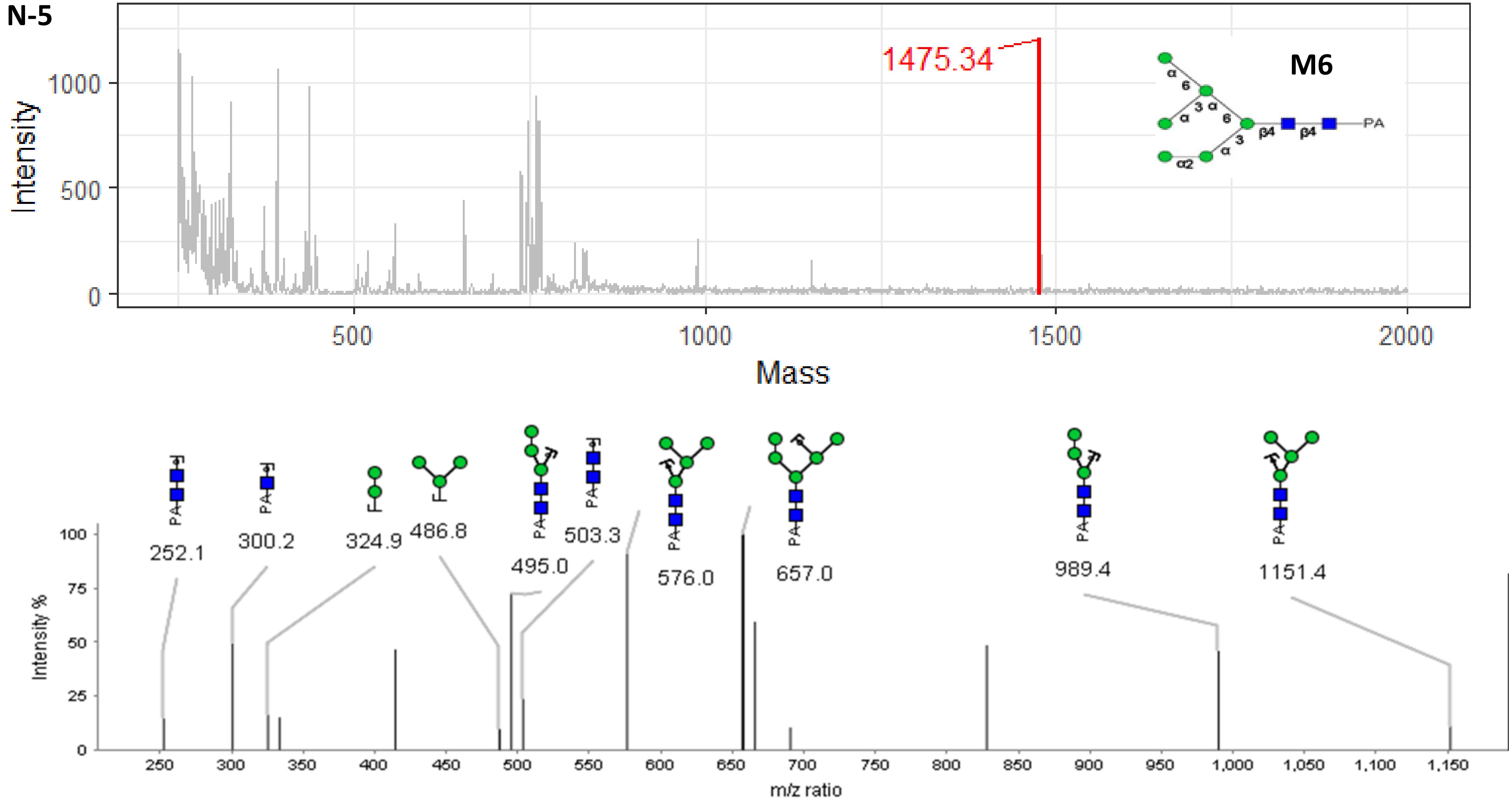

1475.34
M6
Intensity
Mass
PA
252.1
300.2
324.9
486.8
495.0
503.3
576.0
657.0
989.4
1151.4
Intensity %
m/z ratio

N-6-1

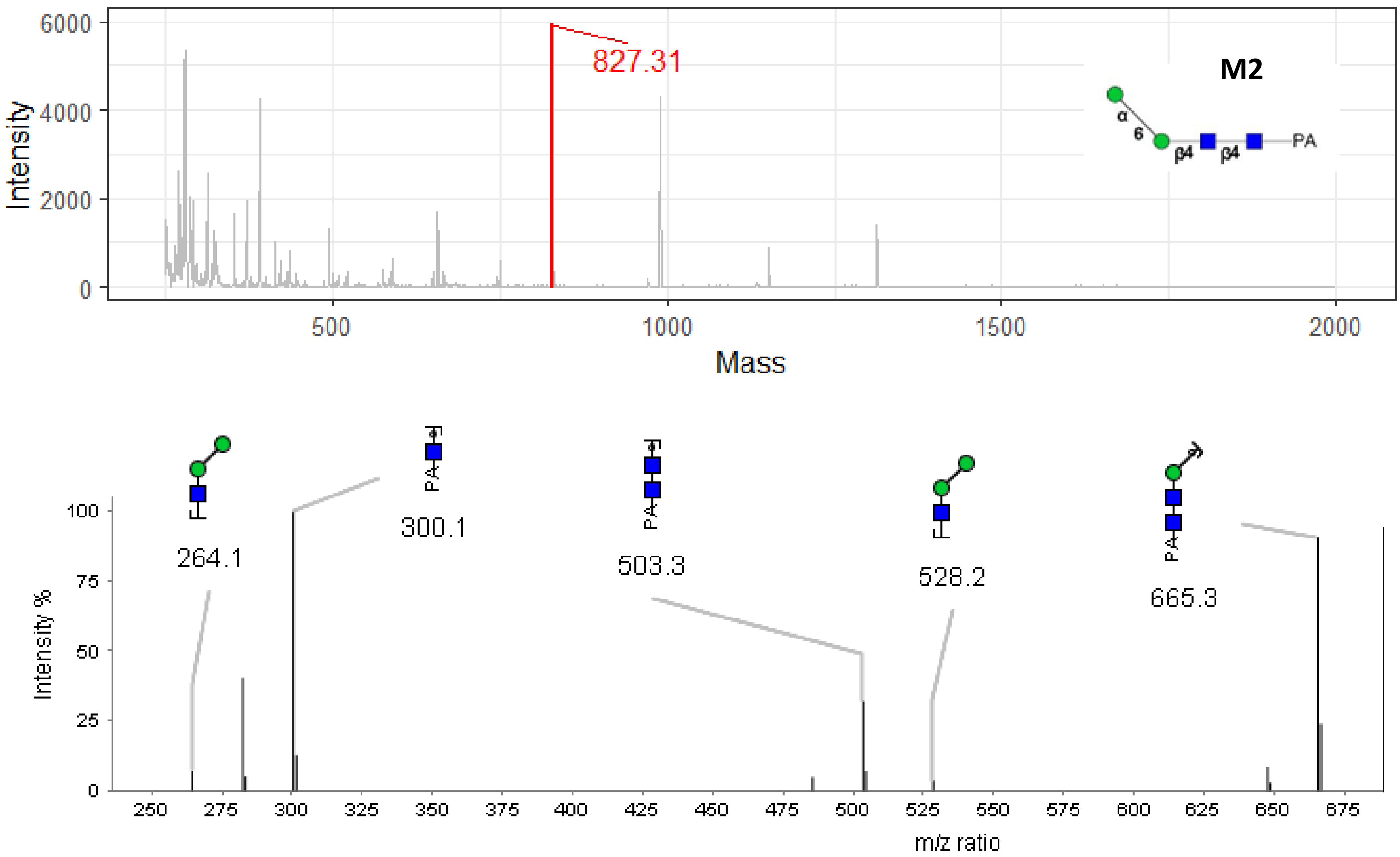

6000
4000
2000
0
Intensity
827.31
M2
α
6
β4
β4
PA
500
1000
1500
2000
Mass
PA
300.1
PA
503.3
264.1
528.2
PA
665.3
100
75
50
25
0
Intensity %
250
275
300
325
350
375
400
425
450
475
500
525
550
575
600
625
650
675
m/z ratio

N-6-2

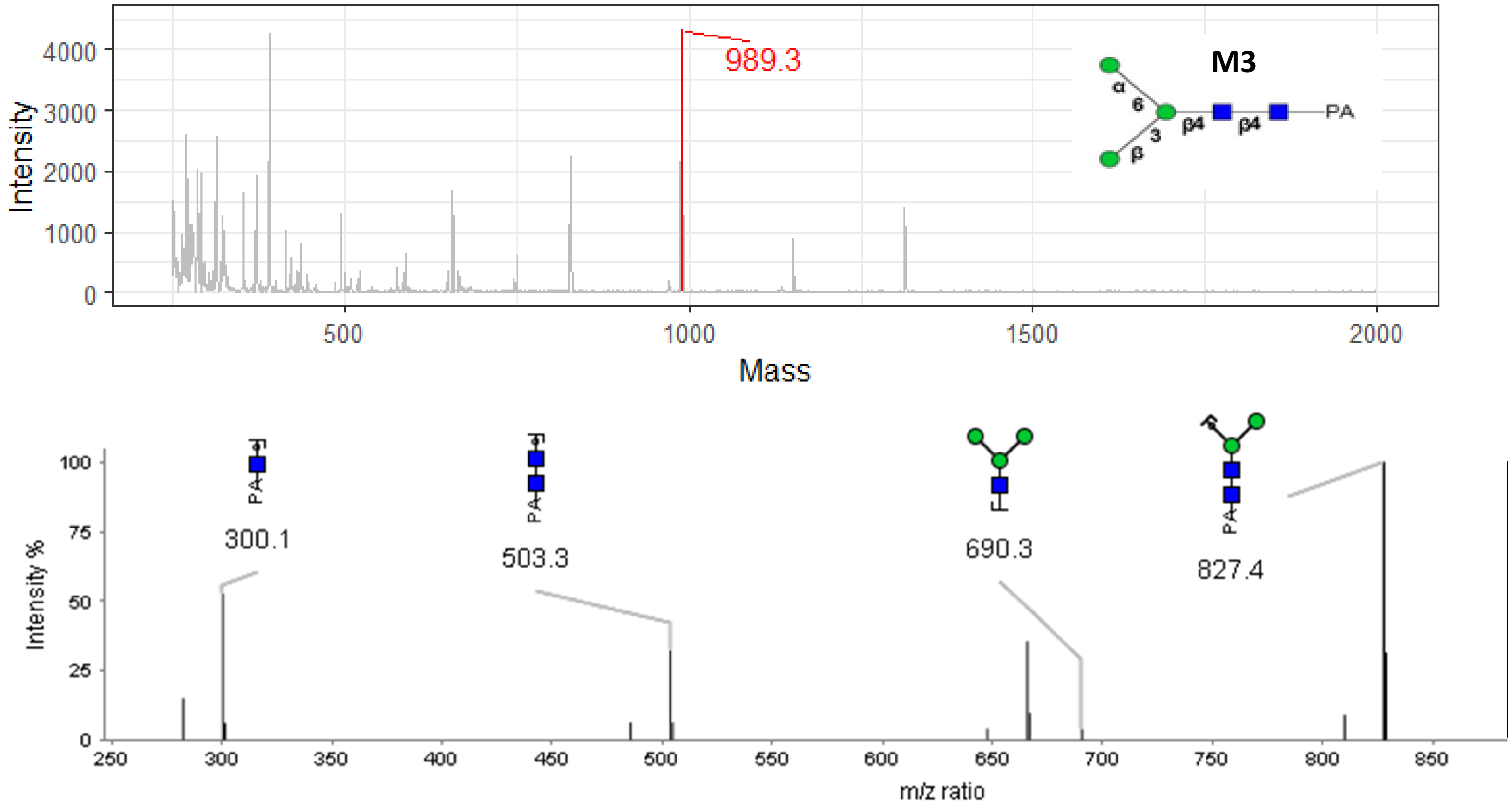
989.3
M3
α
6
3
β
β4
β4
PA
Intensity
4000
3000
2000
1000
0
500
1000
1500
2000
Mass
300.1
503.3
690.3
827.4
Intensity %
100
75
50
25
0
250
300
350
400
450
500
550
600
650
700
750
800
850
m/z ratio

N-6-3

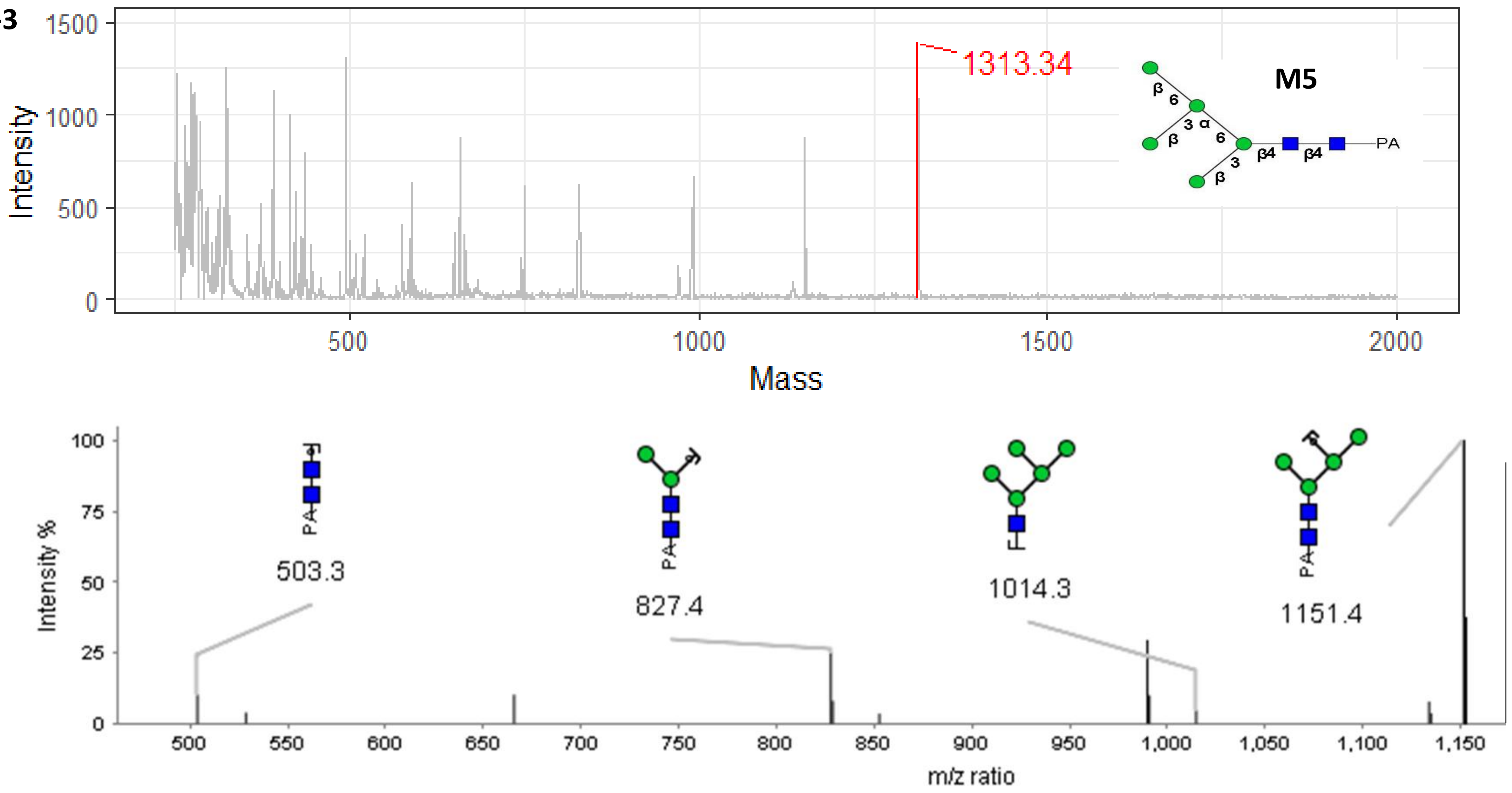

1500
1000
500
0
Intensity
1313.34
M5
β
6
3
α
β
6
3
β
β4
β4
PA
500
1000
1500
2000
Mass
100
75
50
25
0
Intensity %
PA
503.3
827.4
1014.3
1151.4
500
550
600
650
700
750
800
850
900
950
1,000
1,050
1,100
1,150
m/z ratio

N-9-1

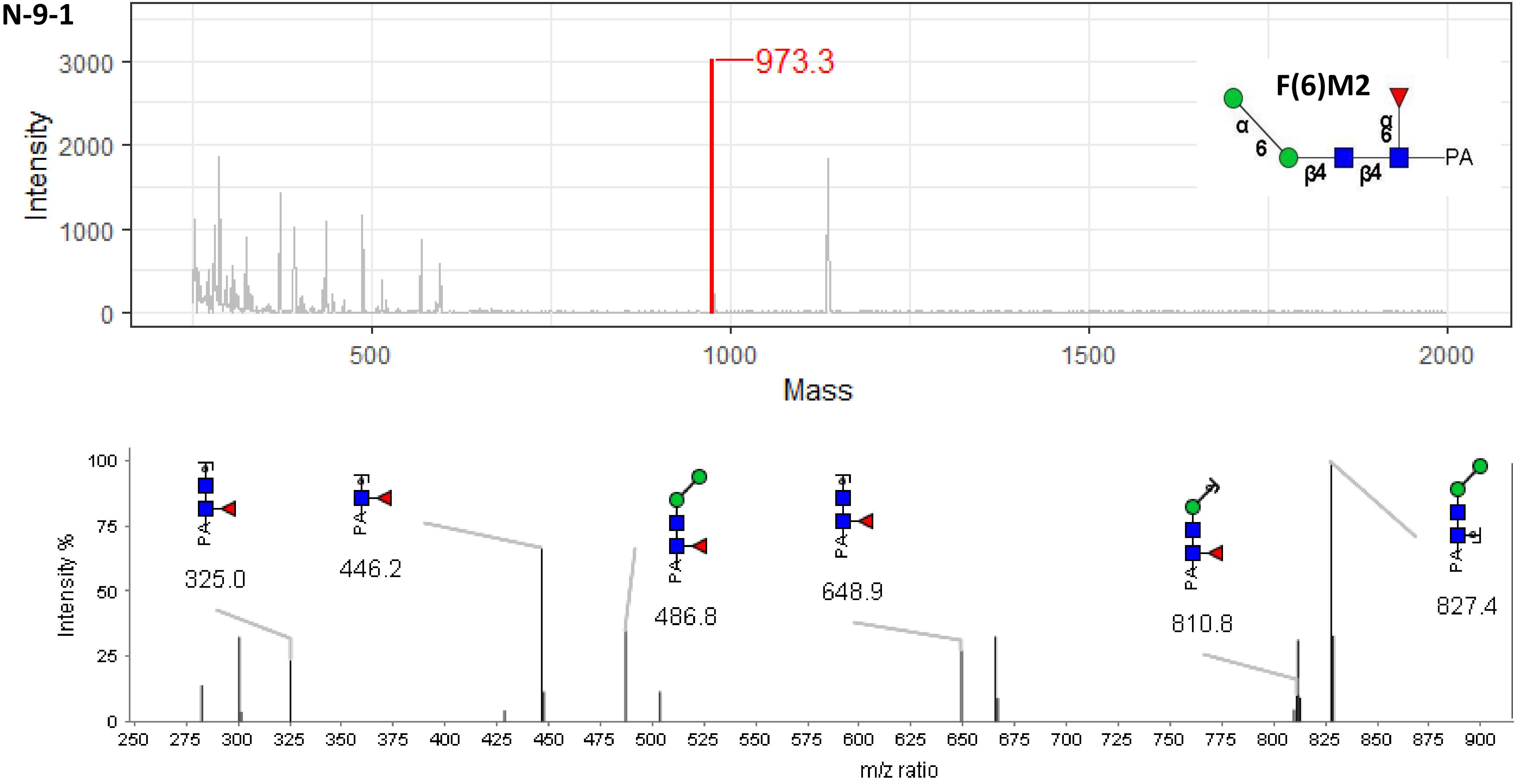
973.3
F(6)M2
α
6
β4
β4
α
6
PA
Intensity
3000
2000
1000
0
500
1000
1500
2000
Mass
Intensity %
100
75
50
25
0
325.0
446.2
486.8
648.9
810.8
827.4
250
275
300
325
350
375
400
425
450
475
500
525
550
575
600
625
650
675
700
725
750
775
800
825
850
875
900
m/z ratio

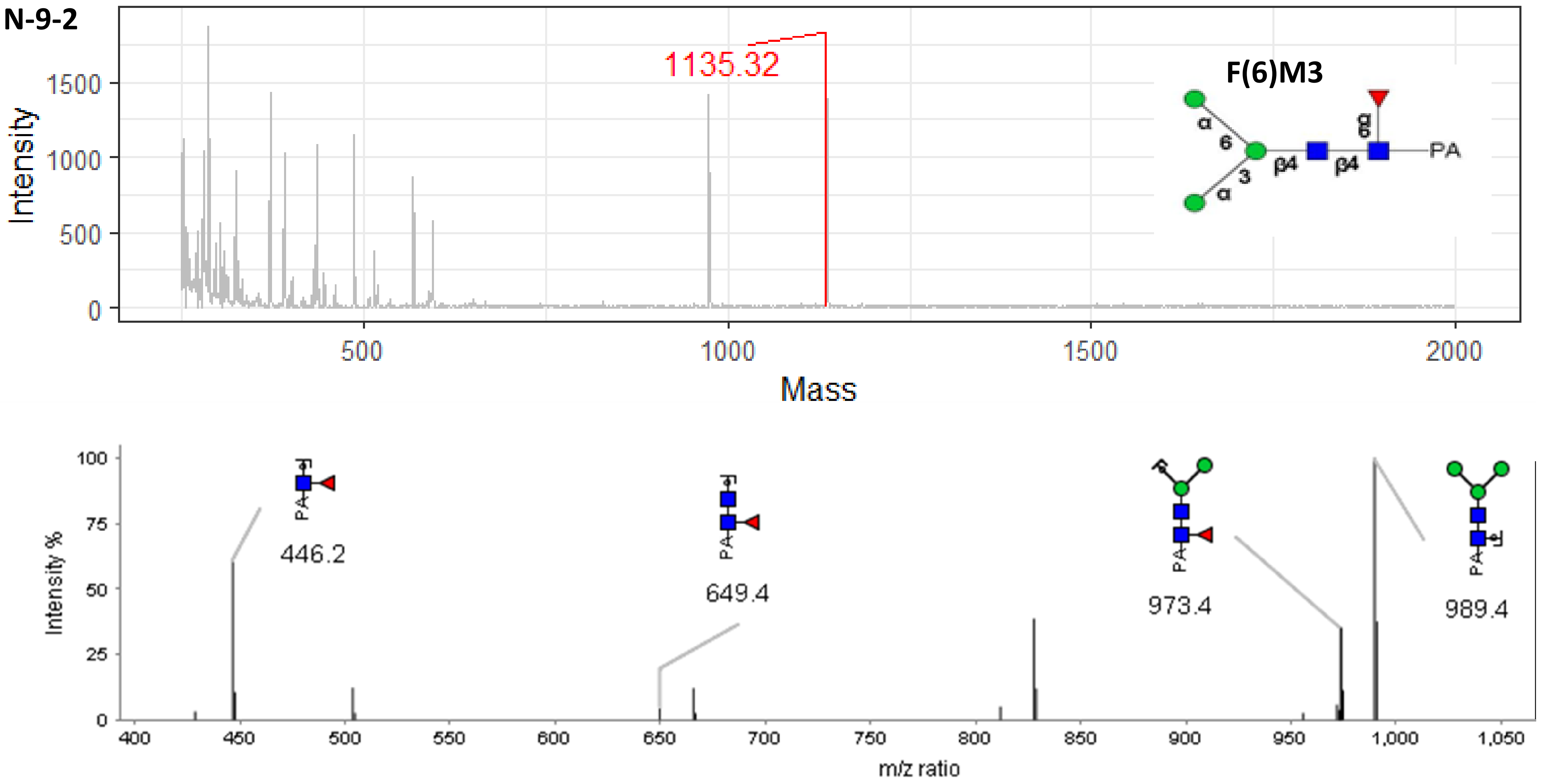

N-9-2
1135.32
F(6)M3
PA
Intensity
Mass
0
500
1000
1500
2000
446.2
649.4
973.4
989.4
Intensity %
m/z ratio
400
450
500
550
600
650
700
750
800
850
900
950
1,000
1,050

N-11

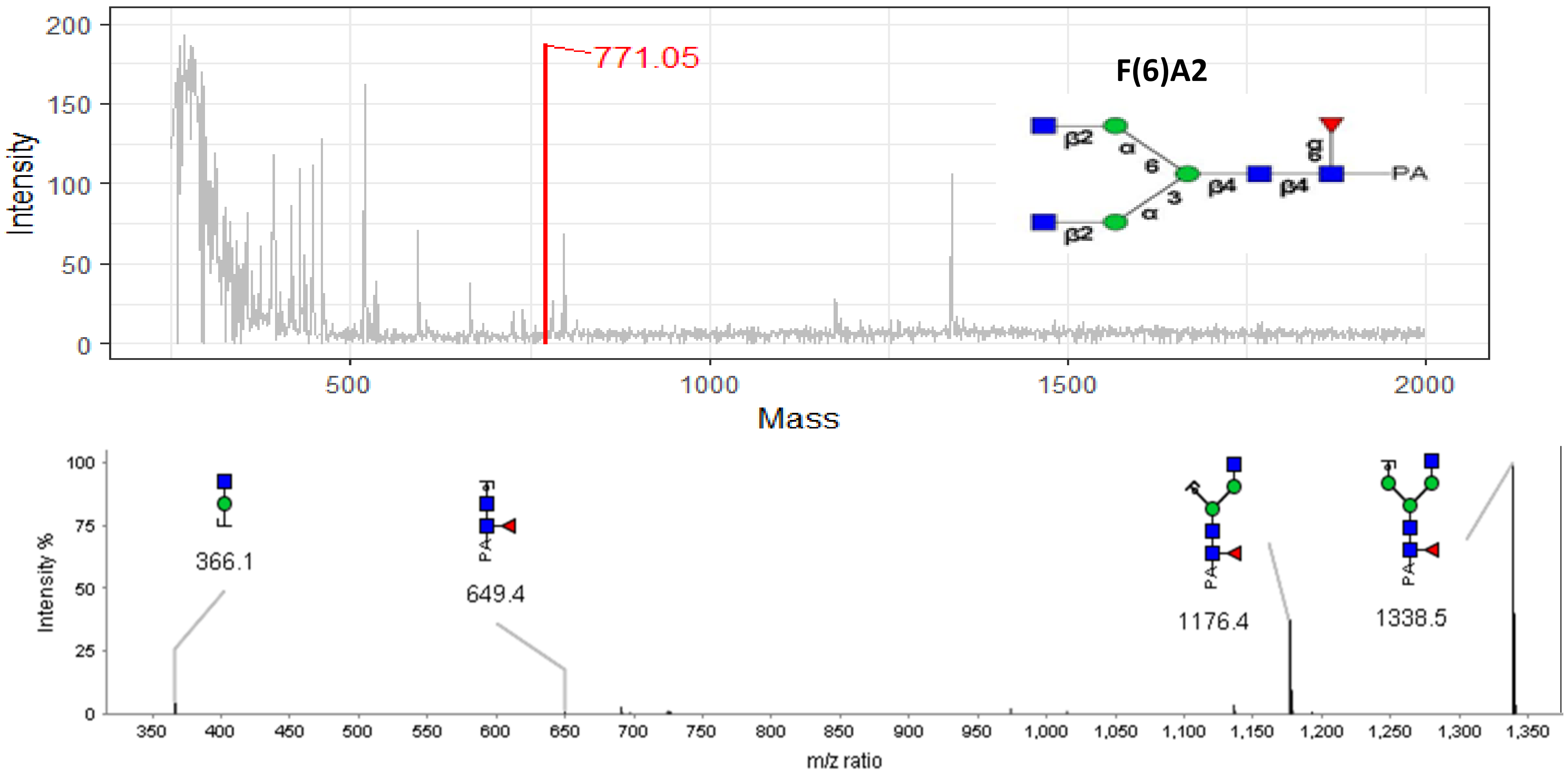

Intensity
Mass
771.05
F(6)A2
PA
366.1
649.4
1176.4
1338.5
Intensity %
m/z ratio

N-12

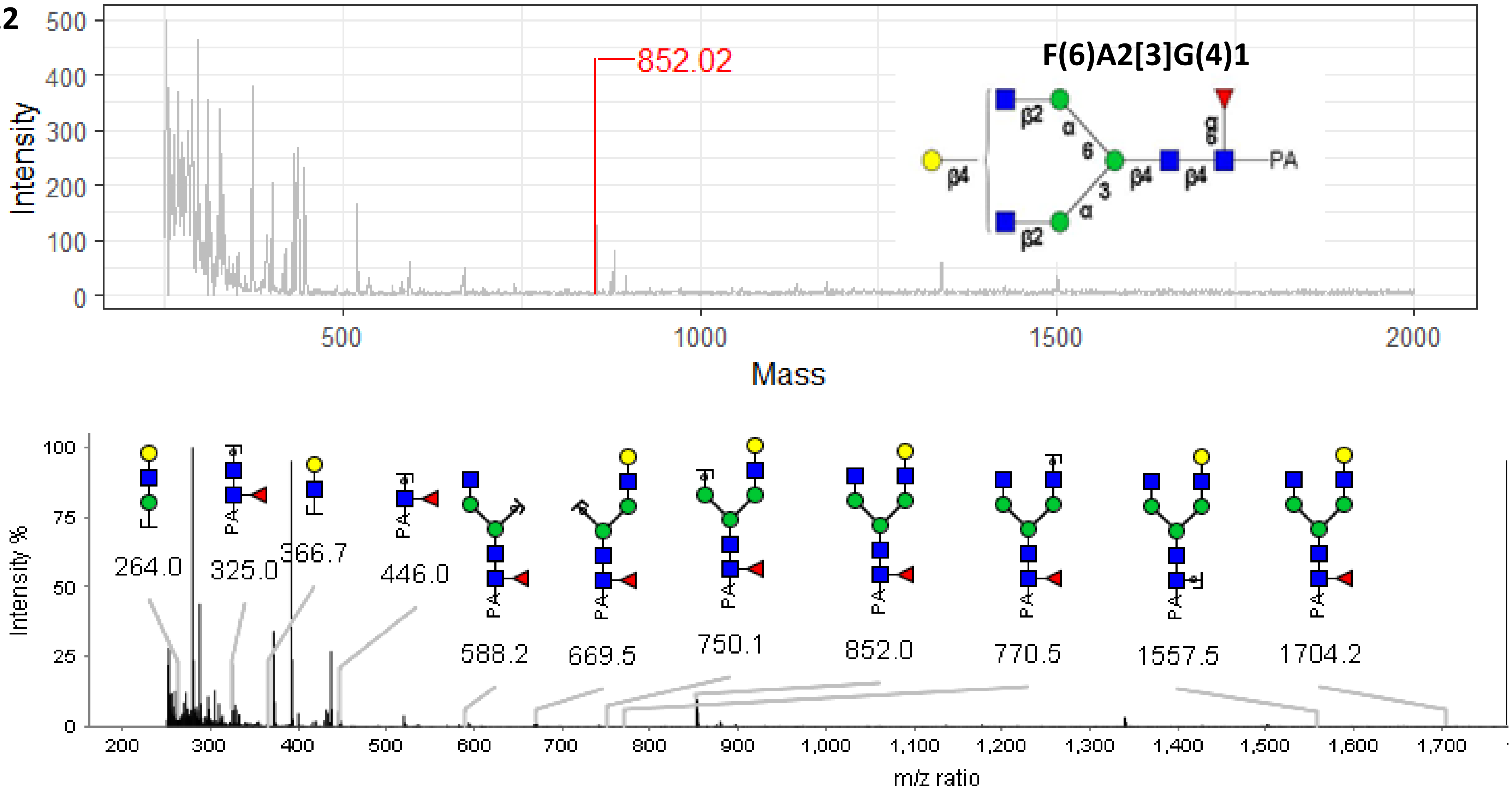

F(6)A2[3]G(4)1
852.02
Intensity
Mass
PA
264.0
325.0
366.7
446.0
588.2
669.5
750.1
852.0
770.5
1557.5
1704.2
Intensity %
m/z ratio

N-14

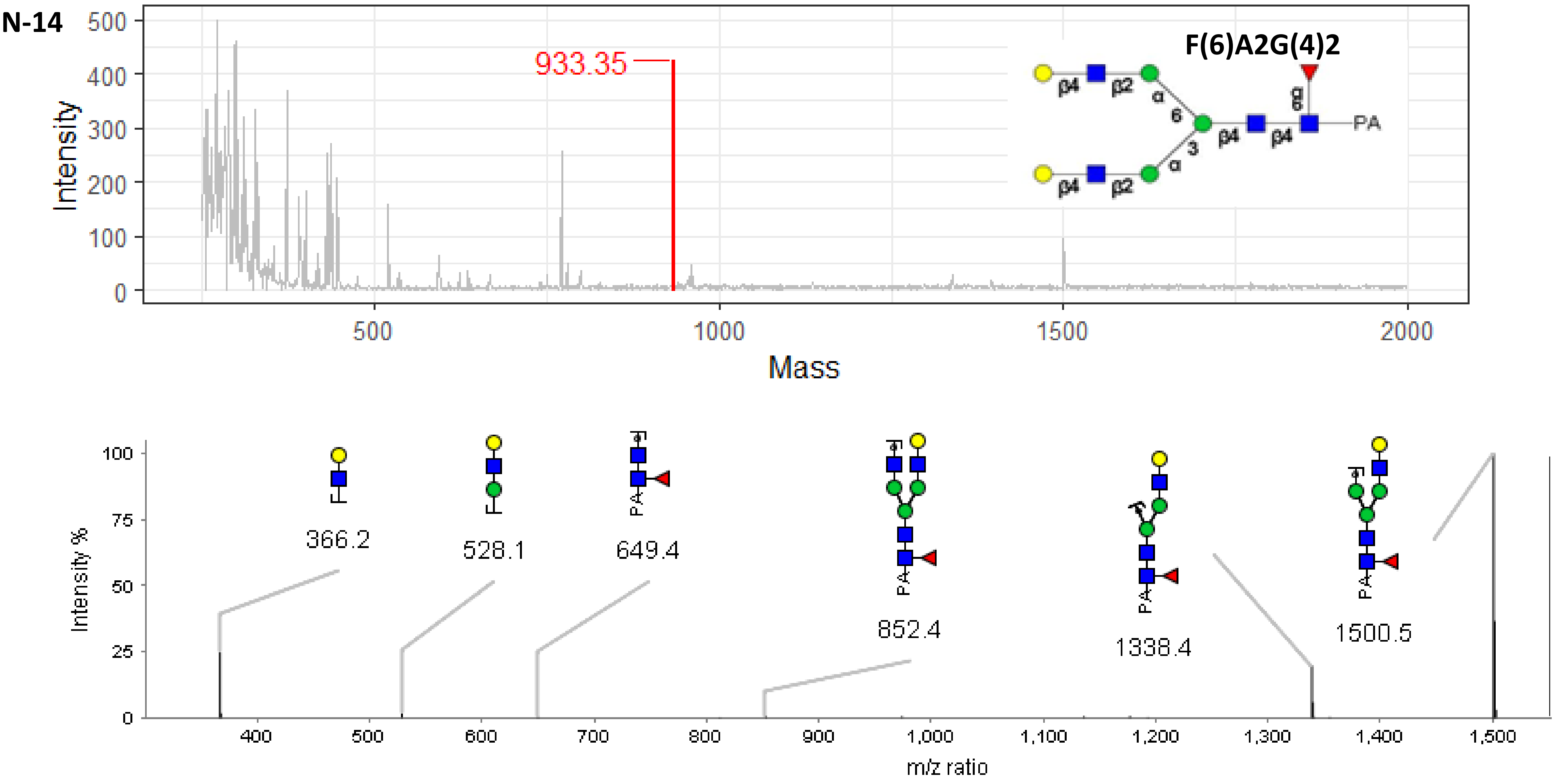
F(6)A2G(4)2
933.35
Intensity
Mass
500
400
300
200
100
0
500
1000
1500
2000
β4
β2
α
6
3
PA
366.2
528.1
649.4
852.4
1338.4
1500.5
Intensity %
m/z ratio
100
75
50
25
400
600
700
800
900
1,000
1,100
1,200
1,300
1,400
1,500

N-21

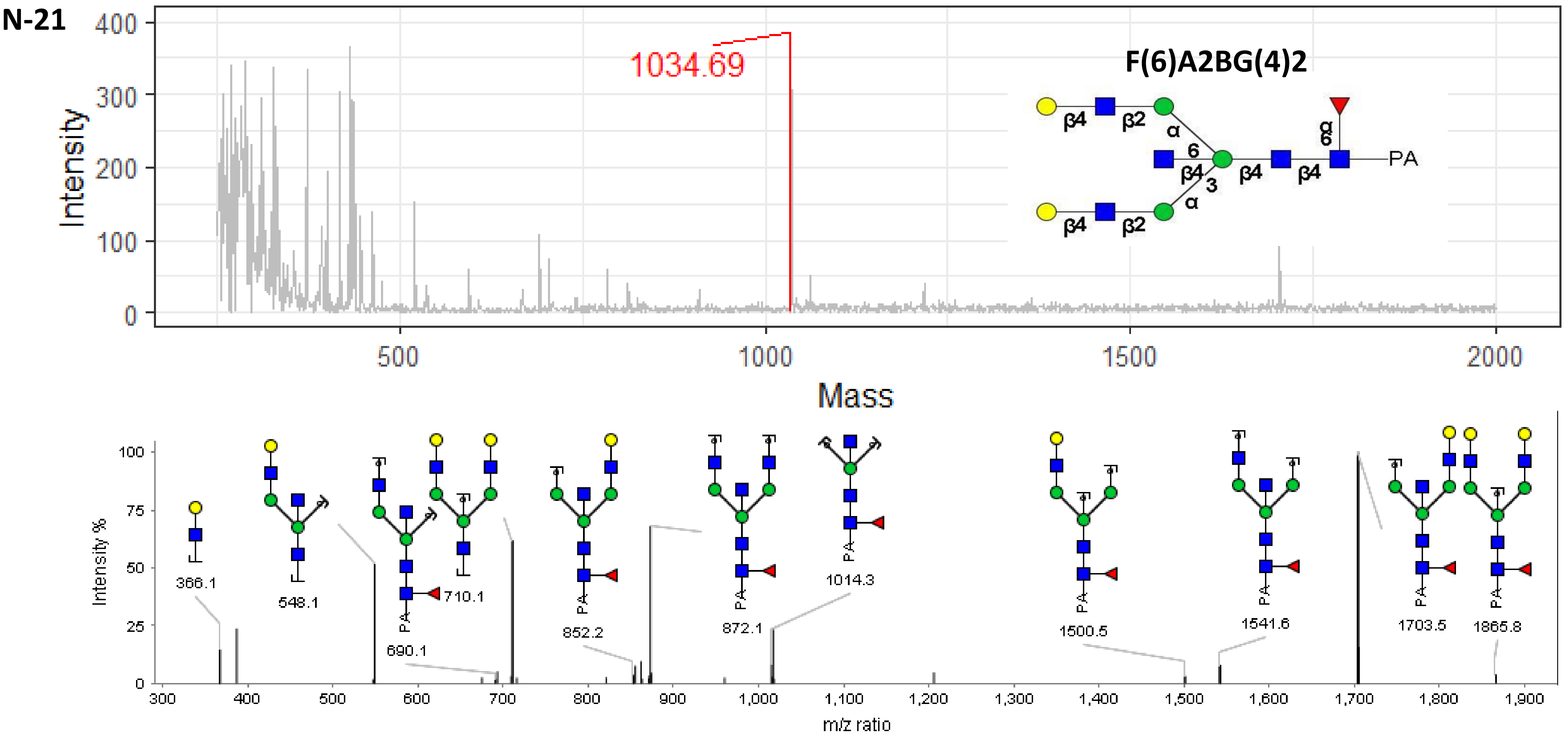

400
300
200
100
0
Intensity
1034.69
F(6)A2BG(4)2
β4
β2
α
6
3
PA
500
1000
1500
2000
Mass
100
75
50
25
0
Intensity %
366.1
548.1
690.1
710.1
852.2
872.1
1014.3
1500.5
1541.6
1703.5
1865.8
300
400
600
700
800
900
1,000
1,100
1,200
1,300
1,400
1,500
1,600
1,700
1,800
1,900
m/z ratio

A1-2-1

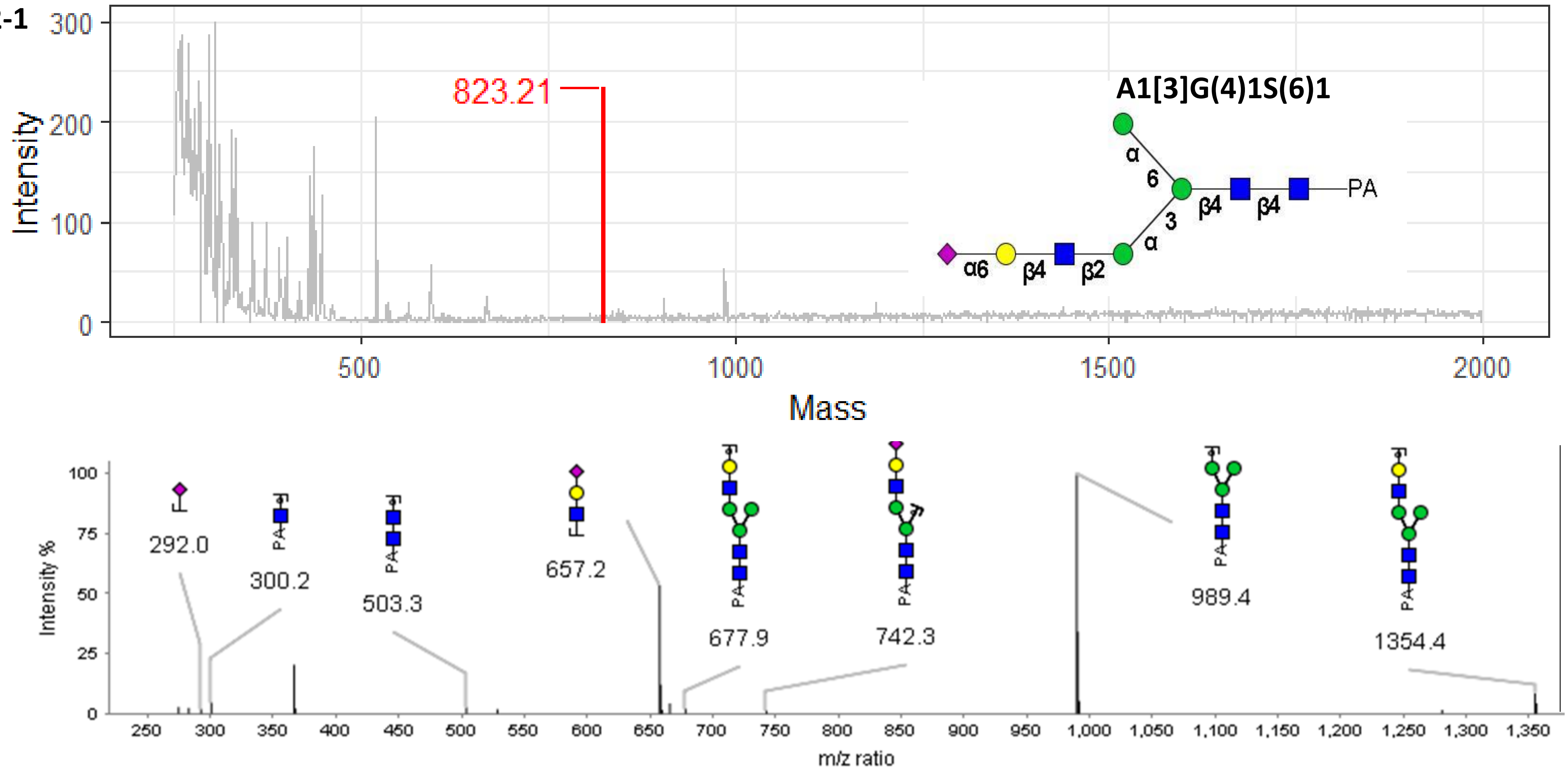

300
200
100
0
Intensity
823.21
A1[3]G(4)1S(6)1
α
6
3
α
β4
β4
PA
α6
β4
β2
500
1000
1500
2000
Mass
100
75
50
25
0
Intensity %
292.0
300.2
503.3
657.2
677.9
742.3
989.4
1354.4
250
300
350
400
450
500
550
600
650
700
750
800
850
900
950
1,000
1,050
1,100
1,150
1,200
1,250
1,300
1,350
m/z ratio

A1-2-2

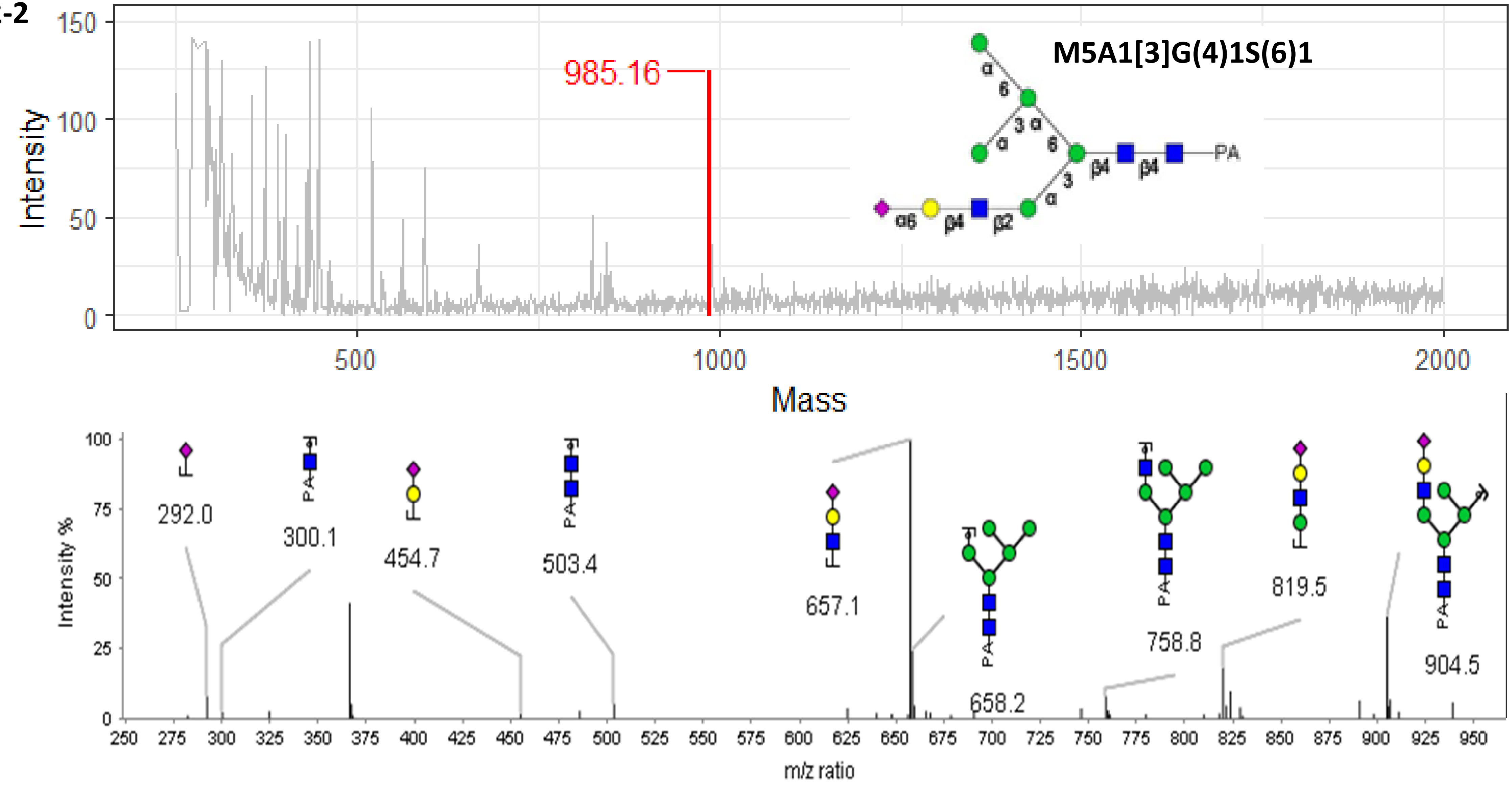

M5A1[3]G(4)1S(6)1
985.16
Intensity
Mass
Intensity %
m/z ratio
292.0
300.1
454.7
503.4
657.1
658.2
758.8
819.5
904.5

A1-3

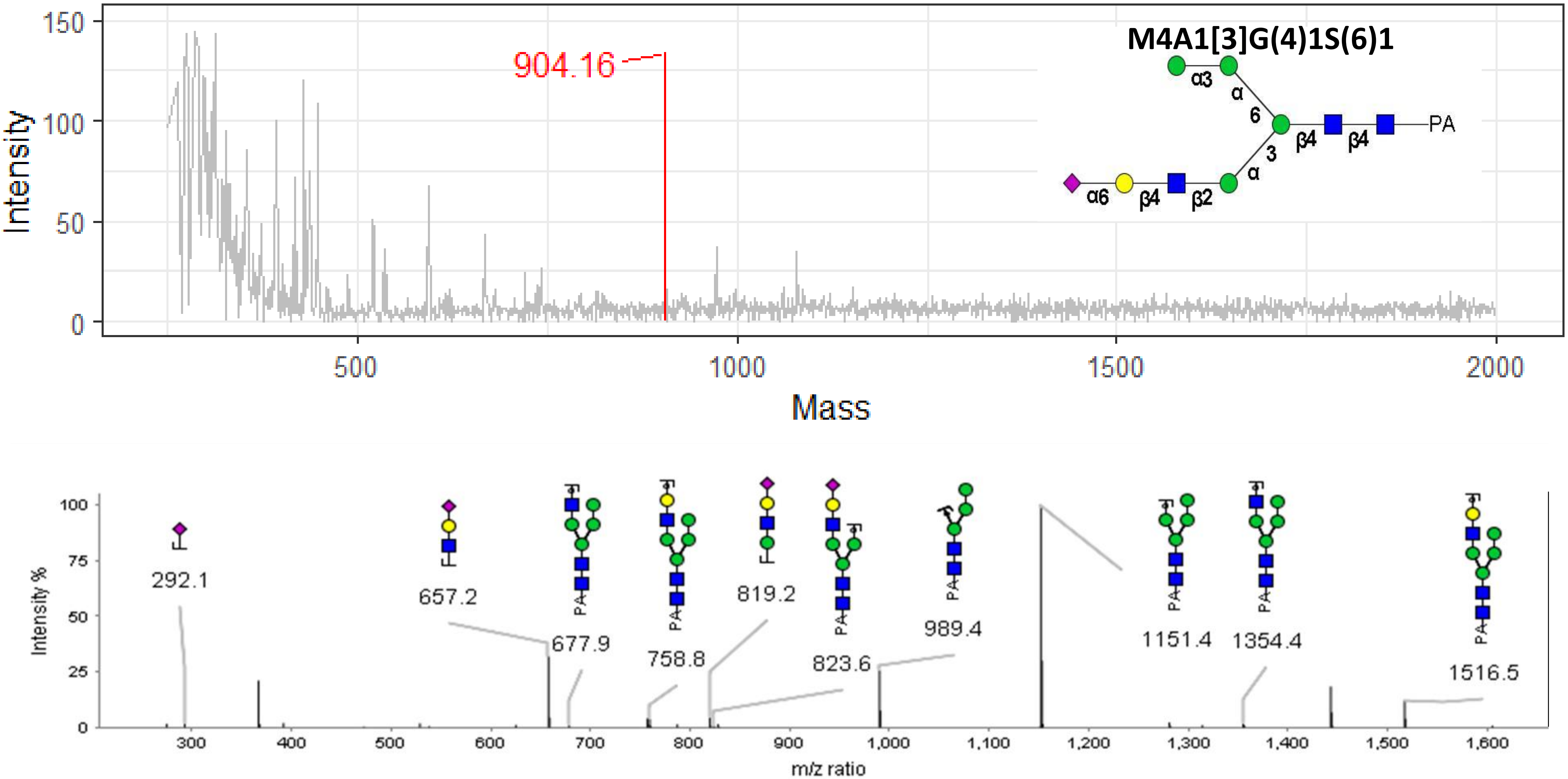
M4A1[3]G(4)1S(6)1
904.16
Intensity
Mass
PA
292.1
657.2
677.9
758.8
819.2
823.6
989.4
1151.4
1354.4
1516.5
Intensity %
m/z ratio

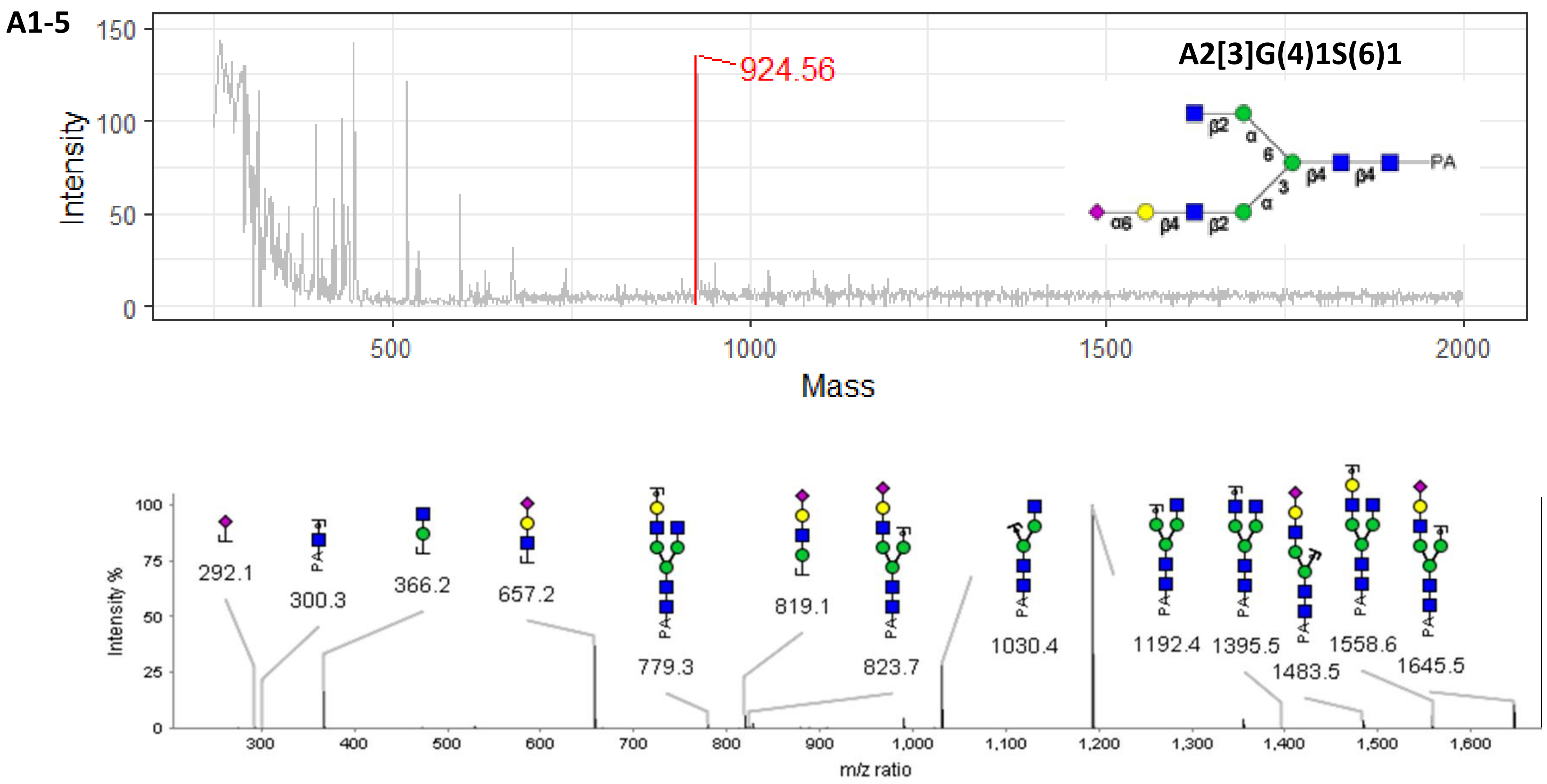
A1-5
150
100
50
0
Intensity
924.56
A2[3]G(4)1S(6)1
β2
α
6
3
β4
β4
PA
α6
β4
β2
α
500
1000
1500
2000
Mass
100
75
50
25
0
Intensity %
292.1
300.3
366.2
657.2
779.3
819.1
823.7
1030.4
1192.4
1395.5
1483.5
1558.6
1645.5
PA
300
400
500
600
700
800
900
1,000
1,100
1,200
1,300
1,400
1,500
1,600
m/z ratio

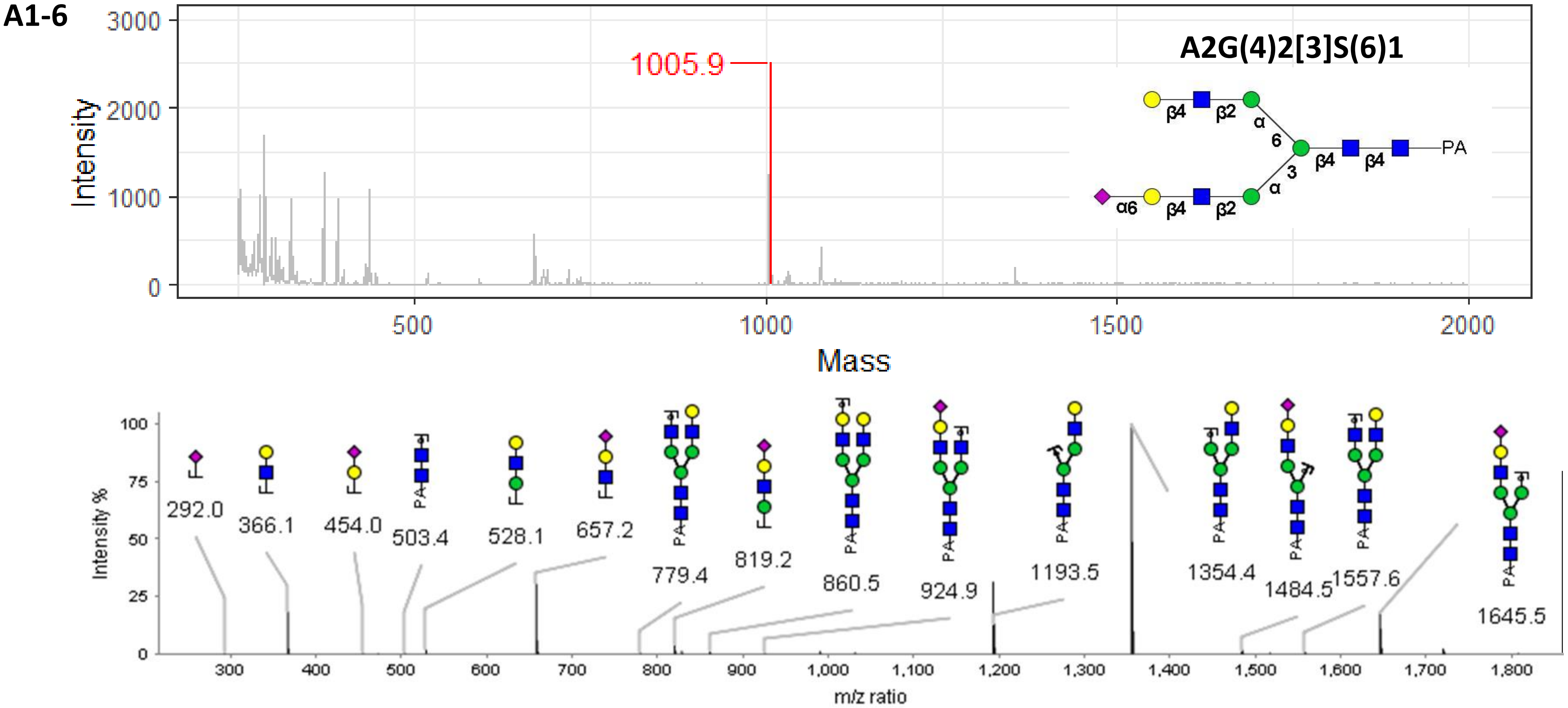
A1-6
3000
2000
1000
0
Intensity
1005.9
A2G(4)2[3]S(6)1
β4
β2
α
6
3
α6
PA
500
1000
1500
2000
Mass
100
75
50
25
0
Intensity %
292.0
366.1
454.0
503.4
528.1
657.2
779.4
819.2
860.5
924.9
1193.5
1354.4
1484.5
1557.6
1645.5
300
400
500
600
700
800
900
1,000
1,100
1,200
1,300
1,400
1,500
1,600
1,700
1,800
m/z ratio

A1-9

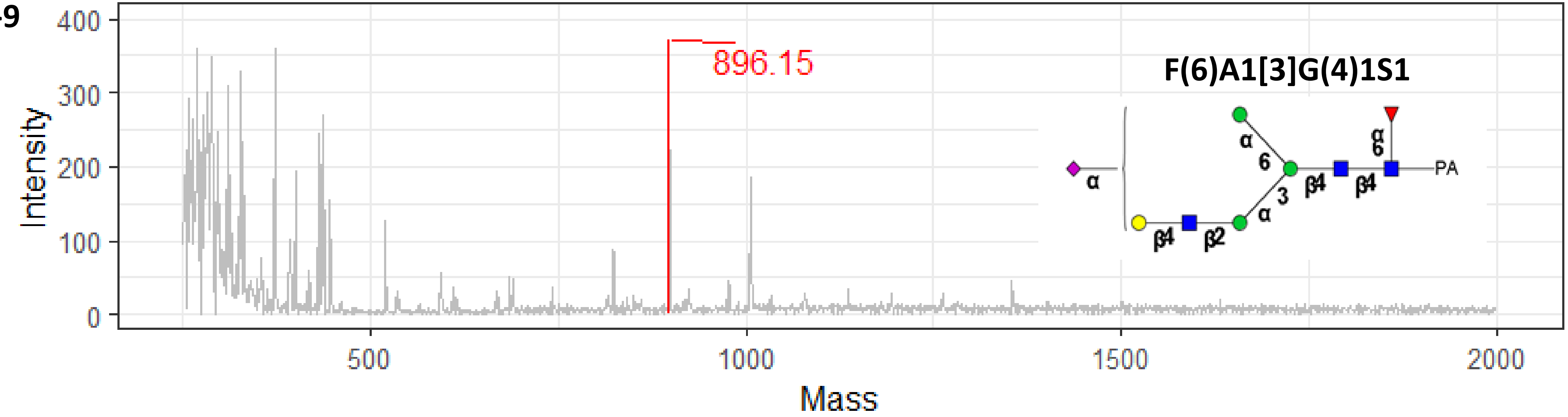

400
300
200
100
0
Intensity
896.15
F(6)A1[3]G(4)1S1
PA
500
1000
1500
2000
Mass

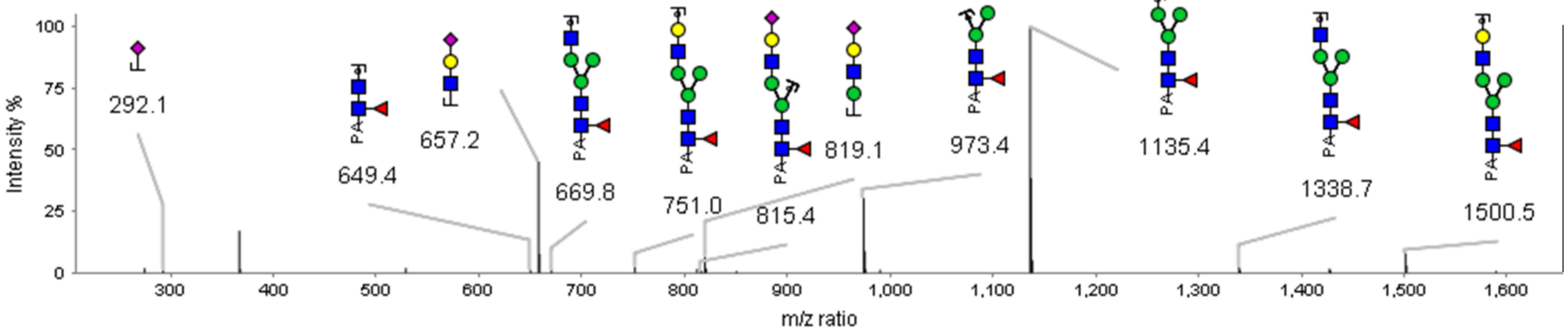

100
75
50
25
0
Intensity %
292.1
649.4
657.2
669.8
751.0
815.4
819.1
973.4
1135.4
1338.7
1500.5
300
400
500
600
700
800
900
1,000
1,100
1,200
1,300
1,400
1,500
1,600
m/z ratio

A1-12

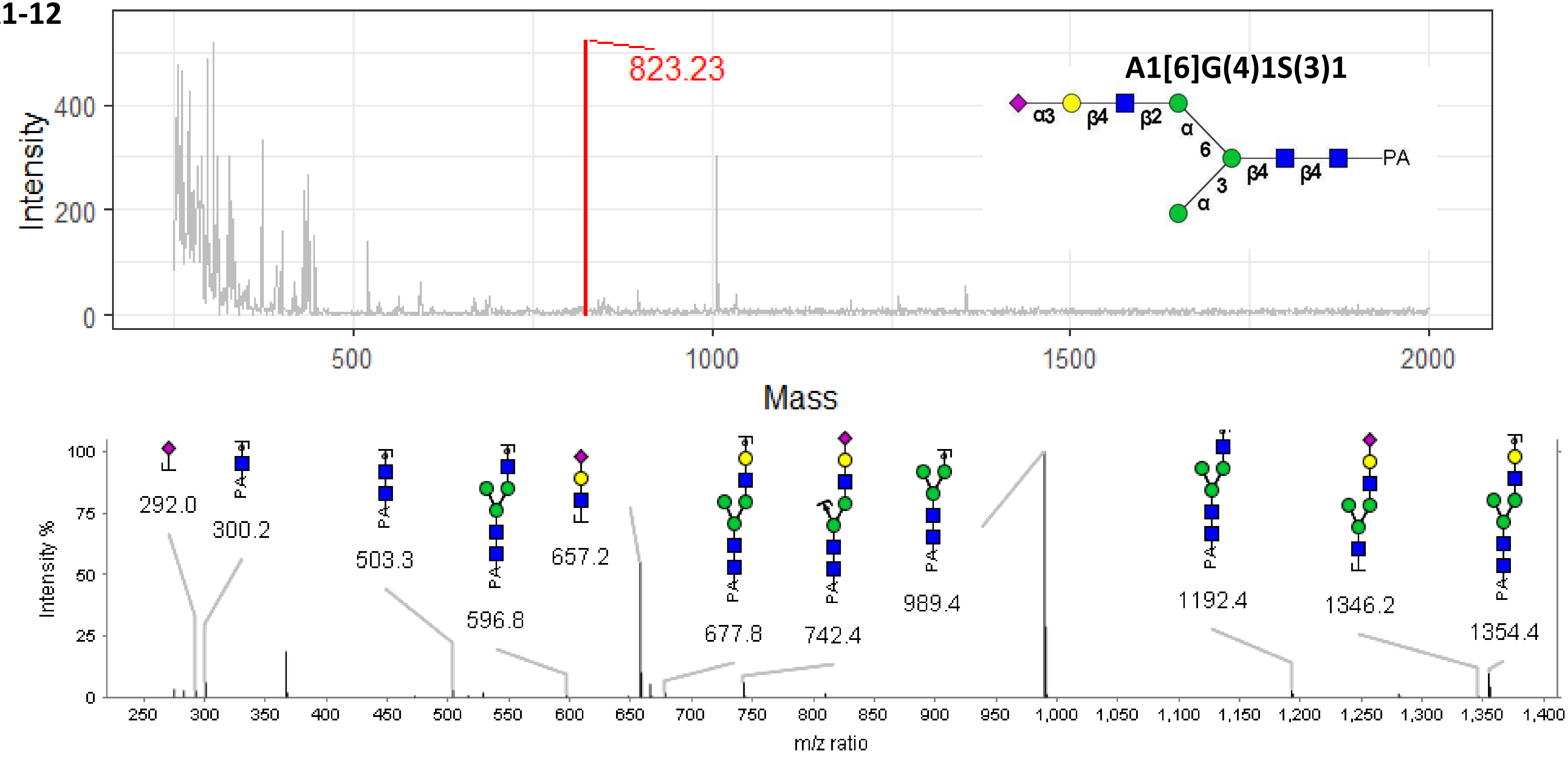
823.23
A1[6]G(4)1S(3)1
α3
β4
β2
α
6
3
α
β4
β4
PA
Intensity
400
200
0
500
1000
1500
2000
Mass
292.0
300.2
503.3
596.8
657.2
677.8
742.4
989.4
1192.4
1346.2
1354.4
Intensity %
100
75
50
25
0
m/z ratio

A1-14-2

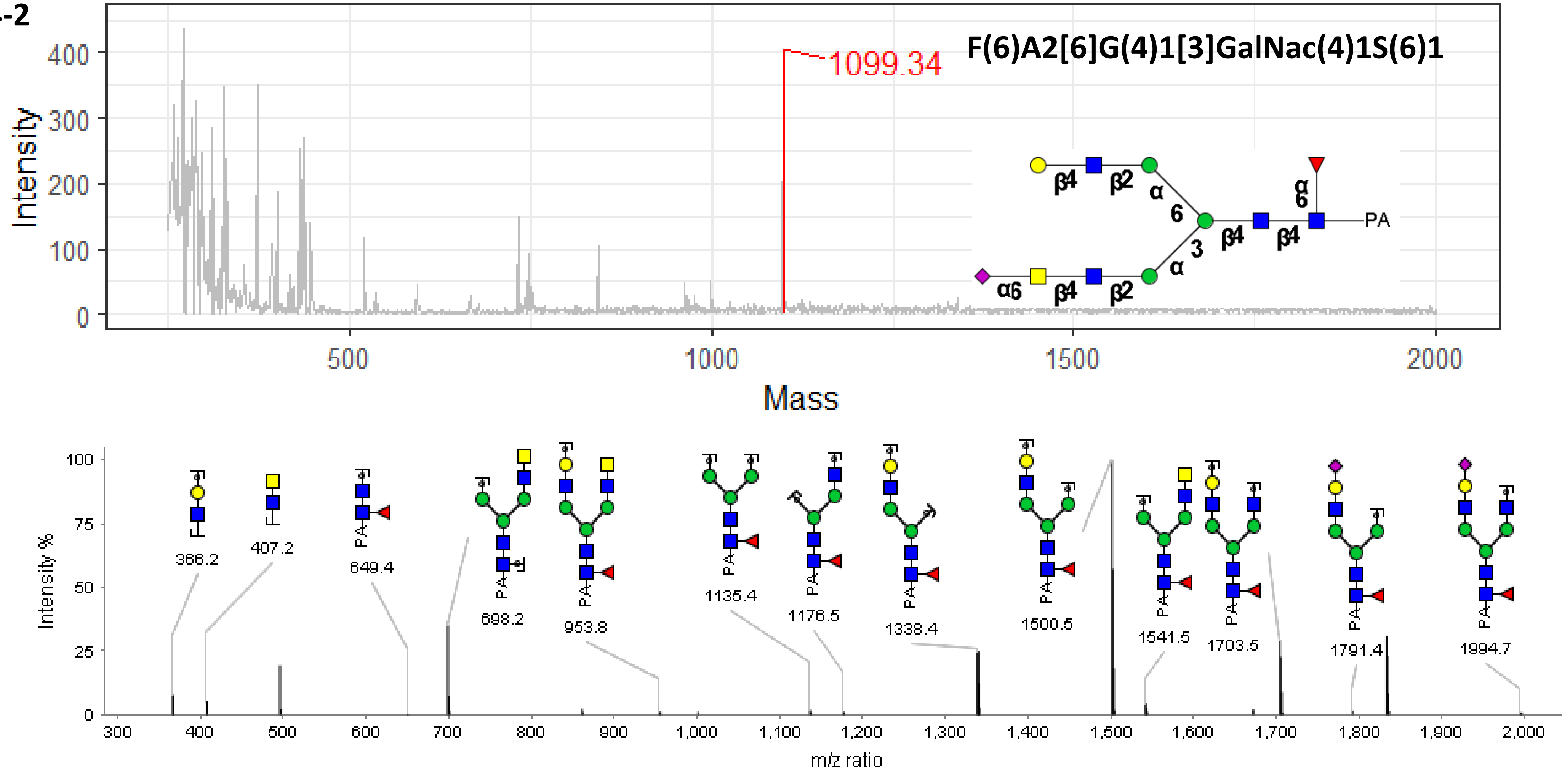

F(6)A2[6]G(4)1[3]GalNac(4)1S(6)1
1099.34
Intensity
Mass
400
300
200
100
0
500
1000
1500
2000
β4
β2
α
6
3
α6
PA
Intensity %
m/z ratio
366.2
407.2
649.4
698.2
953.8
1135.4
1176.5
1338.4
1500.5
1541.5
1703.5
1791.4
1994.7

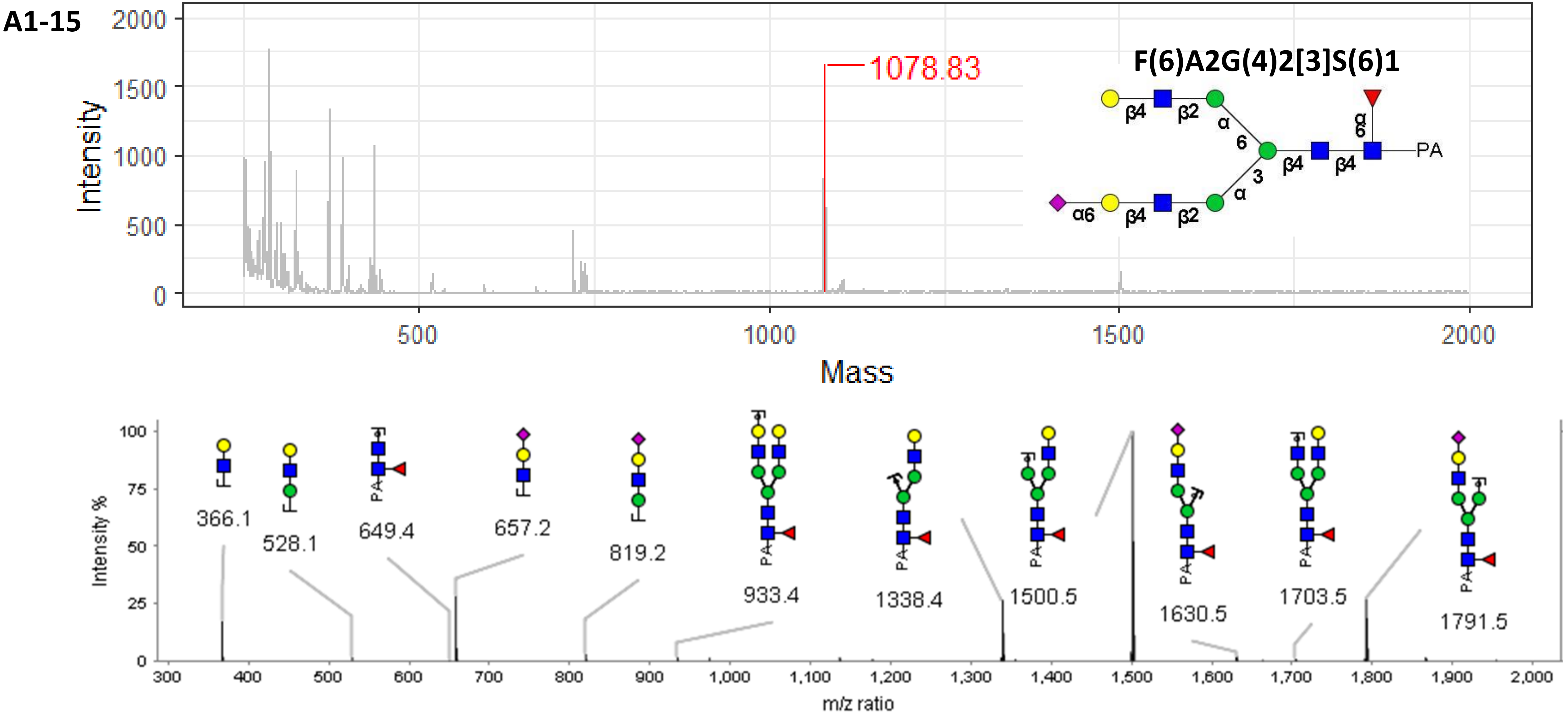
A1-15
Intensity
Mass
1078.83
F(6)A2G(4)2[3]S(6)1
PA
Intensity %
m/z ratio
366.1
528.1
649.4
657.2
819.2
933.4
1338.4
1500.5
1630.5
1703.5
1791.5

A1-17

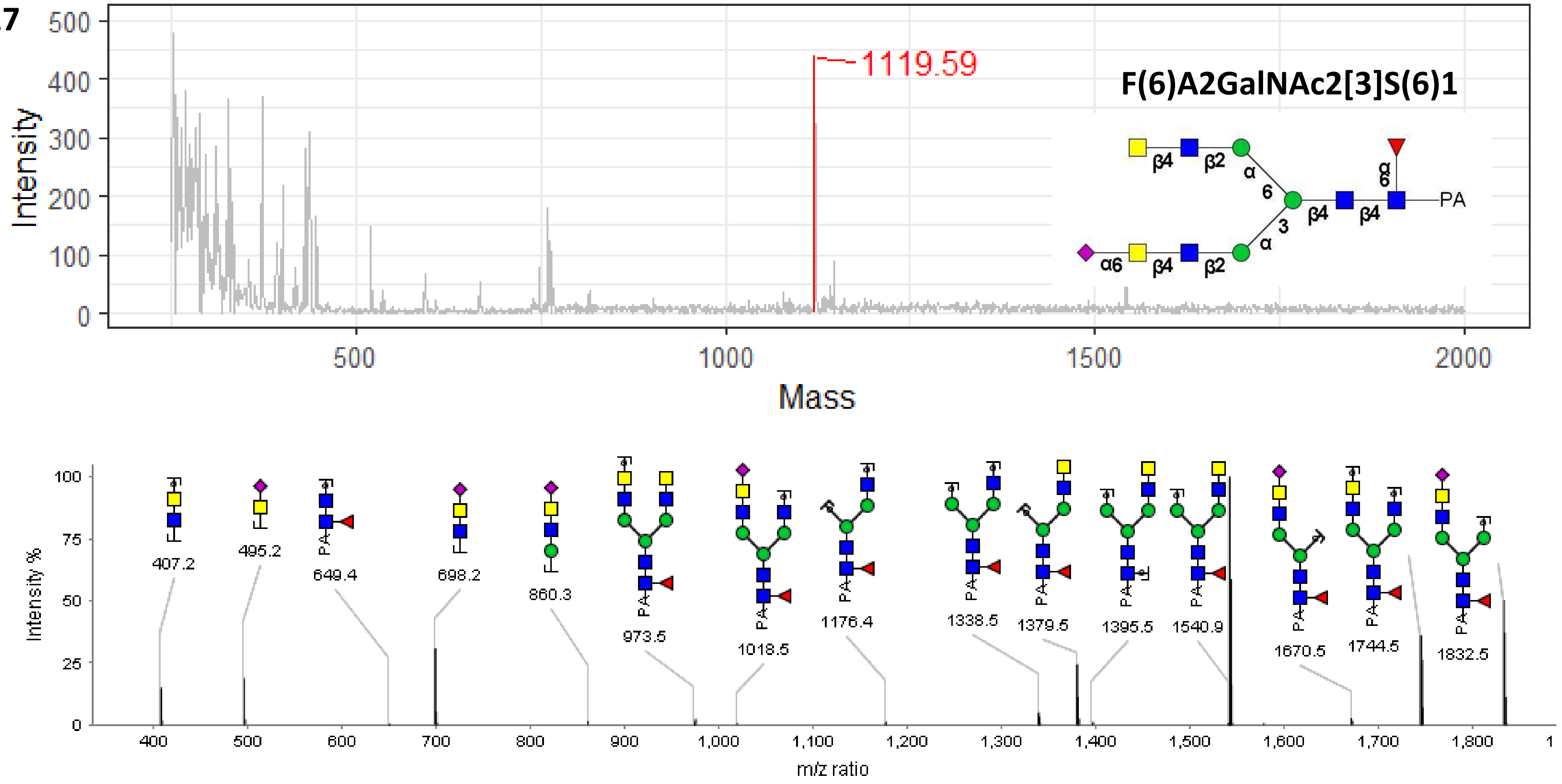

F(6)A2GalNAc2[3]S(6)1
1119.59
Intensity
Mass
PA
Intensity %
m/z ratio
407.2
495.2
649.4
698.2
860.3
973.5
1018.5
1176.4
1338.5
1379.5
1395.5
1540.9
1670.5
1744.5
1832.5

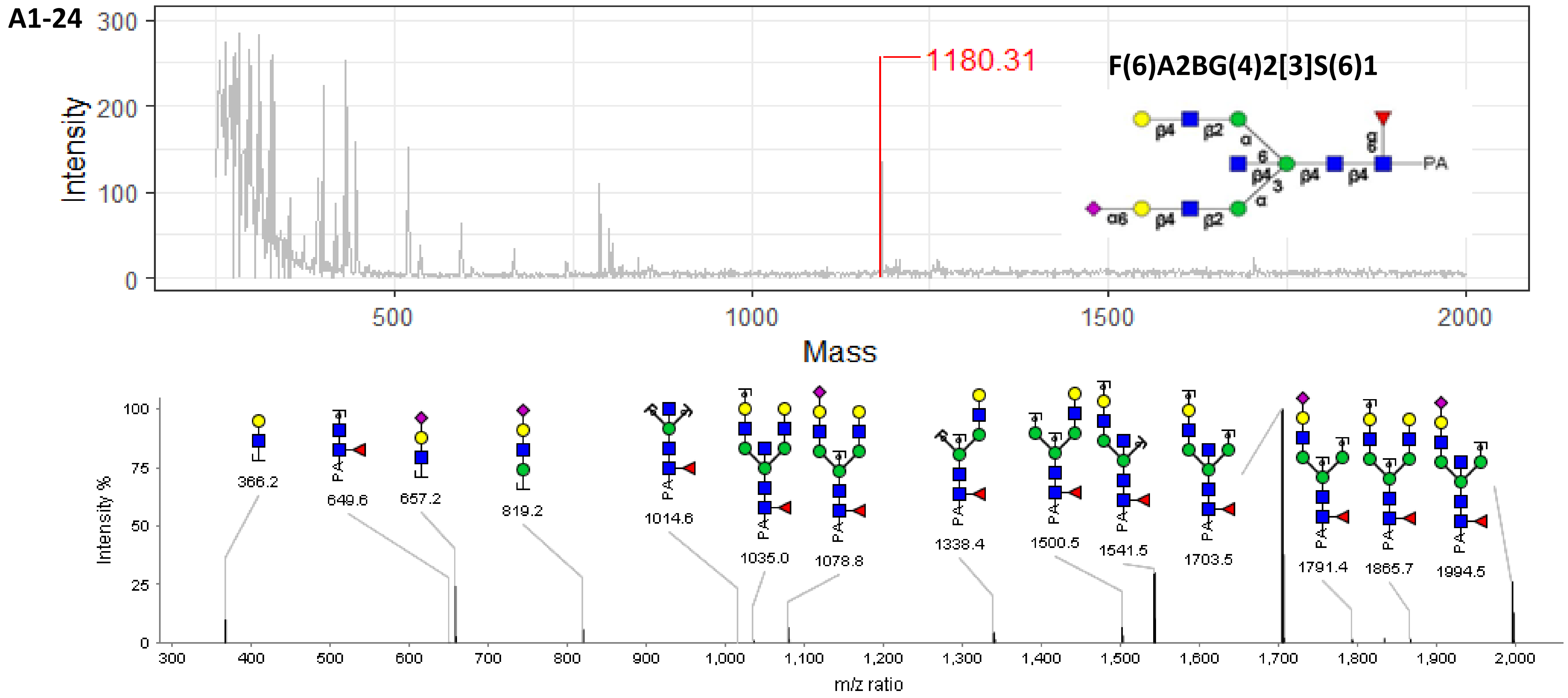

A1-24
300
200
100
0
Intensity
500
1000
1500
2000
Mass
1180.31
F(6)A2BG(4)2[3]S(6)1
PA
100
75
50
25
0
Intensity %
366.2
649.6
657.2
819.2
1014.6
1035.0
1078.8
1338.4
1500.5
1541.5
1703.5
1791.4
1865.7
1994.5
300
400
500
600
700
800
900
1,000
1,100
1,200
1,300
1,400
1,500
1,600
1,700
1,800
1,900
2,000
m/z ratio

A2-2

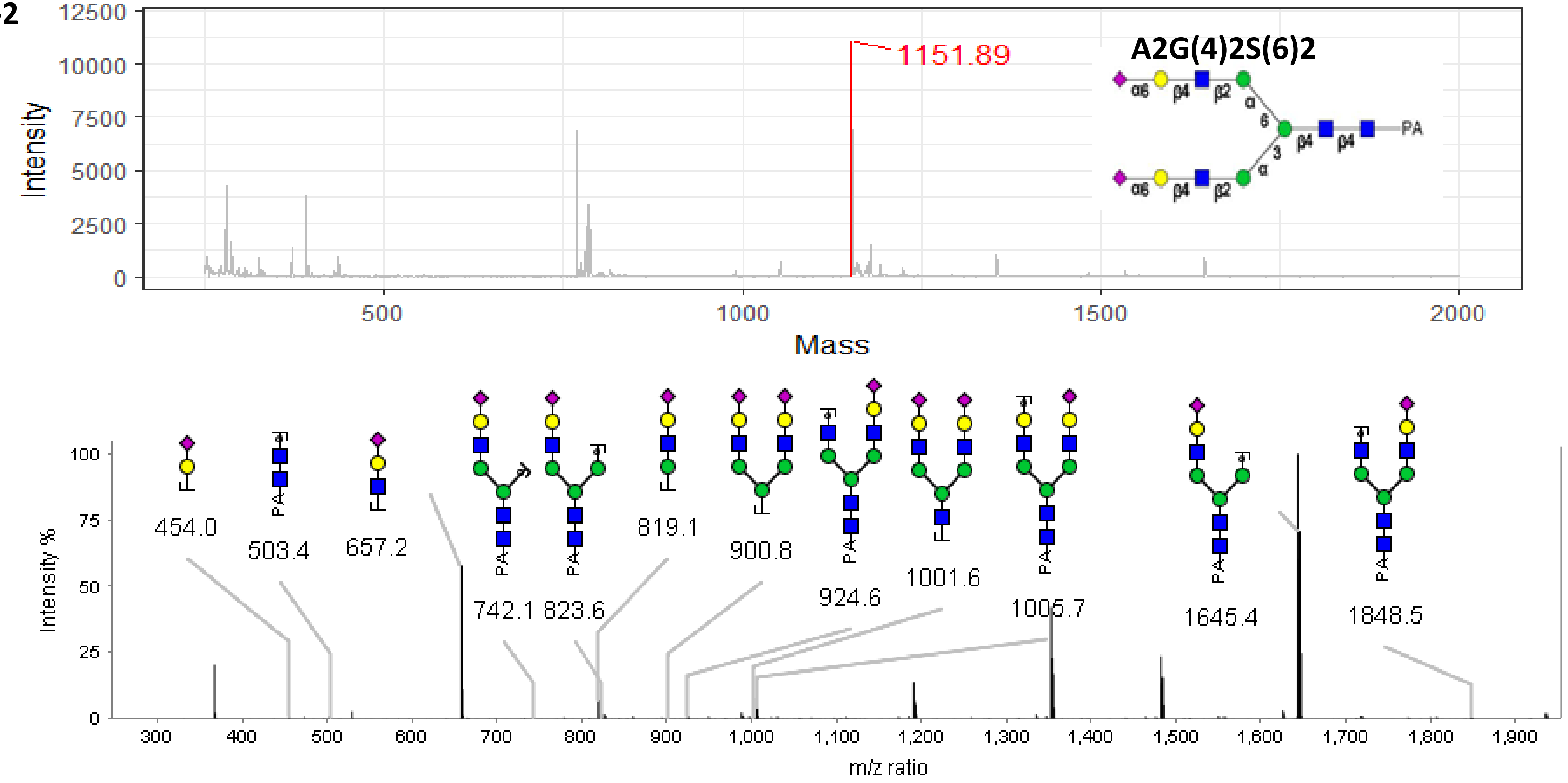

12500
10000
7500
5000
2500
0
Intensity
1151.89
A2G(4)2S(6)2
α6
β4
β2
α
6
3
PA
500
1000
1500
2000
Mass
100
75
50
25
0
Intensity %
454.0
503.4
657.2
742.1
823.6
819.1
900.8
924.6
1001.6
1005.7
1645.4
1848.5
300
400
500
600
700
800
900
1,000
1,100
1,200
1,300
1,400
1,500
1,600
1,700
1,800
1,900
m/z ratio

A2-8

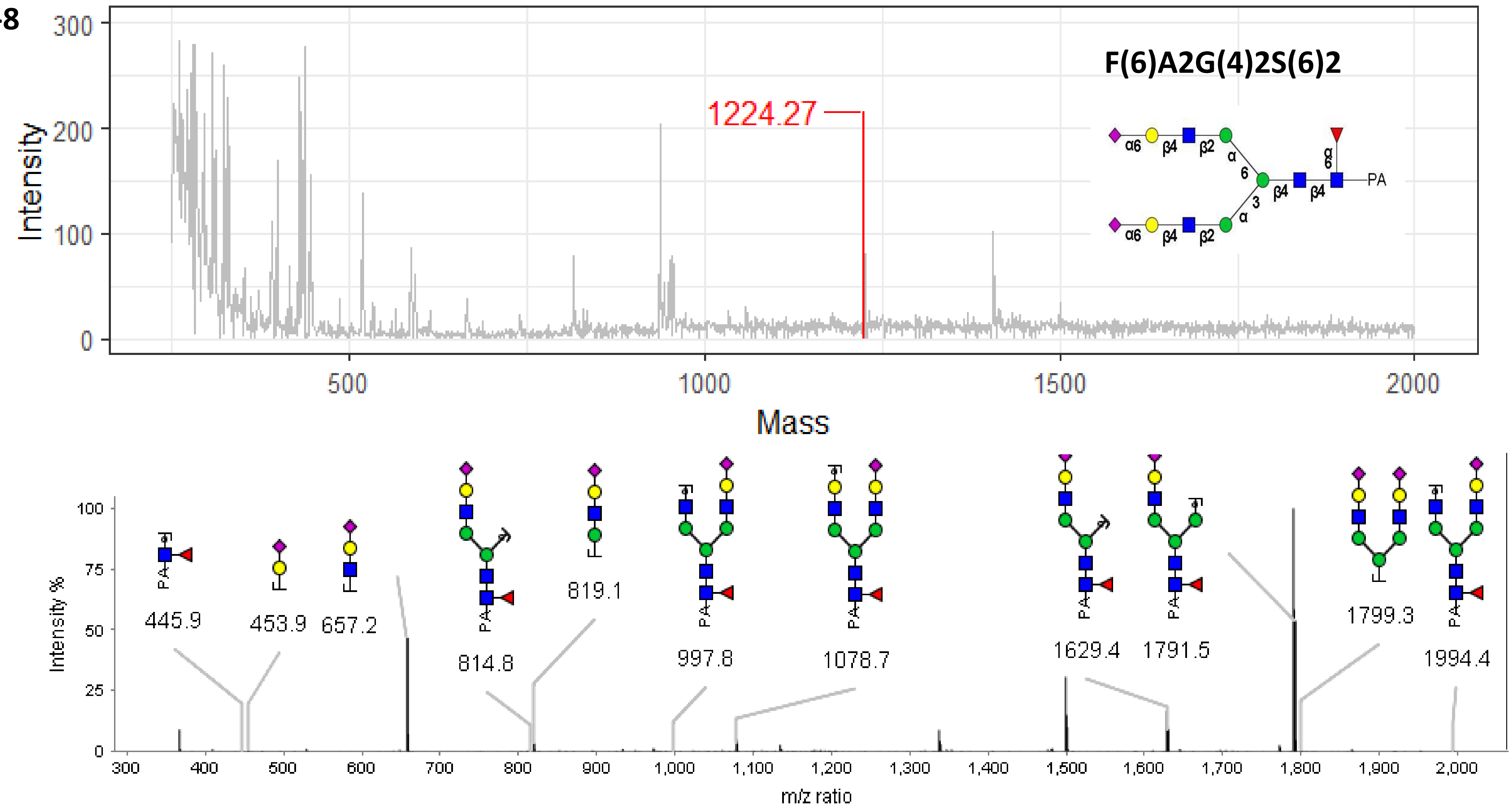

F(6)A2G(4)2S(6)2
1224.27
Intensity
Mass
PA
445.9
453.9
657.2
814.8
819.1
997.8
1078.7
1629.4
1791.5
1799.3
1994.4
Intensity %
m/z ratio

A2-9

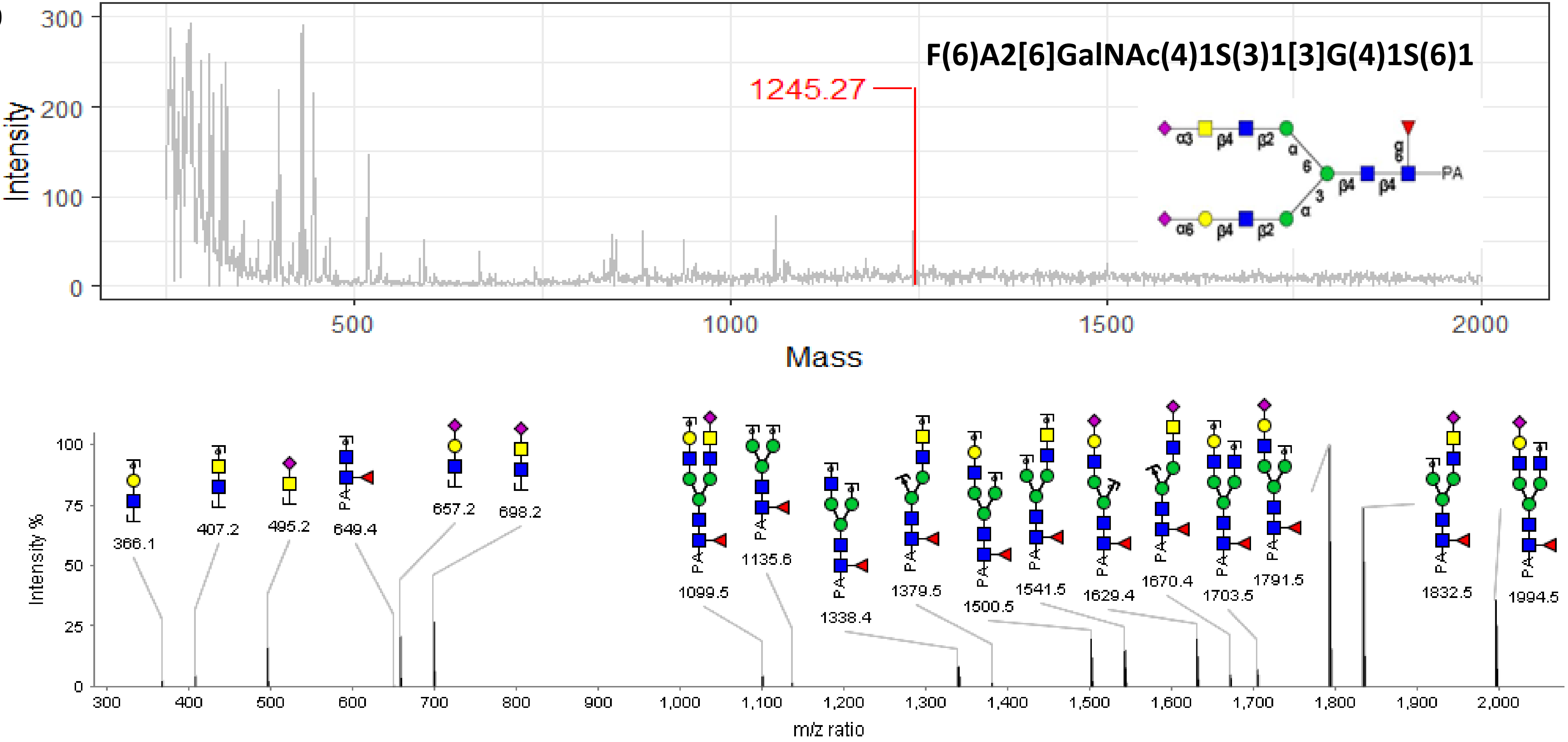
F(6)A2[6]GalNAc(4)1S(3)1[3]G(4)1S(6)1
1245.27
Intensity
300
200
100
0
500
1000
1500
2000
Mass
α3
β4
β2
α6
β4
β2
α
6
α
3
β4
β4
α6
PA
Intensity %
100
75
50
25
0
366.1
407.2
495.2
649.4
657.2
698.2
1099.5
1135.6
1338.4
1379.5
1500.5
1541.5
1629.4
1670.4
1703.5
1791.5
1832.5
1994.5
300
400
500
600
700
800
900
1,000
1,100
1,200
1,300
1,400
1,500
1,600
1,700
1,800
1,900
2,000
m/z ratio

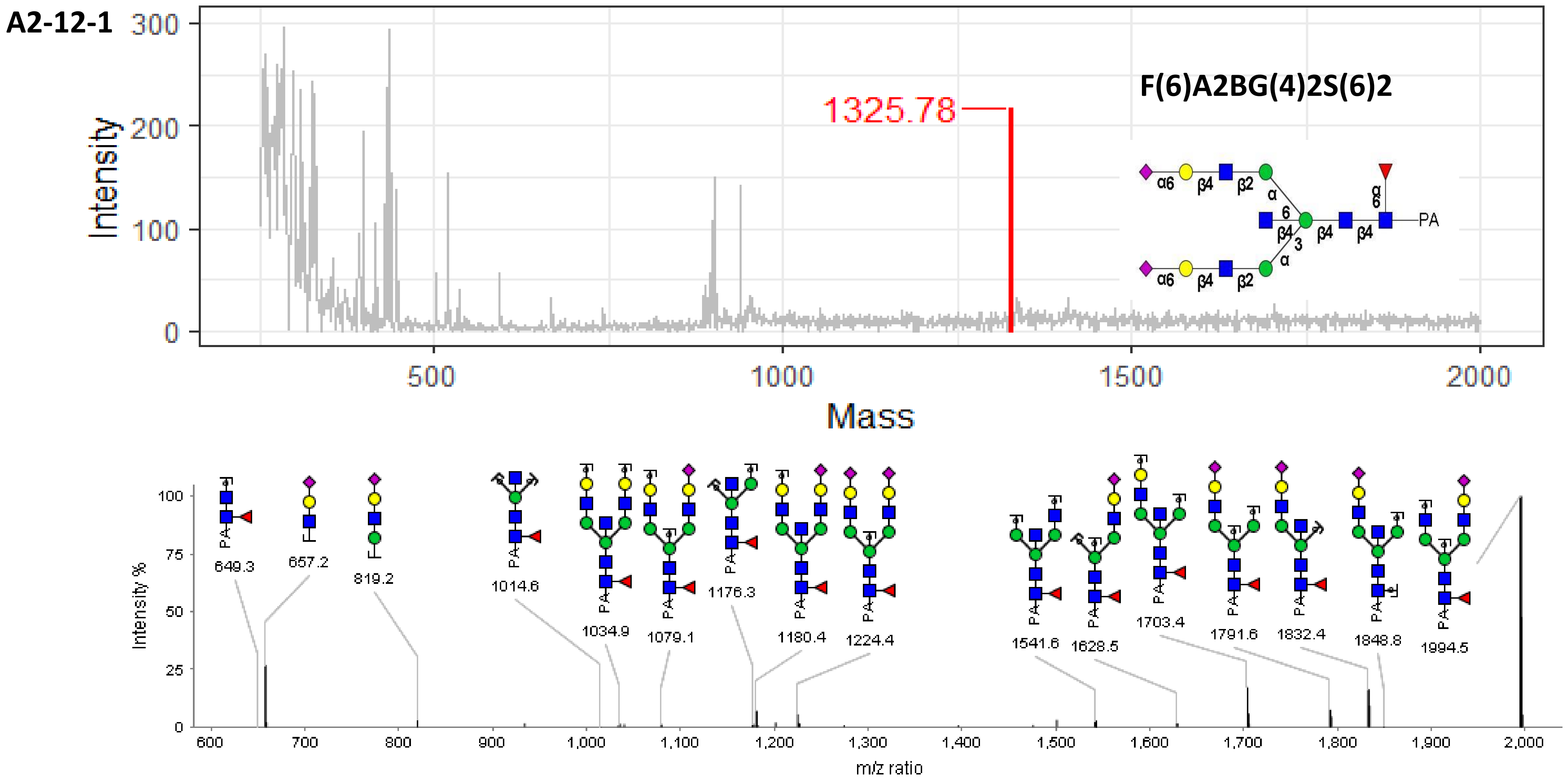
A2-12-1
Intensity
Mass
1325.78
F(6)A2BG(4)2S(6)2
PA
649.3
657.2
819.2
1014.6
1034.9
1079.1
1176.3
1180.4
1224.4
1541.6
1628.5
1703.4
1791.6
1832.4
1848.8
1994.5
Intensity %
m/z ratio

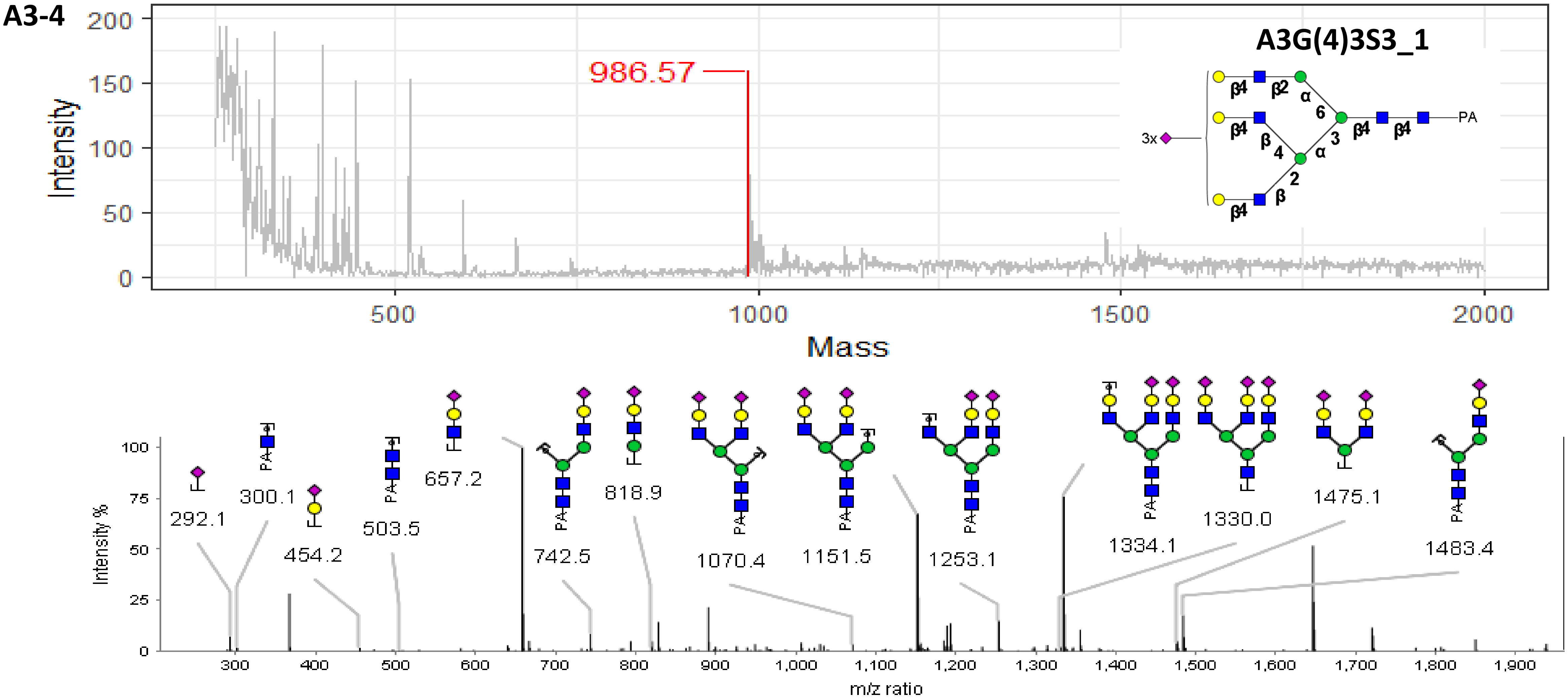

A3-4
A3G(4)3S3_1
986.57
Intensity
Mass
3x
PA
β4
β2
α
6
3
β
4
2
292.1
300.1
454.2
503.5
657.2
742.5
818.9
1070.4
1151.5
1253.1
1334.1
1330.0
1475.1
1483.4
Intensity %
m/z ratio

A3-13

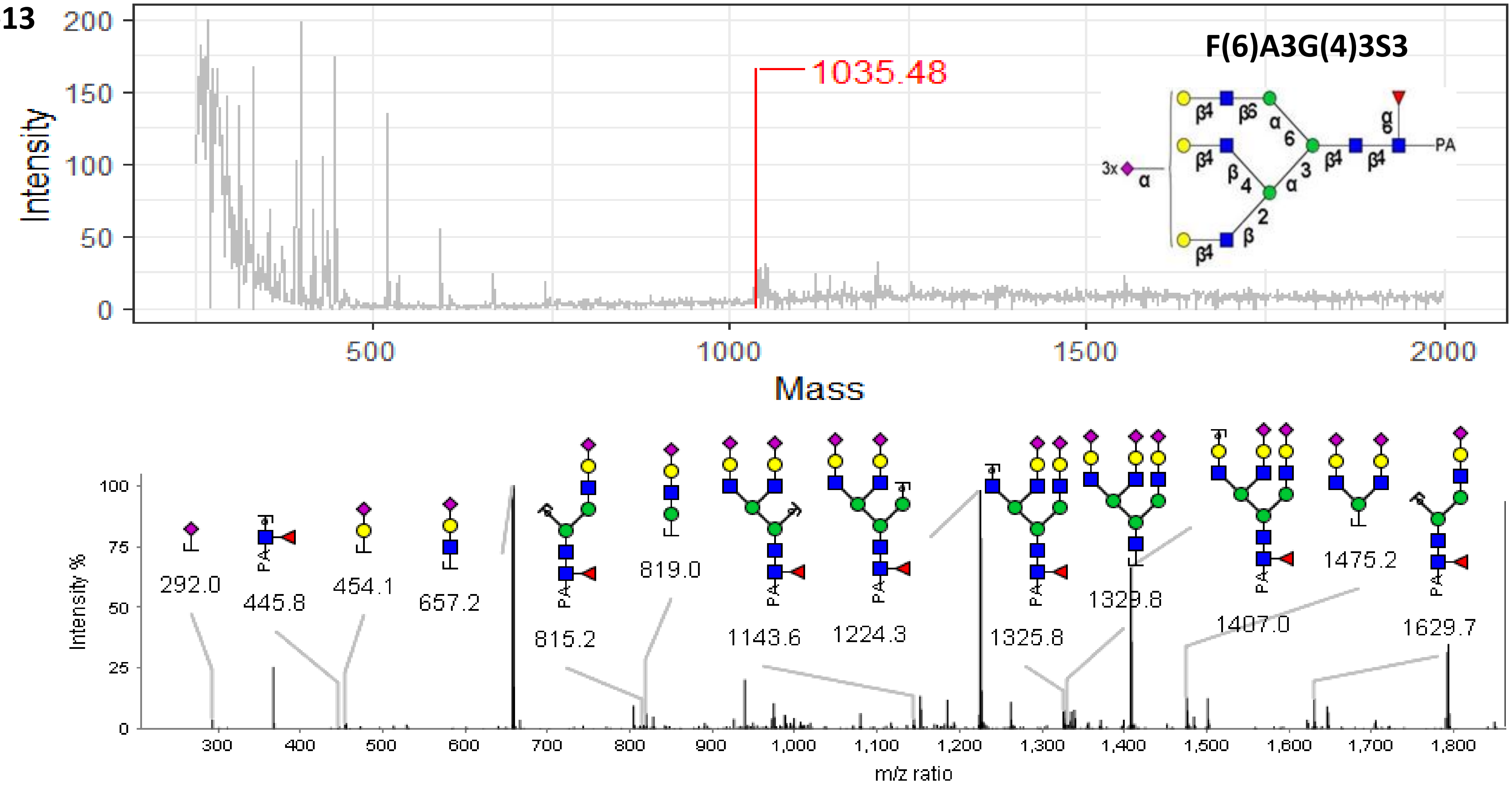

F(6)A3G(4)3S3
1035.48
Intensity
Mass
200
150
100
50
0
500
1000
1500
2000
3x
PA
292.0
445.8
454.1
657.2
815.2
819.0
1143.6
1224.3
1325.8
1329.8
1407.0
1475.2
1629.7
Intensity %
m/z ratio

A4-5

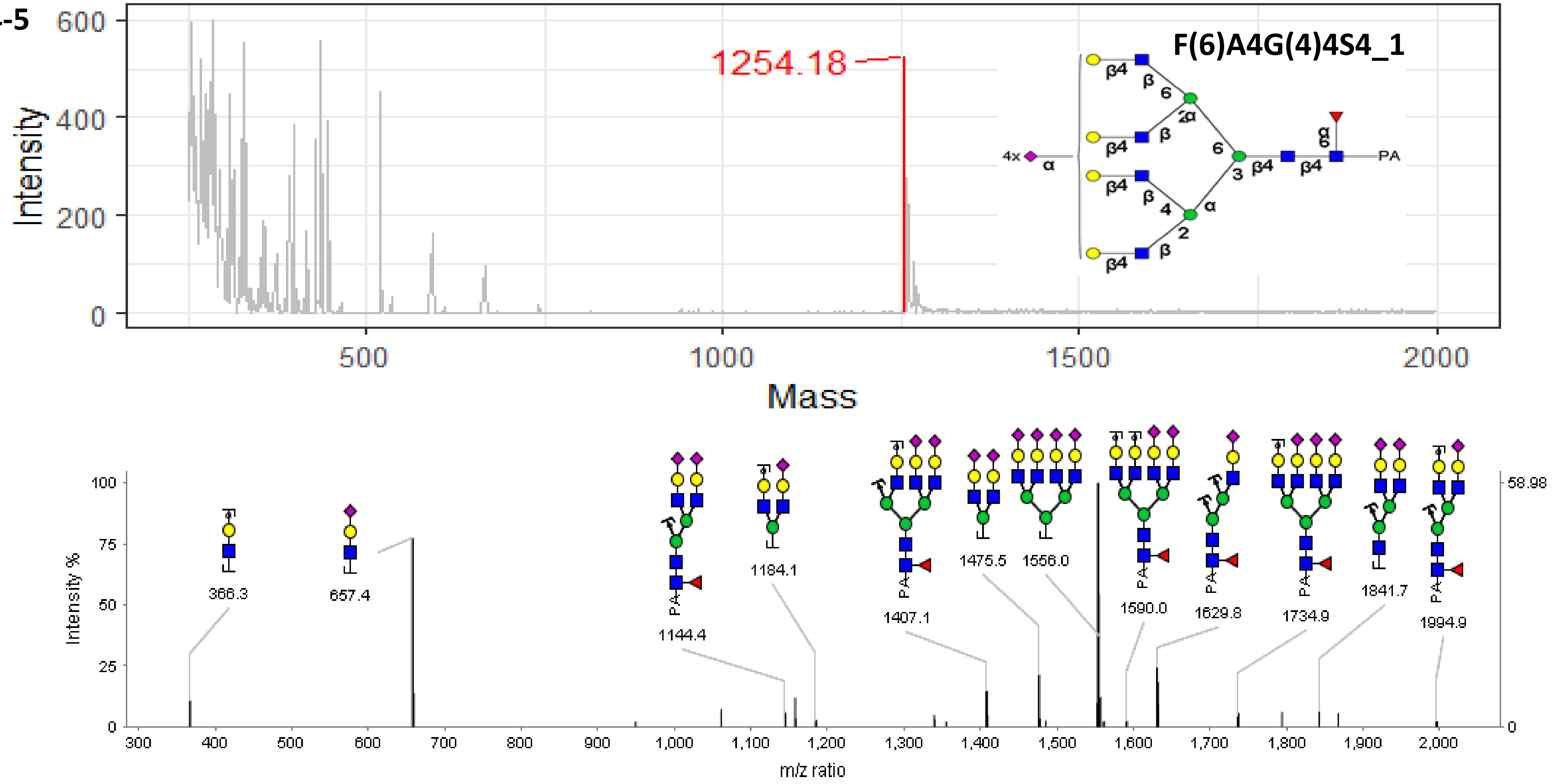
F(6)A4G(4)4S4_1
1254.18
Intensity
Mass
600
400
200
0
500
1000
1500
2000
4x
PA
Intensity %
m/z ratio
366.3
657.4
1144.4
1184.1
1407.1
1475.5
1556.0
1590.0
1629.8
1734.9
1841.7
1994.9
58.98
