# Supplementary material for: Microheterogeneity and Individual Differences of Human Urinary N-Glycome under Normal Physiological Conditions
Source: Biomolecules. 2023 Apr 27;13(5):756. doi: 10.3390/biom13050756 (PMC10216293; doi:10.3390/biom13050756)

Figure S1. Anion exchange HPLC of human urine *N*-glycans.

The result for the sample 036 is presented as a representative example. Other samples showed similar profiles. The peaks indicated by N, A1 to A4 were fractionated as a neutral, monoanionic, dianionic, trianionic, and tetraanionic PA-glycans, respectively. The arrowheads from S0 to S4 indicate the elution positions of standard *N*-glycans prepared from α1-acid glycoproteins, and these numbers correspond to the number of sialic acids. The mark x shows a non-glycan peak.

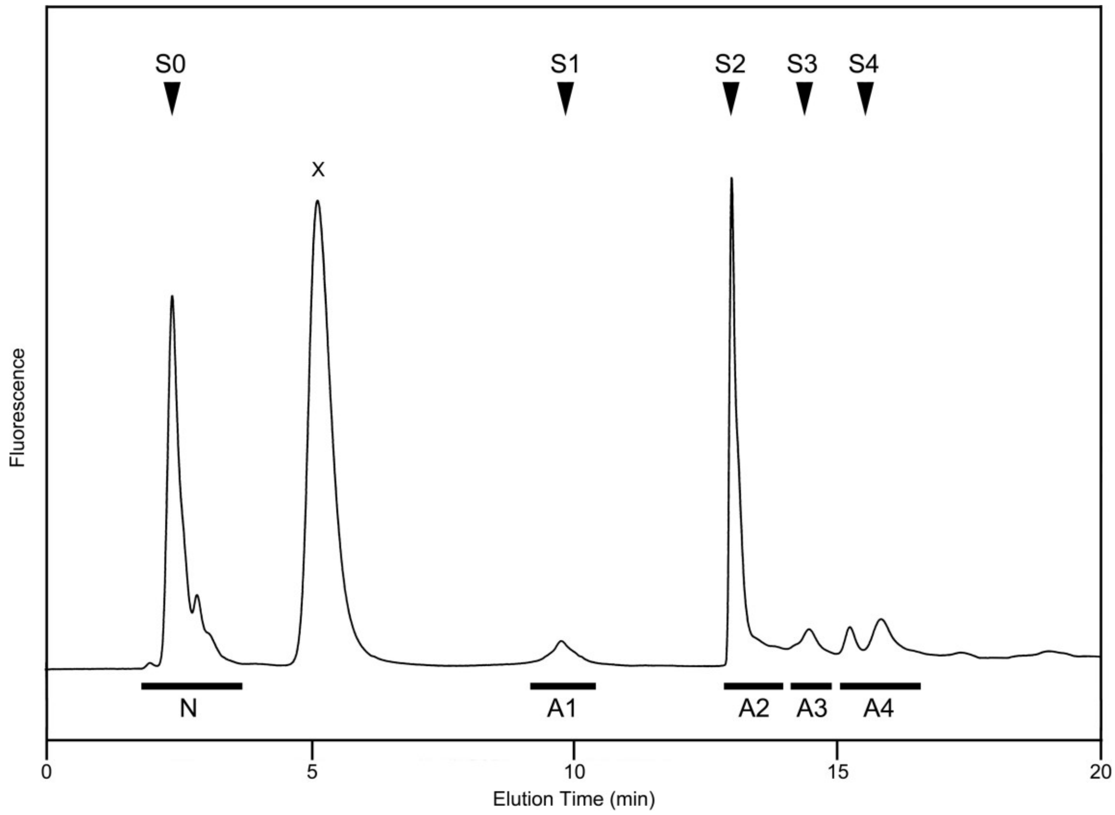

Supplement: Supplementary file 1 [file biomolecules-13-00756-s001.zip › suplementry data/Figure_S1.pdf]
