# Supplementary material for: Microheterogeneity and Individual Differences of Human Urinary N-Glycome under Normal Physiological Conditions
Source: Biomolecules. 2023 Apr 27;13(5):756. doi: 10.3390/biom13050756 (PMC10216293; doi:10.3390/biom13050756)

Figure S2. Chromatograms of reversed phase HPLC for each sample.

Each fraction (N, A1, A2, A3, A4) separated on anion exchange HPLC were developed further on reversed phase HPLC. The peaks were colored with 2 different colors; red color for the peaks detected in at least 10 in 12 samples (Core glycome), blue color for the peaks detected in less than 10 samples. The peaks in each fraction was labeled by numbers referring to certain identified glycan. All identification and quantification data for these labeled peaks can be shown in Table S2. The unlabeled peaks were considered as PA derivatives of *O*-glycans, glucose oligomers or artificial products from *N*-glycans.

028

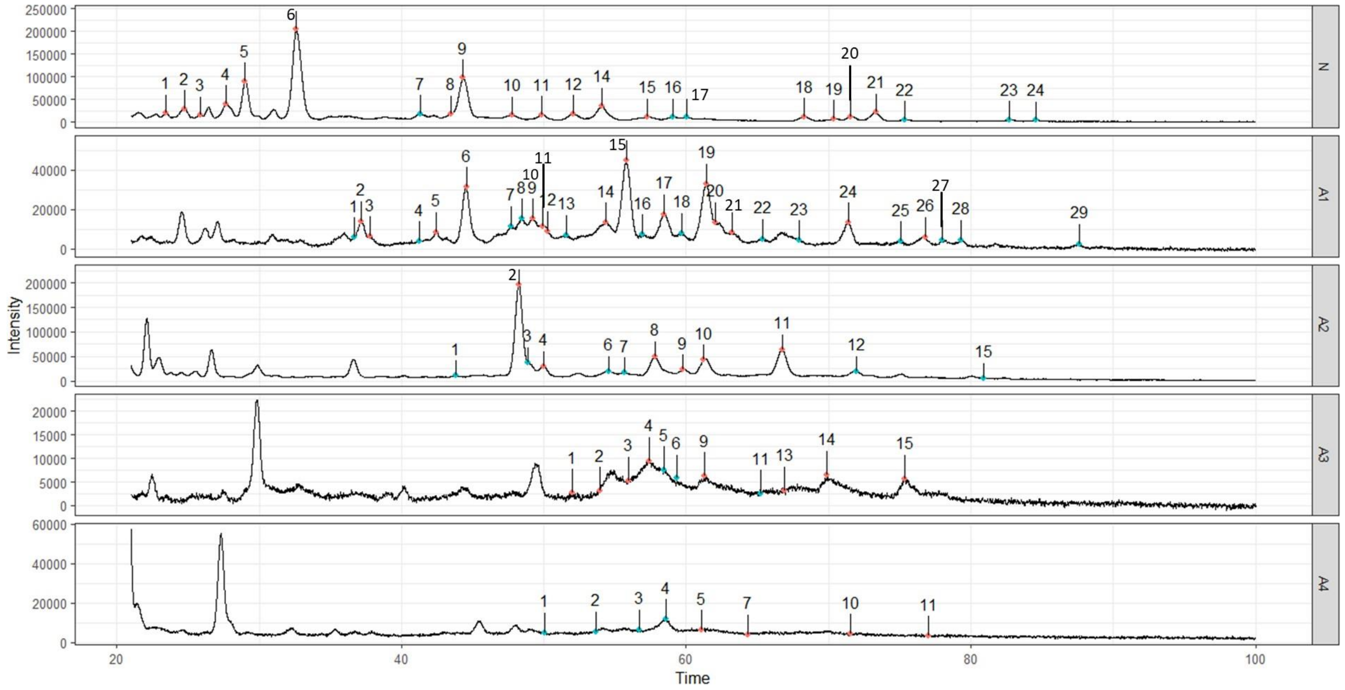

031

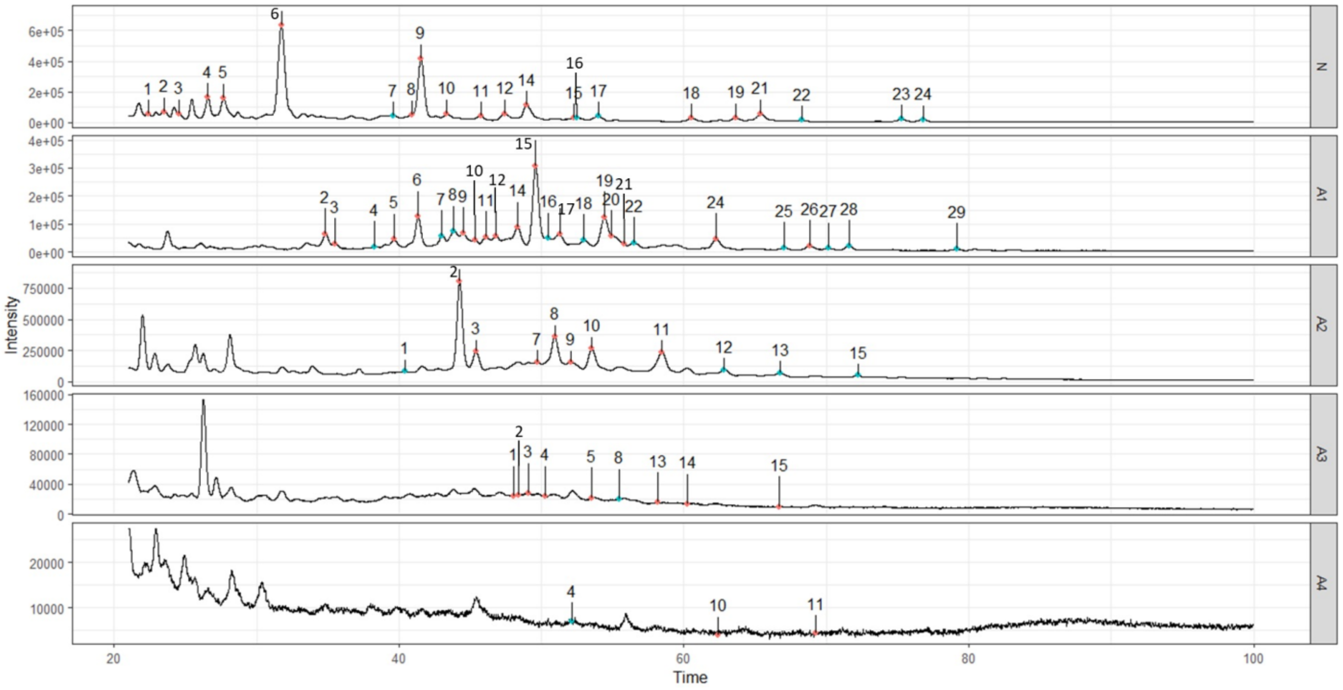

035

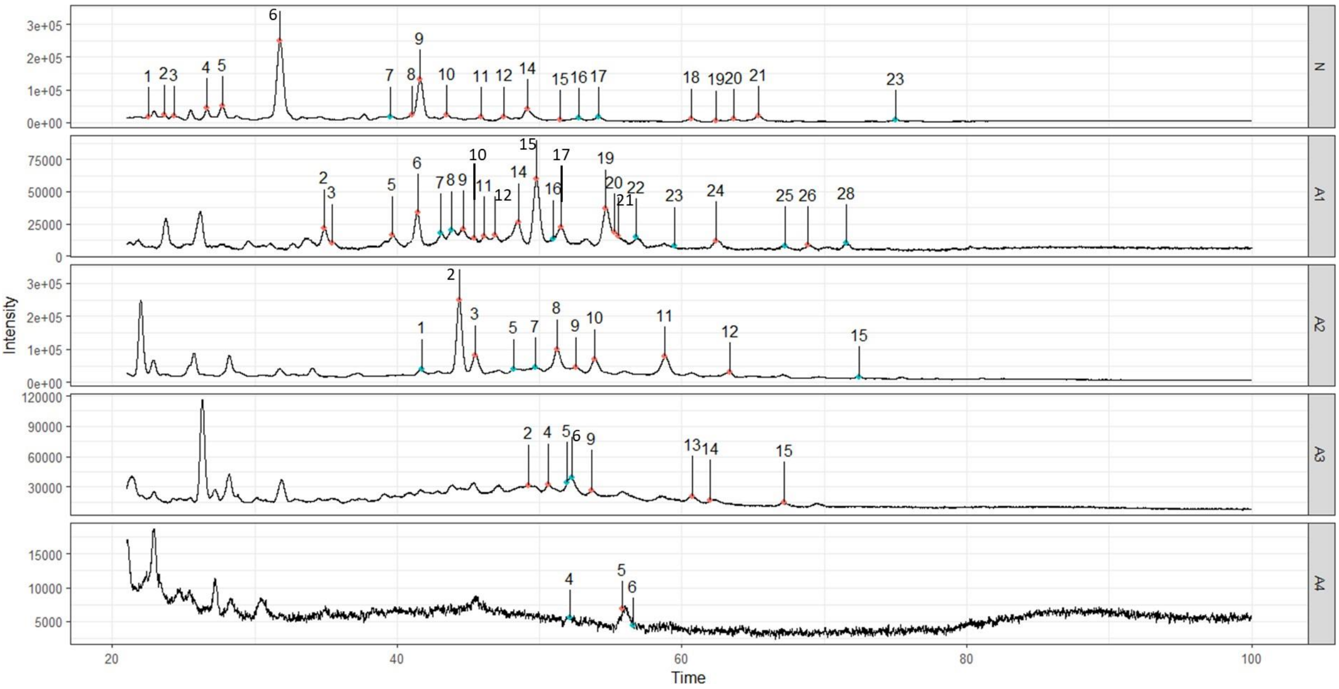

030

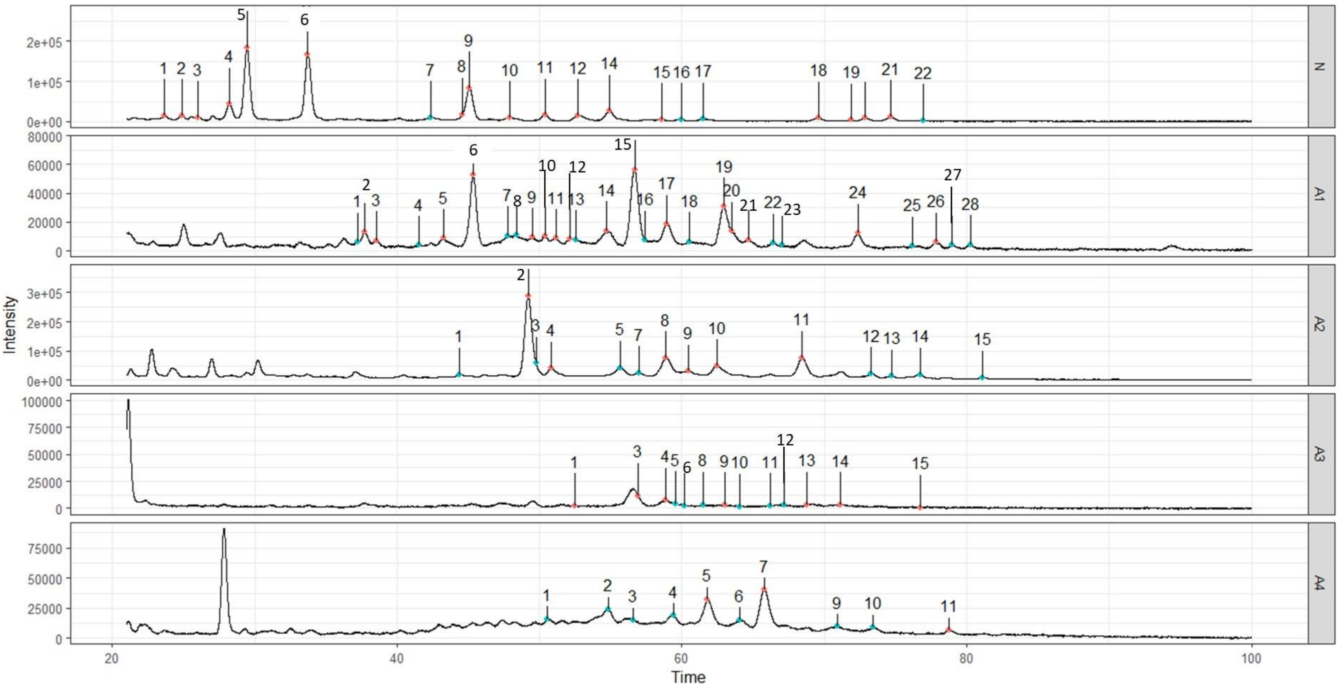

038

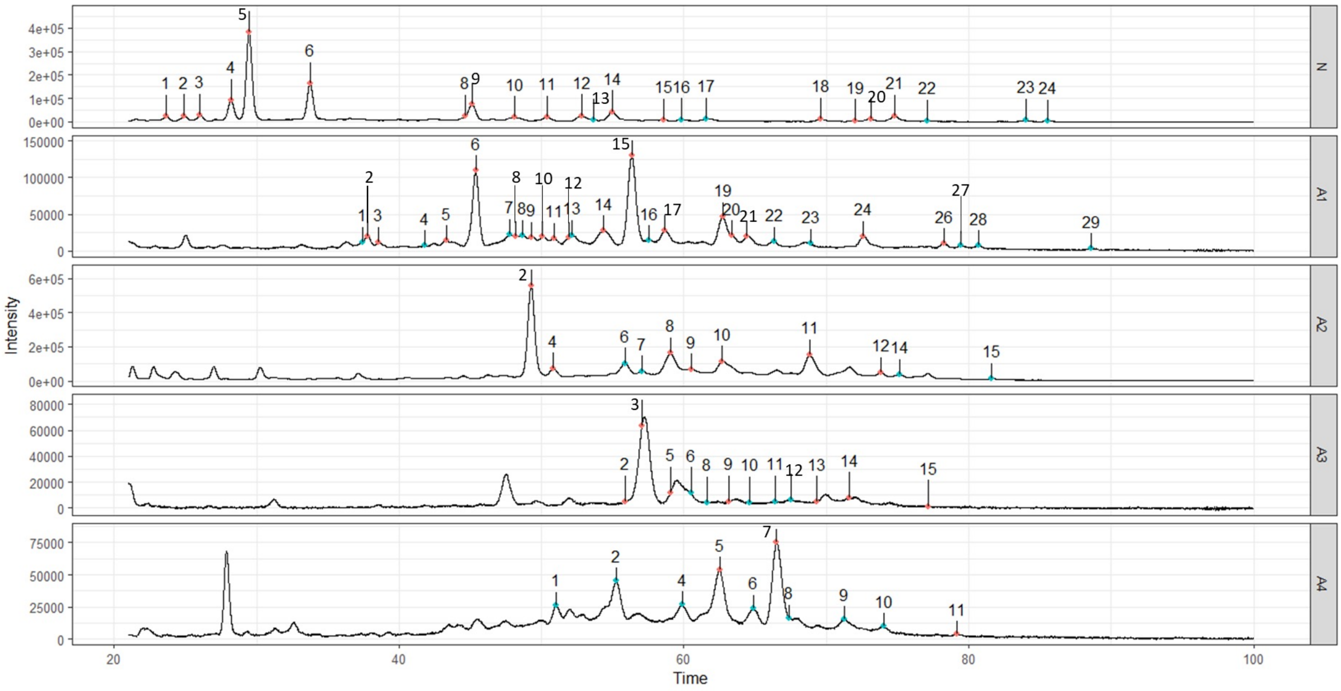

042

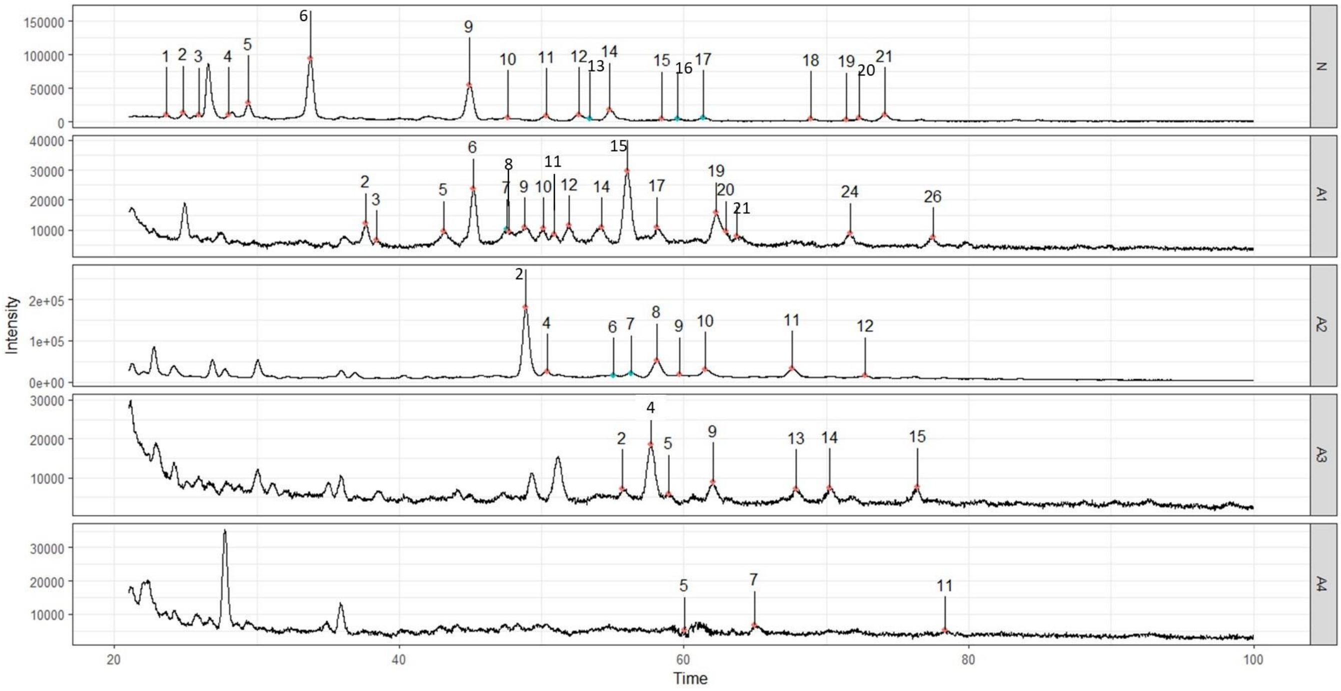

040

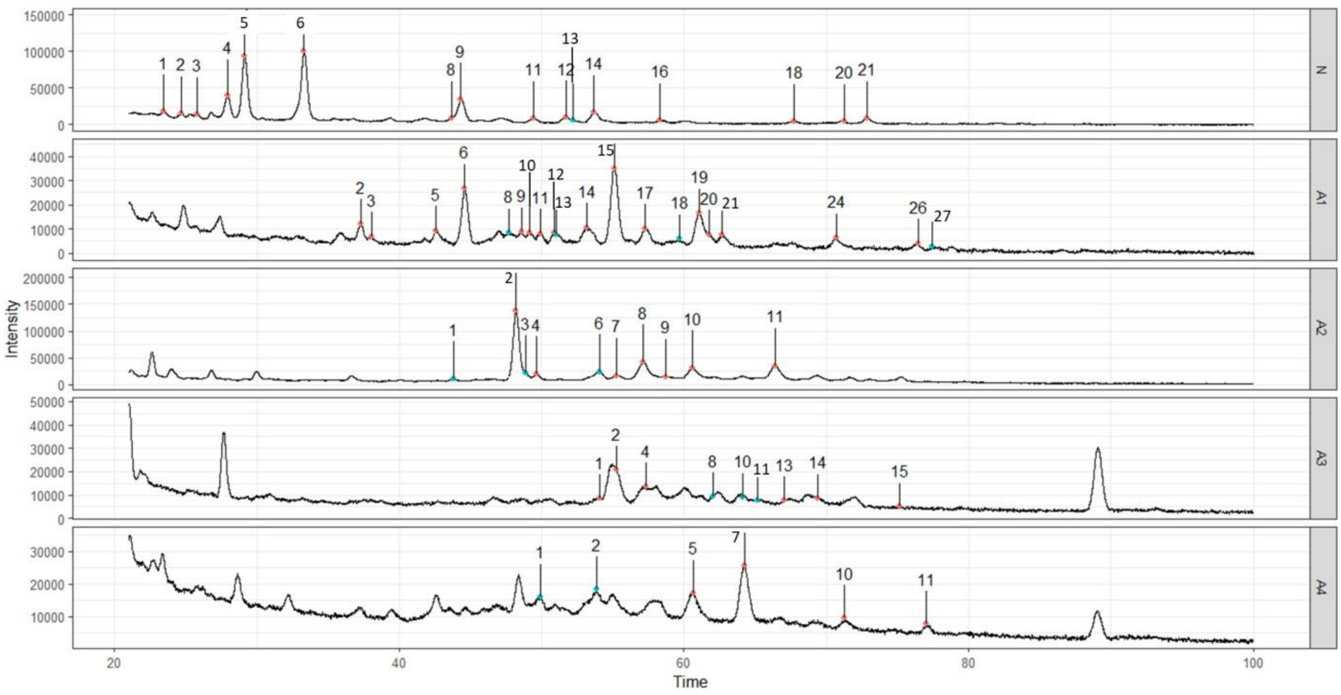

023

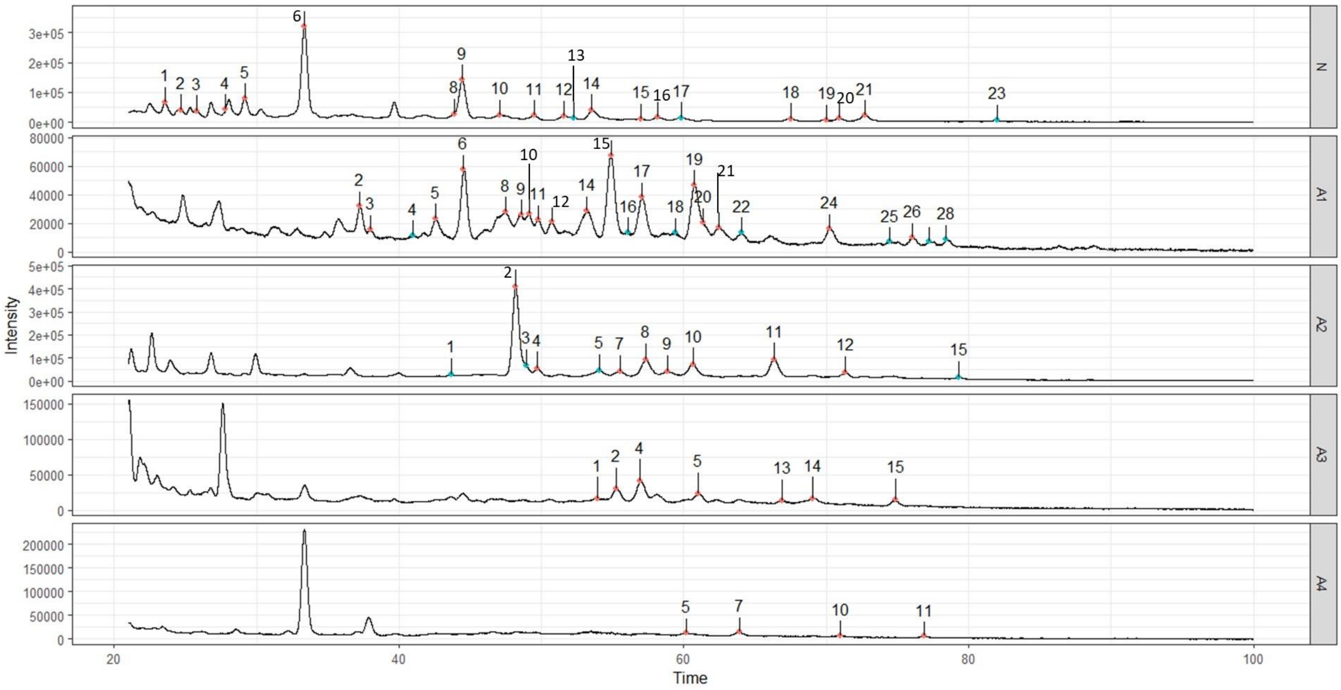

014

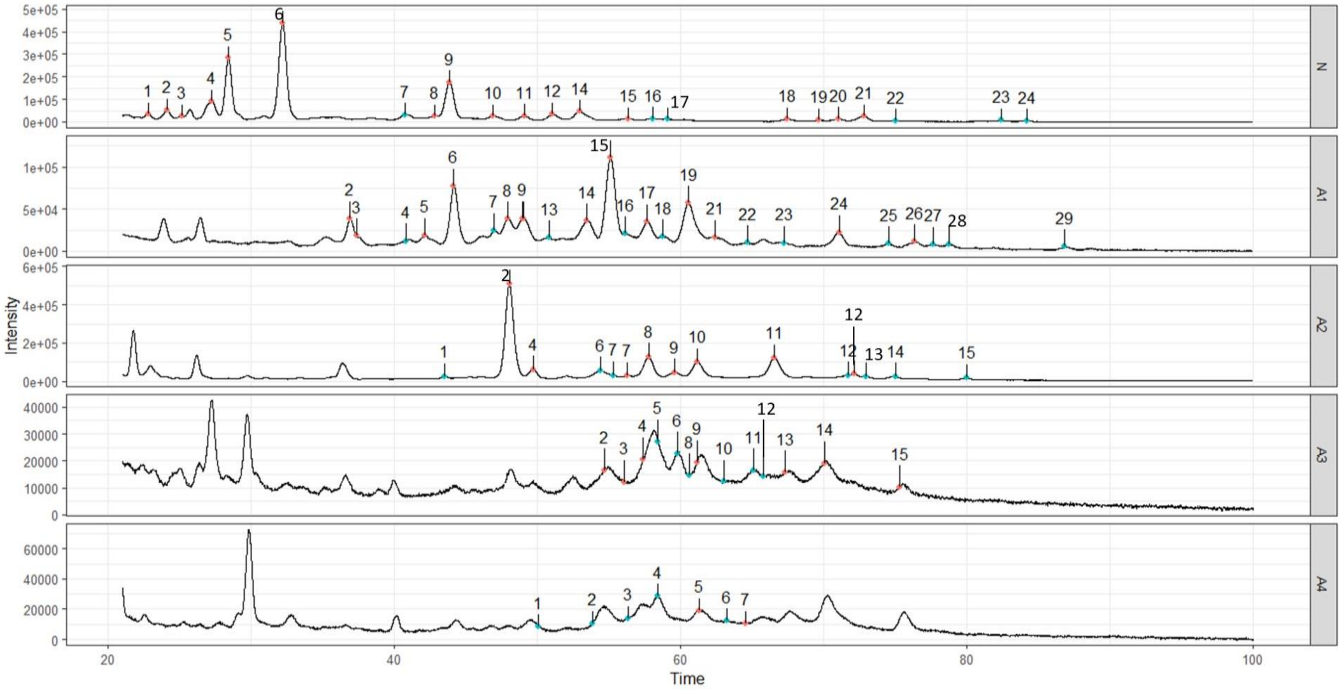

095

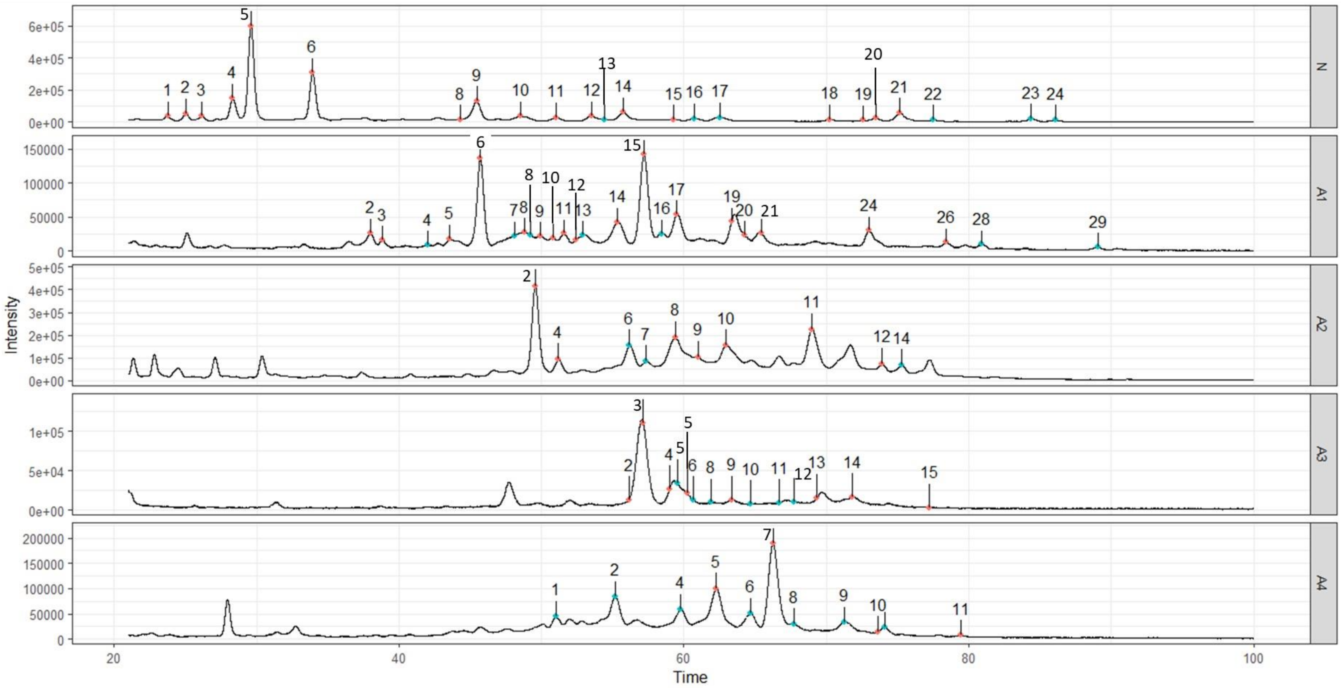

027

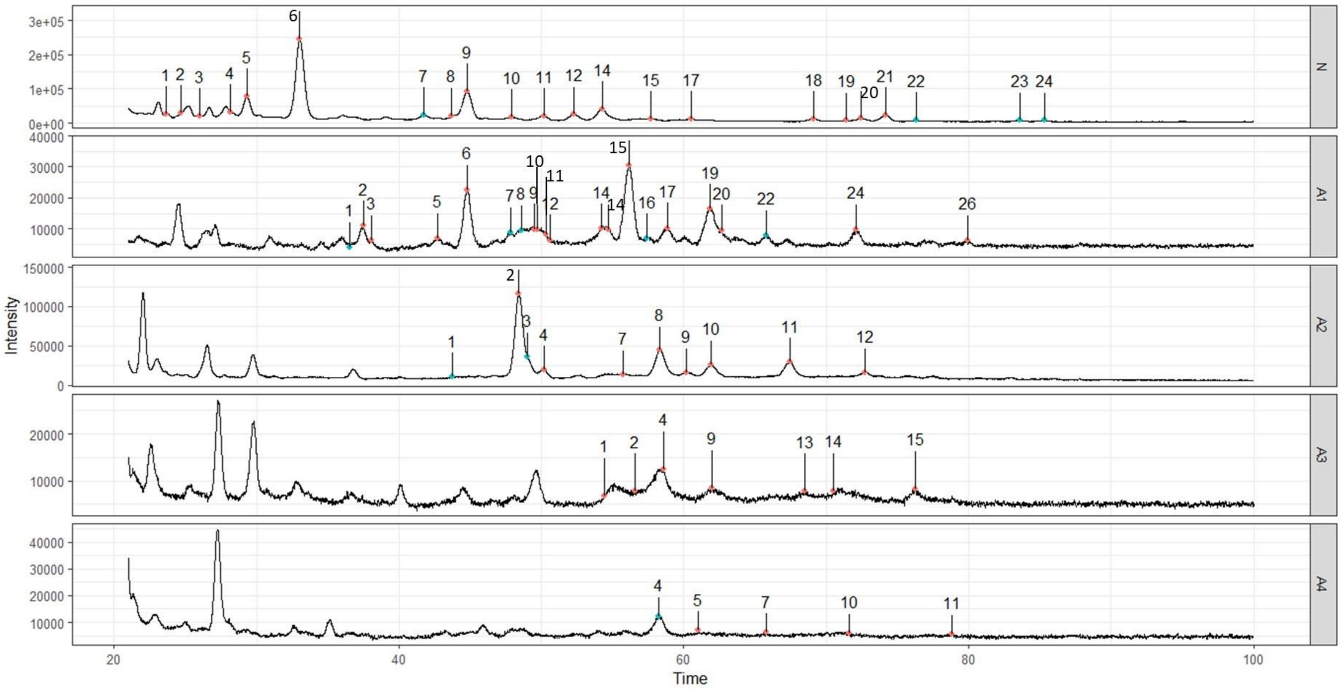

036

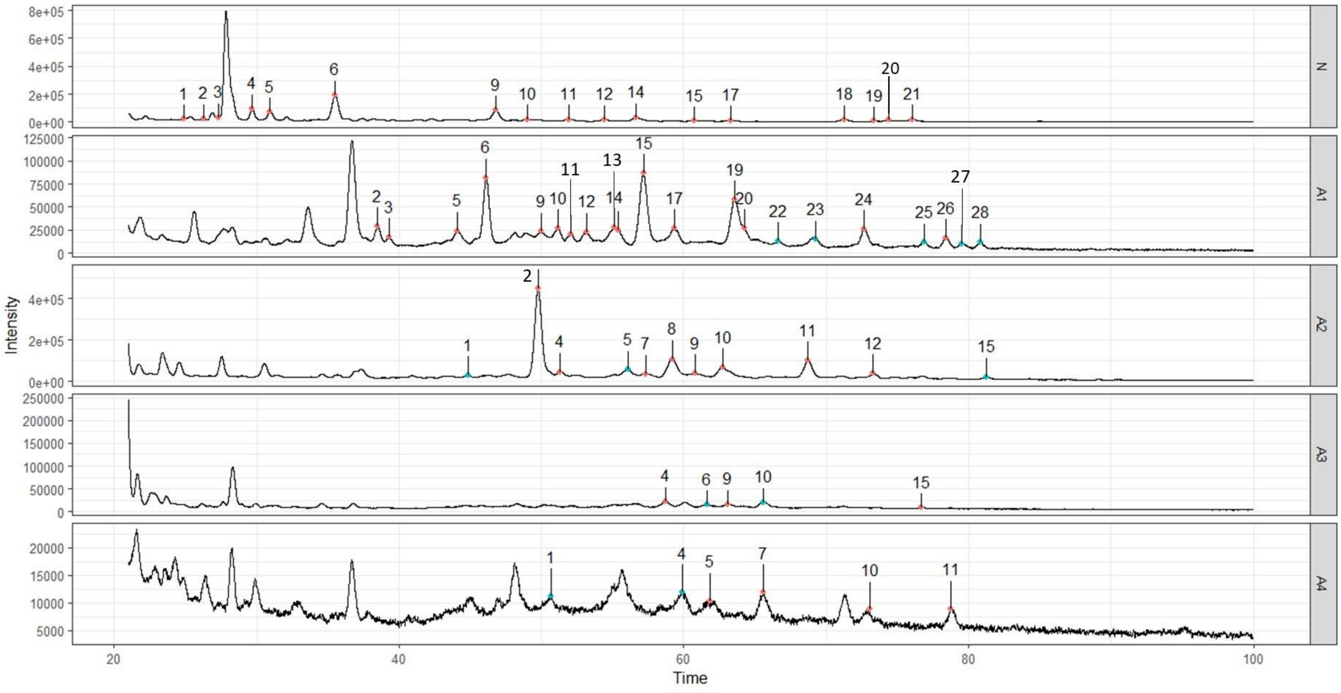

Supplement: Supplementary file 1 [file biomolecules-13-00756-s001.zip › suplementry data/Figure_S2.pdf]
