# Supplementary material for: Microheterogeneity and Individual Differences of Human Urinary N-Glycome under Normal Physiological Conditions
Source: Biomolecules. 2023 Apr 27;13(5):756. doi: 10.3390/biom13050756 (PMC10216293; doi:10.3390/biom13050756)
Supplement: Supplementary file 1 [file biomolecules-13-00756-s001.zip › suplementry data/Table_S1.pdf]

Table S1. Human urine sample information.

Sample symbol	Sex	Age
014	male	32
023	female	32
027	female	28
028	male	27
030	male	44
031	female	26
035	female	24
036	male	32
038	female	37
040	male	32
042	male	26
095	female	32
