# Supplementary material for: Microheterogeneity and Individual Differences of Human Urinary N-Glycome under Normal Physiological Conditions
Source: Biomolecules. 2023 Apr 27;13(5):756. doi: 10.3390/biom13050756 (PMC10216293; doi:10.3390/biom13050756)
Supplement: Supplementary file 1 [file biomolecules-13-00756-s001.zip › suplementry data/Table_S3.pdf]

Table S3. List of 24 unique serum N -glycans

NO.	Fraction of DEAE HPLC	Glucose unit on RP-HPLC	M/Z	ion form	MS	Composition[1]	Proposed structure[2]	Oxford notation of glycans[3]	The ratio of human serum N -glycans
1	N	6.67	1062.39	$2H^+$	2122.76	H10Hn2PA1	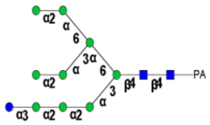	M9Gu	0.13%
2	N	6.89	758.79	$2H^+$	1515.57	H5Hn3PA1	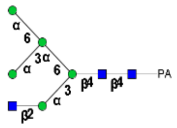	M5[3]A1	0.16%
3	N	15.06	880.85	$2H^+$	1759.68	H4Hn5PA1	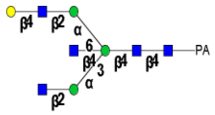	A2B[6]G(4)1	0.35%
4	A1	13.42	1026.39	$2H^+$	2050.77	N1H4Hn5PA1	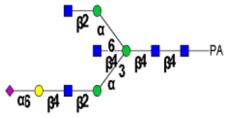	A2B[3]G(4)1S(6)1	1.12%
5	A1	15.86	1078.91	$2H^+$	2155.8	N1H5Hn4dH1PA1	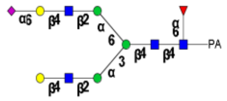	A2G(4)2[6]S(6)1	0.38%
6	A1	23.65	1261.48	$2H^+$	2520.94	N1H6Hn5dH1PA1	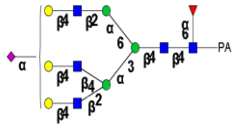	F(6)A3G(4)3S1	0.29%
7	A1	24.95	909.01	$3H^+$	2724.02	N1H6Hn6dH1PA1	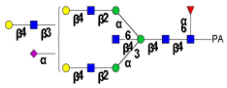	F(6)A2BG(4)2 LacNac1S1	0.15%
8	A2	10.55	1011.38	$3H^+$	3031.11	N2H7Hn6PA1	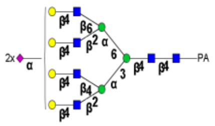	A4G(4)4S2	0.77%
9	A2	13.02	1407.02	$2H^+$	2812.03	N2H6Hn5dH1PA1	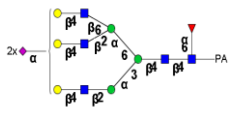	F(6)A3G(4)3S2	1.21%
10	A2	18.39	1333.99	$2H^+$	2665.97	N2H6Hn5PA1	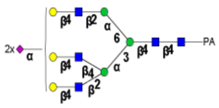	A3G(4)3S2	0.11%

11	A2	25.09	900.31	$2H^+$	1798.61	S1H5Hn4PA1	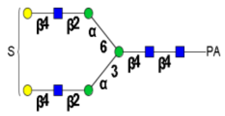	A2G(4)2Slf1	0.26%
12	A3	10.44	1157.09	$3H^+$	3468.26	N3H7Hn6dH1PA1	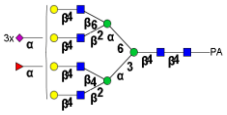	A4F1G(4)4S3	0.42%
13	A3	11.28	1108.41	$3H^+$	3322.2	N3H7Hn6PA1	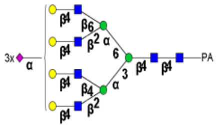	A4G(4)4S3	0.84%
14	A3	11.55	1108.41	$3H^+$	3322.2	N3H7Hn6PA1	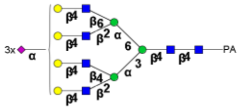	A4G(4)4S3	0.79%
15	A3	11.78	1108.41	$3H^+$	3322.2	N3H7Hn6PA1	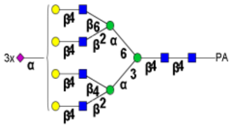	A4G(4)4S3	0.42%
16	A3	12.19	1108.41	$3H^+$	3322.2	N3H7Hn6PA1	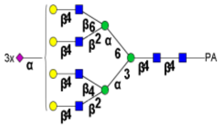	A4G(4)4S3	0.21%
17	A3	17.62	1479.54	$2H^+$	2957.07	N3H6Hn5PA1	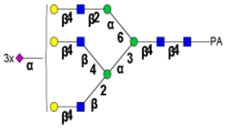	A3G(4)3S3	0.47%
18	A3	18.57	1084.07	$3H^+$	3249.18	N3H6Hn5dH2PA1	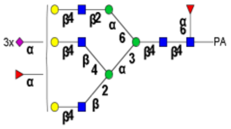	F(6)A3F1G(4)3S3	0.51%
19	A4	10.18	1302.81	$3H^+$	3905.41	N4H7Hn6dH2PA1	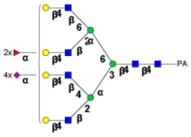	A4F2G(4)4S4	0.15%
20	A4	10.63	1254.13	$3H^+$	3759.35	N4H7Hn6dH1PA1	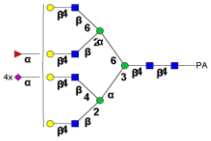	A4F1G(4)4S4	0.26%
21	A4	11.02	1254.13	$3H^+$	3759.35	N4H7Hn6dH1PA1	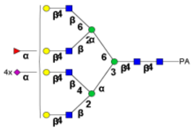	A4F1G(4)4S4	0.55%

22	A4	11.47	1205.44	$3H^+$	3613.3	N4H7Hn6PA1	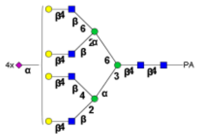	A4G(4)4S4	0.23%
23	A4	11.91	1205.44	$3H^+$	3613.3	N4H7Hn6PA1	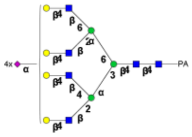	A4G(4)4S4	0.54%
24	A2	14.20	1264.46	$2H^+$	2526.86	S1N2H5Hn4dH1PA1	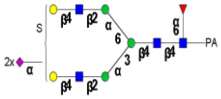	F(6)A2G(4)2 S2Slf1	0.22%

1. Abbreviations of the composition are N for NeuAc, H for hexose, Hn for N -acetylhexosamine, dH for deoxyhexose(fucose), S for sulfate, PA for pyridylamino residue.

2. Proposed Glycan structure. Symbols was explained as follows: PA: pyridylamino- or 2-aminopyridine; blue square: GlcNAc; yellow square: GalNAc; green circle: mannose; yellow circle: galactose; red triangle: fucose; purple diamond:sialic acid (NeuAc); blue circle: Glucose.
S: sulfur. GlycoWork-bench software was used to draw the glycan structure and their linkage information.

2. Abbreviations of the Oxford notation are M for Mannose, A for the number of antennae (GlcNAc) on the trimannosyl core , B for Bisecting GlcNAc, F for fucose(F at the start of the abbreviation indicates a core fucose), G for galactose, S for sialic acid, Gu for Glucose, Slf for Sulfate.
- free numbers after certain glycan element e.g. A2 are used to indicate the number of that element in glycan structure.
- [3]or [6] e.g. [6]G1 indicate that the glycan element comes after is on the antenna of the α1-3 or α1-6 mannose.
- Numbers in brackets e.g. F(6) are used to indicate linkages, where known.
